# Supplementary material for: Long-term prognosis after acute kidney injury (AKI): what is the role of baseline kidney function and recovery? A systematic review
Source: BMJ Open. 2015 Jan 6;5(1):e006497. doi: 10.1136/bmjopen-2014-006497 (PMC4289733; doi:10.1136/bmjopen-2014-006497)
Supplement: Author's manuscript [file bmjopen-2014-006497.draft_revisions.pdf]

Long Term Prognosis after Acute Kidney Injury (AKI) - What is the Role of Baseline Kidney Function and Recovery? A Systematic Review

Journal:	*BMJ Open*
Manuscript ID:	bmjopen-2014-006497
Article Type:	Research
Date Submitted by the Author:	02-Sep-2014
Complete List of Authors:	Sawhney, Simon; University of Aberdeen, Division of Applied Health Sciences; Aberdeen Royal Infirmary, Renal Unit, Ward 108<br>Mitchell, Mhairi; University of Aberdeen, Division of Applied Health Sciences<br>Fluck, Nick; Aberdeen Royal Infirmary, Renal Unit, Ward 108<br>Marks, Angharad; University of Aberdeen, Division of Applied Health Sciences; Aberdeen Royal Infirmary, Renal Unit, Ward 108<br>Black, Corrinda; University of Aberdeen, Division of Applied Health Sciences
**Primary Subject Heading**:	Epidemiology
Secondary Subject Heading:	Renal medicine
Keywords:	EPIDEMIOLOGY, Acute renal failure < NEPHROLOGY, Chronic renal failure < NEPHROLOGY, PUBLIC HEALTH

SCHOLARONE™ Manuscripts

For peer review only

Long Term Prognosis after Acute Kidney Injury (AKI) - What is the Role of Baseline Kidney Function and Recovery? A Systematic Review

Simon Sawhney[1,2*] MBChB
simon.sawhney@abdn.ac.uk

Mhairi Mitchell[1] MBChB
m.mitchell.08@aberdeen.ac.uk

Angharad Marks[1,2] MBBCh
a.marks@abdn.ac.uk

Nick Fluck[2] MBBS
nfluck@nhs.net

Corrinda Black[1] MBChB
corri.black@abdn.ac.uk

[1]Division of Applied Health Sciences, University of Aberdeen
[2]Renal Unit,Ward 108 Aberdeen Royal Infirmary

[*]Correspondence to:
Simon Sawhney, Division of Applied Health Sciences, University of Aberdeen, Polwarth Building, Foresterhill, Aberdeen AB25 2ZD, UK

Key words:
Acute Kidney Injury
Chronic Renal Insufficiency
Epidemiology
Systematic Review

Word count: 2591 words

For peer review only

For peer review only - http://bmjopen.bmj.com/site/about/guidelines.xhtml

ABSTRACT

Objectives: To summarise the evidence from studies of acute kidney injury (AKI) with regard to the effect of level of pre-AKI renal function and post-AKI renal function recovery on long-term mortality and renal outcomes, to assess whether these factors should be taken into account in future prognostic studies.

Design/Setting: A systematic review of observational studies listed in Medline and EMBASE from 1990 to present.

Participants: All AKI studies in adults with data on baseline kidney function to identify AKI; with outcomes either stratified by pre-AKI and / or post-AKI kidney function, or described by the timing of the outcomes.

Outcomes: Long-term mortality and worsening chronic kidney disease (CKD).

Results: Of 7385 citations, 16 studies of mortality and 5 studies of CKD outcomes met inclusion quality criteria. Three studies of mortality stratified by pre-AKI and six by post-AKI renal function. Two studies of CKD stratified by pre-AKI and two by post AKI function. The presence of CKD pre-AKI (compared to AKI alone) was associated with doubling of mortality and a four- to five-fold increase in CKD outcomes. Non-recovery of kidney function was associated with greater mortality and CKD outcomes in some studies, but findings were inconsistent varying with study design. Two studies reported that risk of poor outcome reduced over time post-AKI. Meta-analysis was precluded by variations in definitions for AKI, CKD and recovery.

Conclusions: While carefully considered in clinical practice, few heterogeneous long-term prognostic studies address pre-AKI and post-AKI renal function. Variable AKI prognosis may in part be explained by these factors. Future studies should incorporate pre-AKI and post-AKI measurements with consistent definitions to establish how to predict future risk following AKI.

For peer review only

For peer review only - http://bmjopen.bmj.com/site/about/guidelines.xhtml

Strengths and Limitations of this study

This systematic review followed strict inclusion, exclusion and quality assessment criteria to summarise the available evidence regarding the role of pre-AKI baseline and post-AKI recovery of renal function in long-term AKI outcomes.

Few studies reported long-term AKI outcomes stratified by pre-AKI and post-AKI factors. Quantitative meta-analysis was precluded by heterogeneity in design of included studies.

For peer review only

For peer review only - http://bmjopen.bmj.com/site/about/guidelines.xhtml

INTRODUCTION

Acute kidney injury (AKI) affects an estimated 13-18% of hospitalised patients[1], frequently under the care of specialties other than nephrology. With the advent of an internationally agreed definition based on changes in serum creatinine and urine output[3], there is now increasing awareness of the poor outcomes suffered by such patients and this has been accompanied by an emphasis on screening in an effort to improve patient safety and outcomes[1]. Some of the poor outcomes (including increased mortality[3,4] and development of chronic kidney disease (CKD)[5-7]) are increasingly described after hospital discharge, but this is variable and factors associated with long-term prognosis are poorly understood[1]. Recent guidelines from the National Institute of Clinical Excellence (NICE) call for more studies into acute kidney injury, with non-AKI comparators, but without specifying which factors should be included in study design.

AKI occurs in many different situations and the context is likely to be important both when studying onset of AKI and assessing future outcome risk. In particular, clear knowledge of prior kidney (*pre-AKI* baseline) function is essential to distinguish AKI from CKD. This "baseline function" is typically a creatinine measurement prior to hospitalisation, but may not be available or may be estimated if absent. Baseline function is required for grading AKI severity; as a reference for establishing if recovery is complete; and as a means of stratifying patients with and without *pre-AKI* CKD. It is intuitive that AKI in patients with advanced baseline *pre-AKI* CKD could present and behave differently[8], thus studies without baseline function are at risk of selection bias and misclassification of CKD as AKI.

After an episode of AKI (for instance at hospital discharge or clinic review), the physician also has an opportunity to consider a patient's most recent *post-AKI* kidney function. Accounting for *post-AKI* recovery from AKI (e.g. return of creatinine to within 20% pre-AKI creatinine[3]) may assist future risk assessment. However, the timing of this *post-AKI* assessment may also influence its predictive ability, since clinical course may vary, with both recovery and deterioration possible and not necessarily at a constant rate[9].

Thus, while there is evidence that AKI may have a poor overall prognosis, in this systematic review we seek to evaluate whether previous observational studies have demonstrated the modifying contribution of *pre-AKI* baseline function, *post-AKI* recovery and the timing of outcomes. We also assess whether stratification by these factors should be necessary in future prognostic studies and might provide valuable information to the physician making a risk assessment.

METHODS

A systematic review of observational studies was undertaken in accordance with guidelines for meta-analysis of observational studies in epidemiology (MOOSE)[10]. Medline and EMBASE were searched from January 1990 through October 2012 using Medline subject heading (MeSH) terms and free text for acute kidney injury (acute renal failure, acute kidney disease, acute kidney injury, acute dialysis, RIFLE, AKIN) and prognosis (prognosis, survival, mortality, follow up, progression, chronic kidney disease, renal replacement therapy, chronic dialysis, end-stage kidney disease) (see

For peer review only - http://bmjopen.bmj.com/site/about/guidelines.xhtml

supplementary material). Reference lists of relevant studies and review articles were also searched. There were no language restrictions.

Studies were included if AKI was defined with: creatinine changes within a specified time interval, hospital episode coding for AKI, or the initiation of acute renal replacement therapy (RRT). Studies had to report either mortality or a CKD outcome (development of CKD, progression of CKD severity, or end-stage kidney disease (ESKD)). Studies were included that reported adults (age over 18 years), had at least 50 participants with AKI surviving to hospital discharge, and had follow-up of at least one year. Studies of those with only specialised conditions (e.g. cancer, chemotherapy, transplantation) were excluded.

Two reviewers independently screened titles, abstracts and full papers against the inclusion and exclusion criteria. Where disagreement could not be resolved by discussion, a third author was available, but was not required. One researcher extracted information from the included studies into a data extraction proforma, with confirmation by a second reviewer.

Quality Assessment
Six quality criteria (based on guidelines for assessing the quality of prognostic studies[11]) with a specific focus on the key areas of potential bias and heterogeneity in studies identifying AKI and subsequent outcomes, were used to assess the included studies (figure 1).

Analysis
Study characteristics and quality assessment information were tabulated and described. Multiple papers from the same study dataset were presented together with findings reported from the most complete paper.

Quality criteria 1-3 limited analysis to studies that accurately identified AKI patients and restricted to those who survived the acute event, without significant potential for misclassification of CKD patients. If criteria 1-3 were not met, the study was excluded from further analysis.

Further analysis was limited to studies that analysed patients separately according to one of pre-AKI baseline, post-AKI recovery, or the timing of outcomes by (quality criteria 4-6).

The remaining studies, satisfying criteria 1-3 and one of 4-6, were then summarised in two tables for mortality and CKD outcomes. Clinical setting of AKI was described for each table as follows: intensive care (ITU: patients admitted to ITU, regardless of RRT use); postoperative; cardiac (patients presenting following a myocardial infarction or coronary angiogram); unselected (studies of hospital admissions where the clinical settings above did not describe all patients). Retrospective or prospective design was also reported.

Mortality and CKD outcomes were presented separately, reporting study characteristics, population, exposure, comparators, follow-up and measure of outcome. Multivariate adjusted risk ratios for both survival and CKD outcomes were

For peer review only - http://bmjopen.bmj.com/site/about/guidelines.xhtml

displayed graphically if available. If the timing of outcome was considered in a study this was indicated and described.

RESULTS

The literature search identified 7385 citations. Following review of titles, abstracts and full texts (figure 2), 68 papers describing 61 unique studies were identified (further details summarised in supplementary material). Study size varied, ranging from 61 to 82711 individuals with follow-up from 12 to 142 months. 30 out of 61 studies satisfied quality criteria 1-3, reported either mortality or CKD outcomes (and these studies are summarised in table 1)[12-40]. Notably, 28/61 studies had insufficient pre-AKI data to avoid misclassification of CKD as AKI. Of 30 remaining studies stratification or separation by exclusion of pre-AKI or post-AKI function subgroups was performed in 16/30 mortality studies with 14/30 not conducting separate analyses. It was also performed in 5/30 CKD outcome studies with 25/30 not conducting separate analyses. As noted in table 2, most studies satisfied quality criteria 1-3 by excluding patient groups rather than by stratifying by pre-AKI or post-AKI function. Only three mortality studies stratified by pre-AKI function and six by post-AKI function. Two CKD studies stratified by pre-AKI and two post AKI function. Only two mortality studies[9,20] and one CKD outcome study[9] addressed whether the risk of outcomes changed with increasing time from AKI.

Among these studies, definitions varied: AKI was defined by acute RRT[14], different thresholds for creatinine rise[16,24-27], or hospital episode code[15,19]. CKD was defined by hospital codes[19], low eGFR for CKD cut off (<45ml/min/1.73m^2)[14,21] conventional eGFR for CKD (<60 ml/min/1.73m^2) [16], varying thresholds based on serum creatinine[22,36] or hospital episode code[19]. Definitions of renal recovery also varied: timing from three days[27] to three months[16]; and extent from to within 50% of baseline[21] to within 10% of baseline[15], or below a pre-AKI value[25].

For peer review only

For peer review only - http://bmjopen.bmj.com/site/about/guidelines.xhtml

Table 1- Description and Application of Quality Criteria 4-6 in Studies Satisfying Criteria 1-3

			Mortality outcomes reported		CKD outcomes reported		For either outcome
Paper	N	Clinical Setting	by Pre-AKI baseline	by Post-AKI recovery	by Pre-AKI function	by Post-AKI recovery	Timing considered
Hoste 2011 [28]	82	ITU	N	N	-	-	-
Lopes 2010 [29]	61	ITU	N	N	-	-	-
Manns 2003 [30]	66	ITU	N	N	-	-	-
Schiffl 2008 [12]	226	ITU	CKD excluded	Y	-	-	-
Triverio 2009 [18]	95	ITU	Y	Y	-	-	-
Bucaloiu 2012 [13]	1610	Unselected	CKD excluded	Recovered only	CKD excluded	Recovered only	-
Hsu 2009 [14]	782	Unselected	CKD only	N	N	N	-
Ishani 2009 [19]	7197	Unselected	Y by code alone	N	Y by code alone	N	-
Jones 2012 [15]	719	Unselected	CKD excluded	Recovered only	CKD excluded	Recovered only	-
Lafrance 2010 [20]	82711	Unselected	Y	Y	-	-	Sensitivity analysis excluding first six months
Lo 2009 [31]	343	Unselected	N (excluded only if eGFR<45)	N	-	-	-
Ng 2011 [32]	262	Unselected	N	N	-	-	-
Ponte 2008 [22]	177	Unselected	N (Cr>1.4 excluded)	Y	-	-	-
Wald 2012 [33]	41327	Unselected	N	N	N	N	-
Gupta 2005 [34]	143	Cardiac	N	N	-	-	-
Kimura 2010 [35]	81	Cardiac	N	N	-	-	-
Lindsay 2003 [36]	179	Cardiac	Y	N	-	-	-
Maioli 2012 [16]	167	Cardiac	CKD only	Y	-	-	-
Rihal 2002 [37]	185	Cardiac	N	N	-	-	-
Roghi 2008 [38]	106	Cardiac	N	N	-	-	-
Brown 2010 [23]	1886	Postoperative	N	Y	-	-	-
Coca 2010 [24]	6257	Postoperative	N	Y	-	-	-
Ishani 2011 [9]	4053*	Postoperative	N	N	N	N	Changing hazard ratios for mortality and CKD outcomes in time intervals
Kheterpal 2007 [17]	101	Postoperative	CKD excluded	N	-	-	-
Loef 2005 [25]	145	Postoperative	N	Y	-	-	-
Luckraz 2005 [39]	53	Postoperative	N	N	-	-	-
Mehta 2010 [26]	2083	Postoperative	N (excluded only Cr>2)	Y	-	-	-
Swaminathan 2010 [40]	1113	Postoperative	N	N[$]	-	-	-
Van Kuijk 2010 [27]	120	Postoperative	N	N	CKD excluded	Y	-
Wu 2011 [21]	4393	Postoperative	Y by GFR>/<45	Y	Y by GFR>/<45	Y	-

N = outcome addressed but not per quality criterion, - = outcome not addressed by study, * = 19779 in total, 4053 of which had >50% cr rise, [$] = outcomes by rate of fall of creatinine over a 24 hours rather than a recovery variable, Cr = creatinine

For peer review only - http://bmjopen.bmj.com/site/about/guidelines.xhtml

Table 2 –Studies of Mortality Outcomes in AKI by pre and post-AKI Kidney Function

Mortality	N	Clinical Setting	Design	AKI Exposure	Comparator	Follow Up (*mean /median)	Pre-AKI baseline separation	Post-AKI recovery separation	Recovery definition	Findings (*statistically significant p<0.05)
Schiffl 2008 [12]	226	ITU	Cohort	Acute RRT	None	5 years	CKD excluded	Y	Within 10% baseline	Mortality 83% without recovery, 33% with recovery<br>HR without vs with recovery 4.1* (no non-AKI comparator)
Triverio 2009 [18]	95	ITU	Cohort	Acute RRT	None	3 years	Y	Y	eGFR>60	Mortality 50% if baseline CKD, 29% if eGFR<60 at discharge, 18% if no renal impairment before or after AKI
Bucaloiu 2012 [13]	1610	Unselected	Cohort	Cr rise >50%	No AKI	*3.3 years	CKD excluded	Recovered only	Within 10% baseline	HR Mortality 1.48*
Hsu 2009 [14]	782	Unselected	Cohort	Acute RRT	CKD with no RRT	4 years	CKD only (eGFR<45)	N	-	HR composite endpoint of ESKD or Mortality 1.3*
Ishani 2009 [19]	7197	Unselected	Cohort	Code	No AKI or CKD code	2 years	Y by code alone	N	-	HR Mortality vs no AKI or CKD<br>AKI and CKD 3.24*, AKI 2.48*, CKD 1.45*
Jones 2012 [15]	719	Unselected	Cohort	Code	No AKI	*2.5 years	CKD excluded	Recovered only	Within 10% Baseline	HR Mortality 1.08
Lafrance 2010 [20]	82711	Unselected	Cohort	Cr rise >50%	No AKI	*2.34 years	Y	Recovered only in a subanalysis	Within 10% baseline	HR Mortality for AKI vs no AKI in 90d survivors 1.41*<br>Subgroup of 6 month survivors 1.13*<br>Subgroup with recovery 1.47*
Ponte 2008 [22]	177	Unselected	Cohort	Cr rise from <1.4mg/dl to >2	None	*7.2 years	CKD excluded (Cr>1.4mg/dl)	Y	Cr <1.4mg/dl	10 year mortality 40% with recovery, 57% without recovery
Lindsay 2003 [36]	179	Cardiac	Cohort	Cr rise 50% from <1.2mg/dl	No AKI	1 year	CKD excluded (cr>1.2mg/dl)	N	-	1year mortality 9.5% AKI, 2.7% no-AKI
Maioli 2012 [16]	167	Cardiac	Cohort	Cr rise (0.5mg/dl by 3 days)	No AKI	*3.8 years	CKD only (eGFR<60)	Y	Within 25% baseline at 3 months	HR Mortality for AKI with recovery1.3*, without recovery 2.3*
Brown 2010 [23]	1886	Postoperative	Cohort	Cr rise 0.3mg/dl or 50%	No AKI	*2.6 years	N	Y	Number of days AKI definition met	HR Mortality for AKI vs no AKI by AKI Duration<br>1-2d 1.51*, 3-6d 1.74*, >7d 3.45*, persistent 5.75*
Coca 2010 [24]	6257	Postoperative	Cohort	Cr rise 0.3mg/dl or 50%	No AKI	*3.8 years	N	Y	Number of days AKI definition met	HR Mortality for AKI vs no AKI by AKI Duration<br><2d 1.15*, 3-6d 1.5*,>7d any duration with RRT 2.10*
Kheterpal 2007 [17]	101	Postoperative	Cohort	Cr clearance fall to <50	No AKI	1 year	CKD excluded	N	-	1 year mortality 12% AKI, 9% no AKI
Loef 2005 [25]	145	Postoperative	Cohort	Cr rise 25%	No AKI	100 months	N	Y	"improved to or below the preoperative level"	HR Mortality for AKI 1.63*, AKI with recovery 1.66*, AKI without recovery 1.72*, Non-recovery vs recovery 1.22 (not significant)
Mehta 2010 [26]	2083	Postoperative	Cohort	Cr rise 50% or 0.7mg/dl	No AKI	*7 years	N	Y	AKI definition no longer met at 7 days	HR mortality for AKI with recovery 1.21*, without recovery 1.47*
Wu 2011 [21]	4393	Postoperative	Cohort	Cr rise >50%	No AKI and no CKD	*4.76 years	Y by GFR>/<45	Y	Within 50% baseline	HR Mortality for AKI only 1.94*, CKD only 2.64*, AKI and CKD 3.28*<br><br>Stratified by recovery<br>AKI and CKD with recovery 3.0*, without recovery 4.59*<br>AKI only with recovery 1.96*, without recovery 2.18 *<br>CKD without AKI 2.59*

N = outcome addressed but not per quality criterion, - = outcome not addressed by study, ITU=intensive therapy unit; RRT=renal replacement therapy; AKI=acute kidney injury; CKD=chronic kidney disease; eGFR=estimated glomerular filtration rate; HR=hazard ratios; ESKD=end-stage kidney disease (dialysis>90days); Cr=serum creatinine

For peer review only - http://bmjopen.bmj.com/site/about/guidelines.xhtml

Table 3 –Studies of CKD Outcomes in AKI by pre and post-AKI Kidney Function

Progression	N	Clinical Setting	Design	AKI Exposure	Comparator	Follow-up (*mean /median)	Pre-AKI baseline separation	Post-AKI recovery separation	Recovery definition (discharge unless otherwise stated)	Findings (*statistically significant p<0.05)
Bucaloiu 2012 [13]	1610	Unselected	Cohort	Cr rise >50%	No AKI	*3.3 years	CKD excluded	Recovered only	Within 10% baseline	Development of New CKD Stage 3 per 1000 person-year<br>28.1 AKI vs 13.1 no AKI (HR 1.91*)
Ishani 2009 [19]	7197	Unselected	Cohort	Code	No AKI or CKD code	2 years	Y by code alone	N	-	ESKD/1000 person-year (HR vs no code)<br>AKI and CKD 79.45 (HR 41.2*)<br>AKI 24.52 (HR 13*)<br>CKD only 19.88 (HR 8.43*)<br>No Code 2.08 (reference)
Jones 2012 [15]	719	Unselected	Cohort	Code	No AKI	*2.5 years	CKD excluded	Recovered only	Within 10% Baseline	HR for new CKD Stage 3 (15% of AKI vs 3% of no AKI patients)<br>3.82*
van Kuijk 2010 [27]	493	Postoperative	Cohort	10% Cr change by day 2 post-op	No AKI	*5 years	CKD excluded	Y	Within 10% baseline at day 3	Development of New CKD<br>11% if no AKI, 32% if AKI recovered (HR 3.4*), 36% if AKI without recovery (HR3.6*)
Wu 2011 [21]	4393	Postoperative	Cohort	Cr rise (RIFLE, or 50% rise if previous CKD)	No AKI and no CKD	*4.76 years	Y by GFR>/<45	Y	Within 50% baseline	Development of ESKD (vs no code)<br>AKI only 4.64*, CKD only 40.86*, AKI and CKD 91.69*<br><br>Stratified by recovery<br>AKI + CKD + without recovery 212.73*<br>AKI + CKD + recovery 74.07*<br>AKI only + without recovery 60.95*<br>AKI only + recovery 4.5*

N = outcome addressed but not per quality criterion, - = outcome not addressed by study; ITU=intensive therapy unit; RRT=renal replacement therapy; AKI=acute kidney injury; CKD=chronic kidney disease; eGFR=estimated glomerular filtration rate; HR=hazard ratios; ESKD=end-stage renal disease (dialysis>90days); Cr=serum creatinine

For peer review only - http://bmjopen.bmj.com/site/about/guidelines.xhtml

Mortality findings

There were 16 mortality studies (table 2). Follow up ranged from 1 to 7 years and mortality up to 83% for AKI patients at 5 years. AKI was associated with increased mortality in all but one study[15] regardless of pre-AKI baseline (HR 1.08 to 4.59) (figure 3))or recovery of renal function (HR1.08 to 5.75) (figure 4). Five studies did not report non-AKI comparators[12,18,22] or risk ratios[17,36].

Of the three studies stratified by pre-AKI baseline function, in two the overall prognosis was worse in AKI patients with prior CKD with doubling of hazard ratios with respect to a non-AKI non-CKD comparator[16,21]. However, in a third study, where comparators were also stratified by eGFR, the independent mortality risk from AKI diminished with advancing CKD[20]. Six studies compared mortality stratifying either by post AKI recovery (four studies)[16,21, 25, 26] or by AKI duration (two studies)[23,24]. All but one[16] were postoperative studies with different thresholds for defining recovery. Risk from incomplete recovery varied from minimal with overlapping confidence intervals[16,21,25, 26] to greater than double when AKI lasted more than one week[23,24]. Only one study combined stratification by both pre-AKI baseline and post-AKI recovery (defining recovery to 50% baseline, CKD as eGFR<45 ml/min/1.73m^2) with both non-recovery and baseline CKD adding to mortality risk[21].

CKD Outcomes

In five studies, there was marked variation in CKD outcomes following AKI (HR1.91 to 213) depending on pre-AKI baseline and post-AKI recovery (table 3 and figure 5)[13,15,21,19,27]. In the two studies stratifying by pre-AKI function, AKI with baseline CKD was associated with a substantially greater (four-fivefold) risk of ESKD than AKI in cases without prior CKD[19,21] although notably one study defined CKD at eGFR <45ml/min/1.73m^2 [21] and the other by a hospital code[19]. The two studies stratifying by post-AKI function had different findings. In one postoperative study[27] there was no association between non-recovery post-AKI (vs patients with recovery) and additional CKD progression, when recovery was measured at day three[27], but association was substantial (five-tenfold) in the other study where recovery was defined at hospital discharge[21]. Again only one study stratified by both baseline and recovery, finding both associated with poorer prognosis[21].

Timing of Outcomes

Two studies considered whether hazard ratios changed over time following the AKI episode[9,20]. Lafrance et al. (2010)[20], performed a sensitivity analysis of their study of AKI survivors and mortality, limiting analysis from 90 day to six month AKI survivors. The mortality HR (AKI versus non-AKI) fell from 1.41(1.39-1.43) in 90 day survivors to 1.13 (1.11-1.14) in six month survivors[20]. Ishani et al. (2011)[9] reported changes in both hazard ratio of mortality and CKD outcomes from AKI (vs no AKI) when dividing follow up into six month time discrete intervals. For both mortality and CKD outcomes, greatest relative risk (vs non-AKI) was in the first six months, with marked attenuation of risk (but still statistically significant) from one year[9]. While recognising that risk may not be constant, the study did not also consider the importance of stratifying patients by pre-AKI baseline or post-AKI recovery.

DISCUSSION

The introduction of a global definition for AKI has led to growing interest in the high incidence and poor outcomes. In this review of 7385 citations, there were only 30

For peer review only - http://bmjopen.bmj.com/site/about/guidelines.xhtml

studies that met the initial quality criteria for selecting AKI patients with sufficient efforts to avoid misclassifying CKD. Of these studies only three mortality studies stratified by pre-AKI, six by post-AKI function, two CKD outcome studies by pre-AKI and two by post AKI function. Only one study considered both pre-AKI and post-AKI function. Only two studies considered the change in risk over time from AKI.

As with two previous reviews, we note that AKI is associated with overall poorer mortality and CKD outcomes[4,6], but with substantial variation in the magnitude of risk. We were transparent about the variation in definitions and clinical setting and identified heterogeneity as a problem with the design of AKI studies to date. The two previous reviews included studies with incomplete or estimated data on baseline kidney function[4,6], but in studies with available data we found that outcomes were modulated by both pre-AKI baseline and post-AKI renal recovery. Unfortunately, these studies varied in definitions for AKI, recovery and follow up preventing pooled meta-analysis. While proteinuria and oliguria may also be important factors, research on their association with long term prognosis is sparse and we therefore focused on the course of serum creatinine prior and subsequent to the AKI episode.

Pre-AKI baseline CKD was associated with doubling of mortality outcomes and four- to five-fold increased risk of CKD outcomes compared to non-AKI non-CKD patients. Unfortunately, only one study chose CKD comparators for AKI patients at each level of pre-AKI function, finding that the additional risk from AKI diminished in those already at high risk due to advanced baseline CKD[20]. It is likely the mortality and ESKD risk in advanced CKD is already high and less influenced by AKI.

Non-recovery of kidney function was associated with greater mortality and CKD outcomes in some studies, but the finding was inconsistent and dependent on study design. Studies with a less stringent threshold[21] and later point of assessment[16] showed a poorer prognosis with non-recovery, highlighting the significance of arbitrary thresholds in study design. We also note a further study outside the time period in this review reporting a cohort with poorer outcomes with non-recovery after AKI, in line with our overall findings[41].

Increased risk of poor outcomes following AKI may be greatest early post-AKI, and therefore the timing of clinical assessment is important. Despite this, most studies followed a “proportional hazards” assumption (i.e. that the relative risk of AKI vs non-AKI comparator was constant over time), but this may not hold. Only two studies considered the timing of assessment post-AKI and found that both additional risk of mortality and renal progression may diminish over time[9,20], but neither combined this with adjustment or stratification by post-AKI recovery. Both factors are required to assess if the rate of CKD progression is more rapid in AKI than non-AKI (but baseline CKD) comparators with equal kidney function at the time of hospital discharge. Thus, understanding of how mortality and renal progression risks change over time remains inadequately addressed.

Overall while our findings broadly agree with previous reviews[4,6], we found that some of the variation in AKI prognosis may be explained by pre-AKI CKD and post-AKI non-recovery which both lead to a poorer prognosis. This is of great relevance given that these factors were infrequently addressed, but may be a source of selection bias

For peer review only - http://bmjopen.bmj.com/site/about/guidelines.xhtml

and misclassification of CKD patients. The relationships are complex with diminishing additional risk with advancing pre-AKI CKD. We also noted a non-linear clinical post-AKI course, with risk prediction dependent on the time point at which risk is assessed, necessitating fixed follow up points to prevent overestimation of risk in a cohort with shorter follow up. Importantly, few studies assessed these factors, using different definitions, and with only one study assessing both pre-AKI and post-AKI factors.

The NICE guidelines call for further studies of long term AKI prognosis but without specific detail on which factors should be included. We suggest that future studies should adopt consistent definitions, incorporate baseline and recovery function and consider the timing of outcomes during follow up. We therefore suggest that future long term prognostic studies should adopt the KDIGO definition for identifying and stratifying AKI[2], the KDIGO CKD stages for stratifying AKI and non-AKI comparator groups by pre-AKI eGFR[42], the UK Renal Association definition of renal recovery for stratifying recovery (within 20% of baseline)[3] and fixed interim points for establishing recovery (7, 30, 90 days) and excluding early outcomes (up to 30, 90, and 180 days).

CONCLUSION

AKI has a poor, but variable prognosis and there is a recognised need to understand which patients are at greater long-term risk. Poor prognosis may in part be explained by pre-AKI CKD which is associated with doubling of mortality outcomes and a four-fivefold increase in renal outcomes. It may also be explained by non-recovery of kidney function, although the magnitude depends on both recovery definition and the timing of assessment. Both factors should be taken into account when studying AKI prognosis, and while they naturally form part of risk assessment in clinical practice, unfortunately few heterogenous prognostic studies have addressed them. We suggest that future prognostic studies consistently consider these prognostic factors in order to confirm their predictive value and enable integration into predictive models for AKI prognosis.

Contributorship

SS, AM, NF and CB contributed to the design of the review, SS and MM acquired the data. SS, AM and CB contributed to analysis. SS drafted and SS, AM, NF and CB critcally revised the intellectual content of the work. All authors have given final approval for this paper to be published.

Competing Interests

None declared.

Funding

SS is supported by a research training fellowship from the Wellcome Trust.

Acknowledgements

None declared.

Data Sharing

No additional data available.

For peer review only - http://bmjopen.bmj.com/site/about/guidelines.xhtml

REFERENCES

1. Ftouh S, Lewington A, on behalf of the Acute Kidney Injury Guideline Development Group convened by the National Clinical Guidelines Centre and commissioned by the National Institute for Health and Care Excellence,in association with The Royal College of Physicians' Clinical Effectiveness and Evaluation Unit. Prevention, detection and management of acute kidney injury: concise guideline. *Clinical Medicine* 2014, 14:61-5.
2. Kidney Disease: Improving Global Outcomes (KDIGO) Acute Kidney Injury Work Group. KDIGO Clinical Practice Guideline for Acute Kidney Injury. *Kidney Int* 2012, Suppl2:1-138.
3. Lewington A, Kanagasundaram S. Clinical Practice Guidelines. Acute Kidney Injury. 5th ed.: UK Renal Association; 2011.
4. Coca SG, Yusuf B, Shlipak MG, et al. Long-term risk of mortality and other adverse outcomes after acute kidney injury: a systematic review and meta-analysis. *Am J Kidney Dis* 2009, 53:961-973.
5. Bydash JR, Ishani A. Acute kidney injury and chronic kidney disease: a work in progress. *Clin J Am Soc Nephrol* 2011, 6:2555-2557.
6. Coca SG, Singanamala S, Parikh CR. Chronic kidney disease after acute kidney injury: a systematic review and meta-analysis. *Kidney Int* 2012, 81:442-448.
7. Chawla LS, Kimmel PL. Acute kidney injury and chronic kidney disease: an integrated clinical syndrome. *Kidney Int* 2012, 82:516-524.
8. James MT, Hemmelgarn BR, Wiebe N, et al. Glomerular filtration rate, proteinuria, and the incidence and consequences of acute kidney injury: a cohort study. *Lancet* 2010, 376:2096-2103.
9. Ishani A, Nelson D, Clothier B, et al. The magnitude of acute serum creatinine increase after cardiac surgery and the risk of chronic kidney disease, progression of kidney disease, and death. *Arch Intern Med* 2011, 171:226-233.
10. Stroup DF, Berlin JA, Morton SC, et al. Meta-analysis of observational studies in epidemiology: a proposal for reporting. Meta-analysis Of Observational Studies in Epidemiology (MOOSE) group. JAMA 2000, 283(15):2008-2012.
11. Hayden JA, Cote P, Bombardier C. Evaluation of the quality of prognosis studies in systematic reviews. *Ann Intern Med* 2006, 144:427-437.
12. Schiffl H, Fischer R. Five-year outcomes of severe acute kidney injury requiring renal replacement therapy. *Nephrol Dial Transplant* 2008, 23:2235-2241.
13. Bucaloiu ID, Kirchner HL, Norfolk ER, et al. Increased risk of death and de novo chronic kidney disease following reversible acute kidney injury. *Kidney Int* 2012, 81:477-485.
14. Hsu CY, Chertow GM, McCulloch CE, et al. Nonrecovery of kidney function and death after acute on chronic renal failure. *Clin J Am Soc Nephrol* 2009, 4:891-898.
15. Jones J, Holmen J, De Graauw J, et al. Association of complete recovery from acute kidney injury with incident CKD stage 3 and all-cause mortality. *Am J Kidney Dis* 2012, 60:402-408.
16. Maioli M, Toso A, Leoncini M, et al. Persistent renal damage after contrast-induced acute kidney injury: incidence, evolution, risk factors, and prognosis. *Circulation* 2012, 125:3099-3107.

For peer review only - http://bmjopen.bmj.com/site/about/guidelines.xhtml

17. Kheterpal S, Tremper KK, Englesbe MJ, et al. Predictors of postoperative acute renal failure after noncardiac surgery in patients with previously normal renal function. *Anesthesiology* 2007, 107:892-902.
18. Triverio PA, Martin PY, Romand J, et al. Long-term prognosis after acute kidney injury requiring renal replacement therapy. *Nephrol Dial Transplant* 2009, 24:2186-2189.
19. Ishani A, Xue JL, Himmelfarb J, et al. Acute kidney injury increases risk of ESRD among elderly. *J Am Soc Nephrol* 2009, 20:223-228
20. Lafrance JP, Miller DR. Acute kidney injury associates with increased long-term mortality. *J Am Soc Nephrol* 2010, 21:345-352.
21. Wu VC, Huang TM, Lai CF, et al. Acute-on-chronic kidney injury at hospital discharge is associated with long-term dialysis and mortality. *Kidney Int* 2011, 80:1222-1230.
22. Ponte B, Felipe C, Muriel A, et al. Long-term functional evolution after an acute kidney injury: a 10-year study: *Nephrol Dial Transplan* 2008, 23:3859-3866.
23. Brown JR, Kramer RS, Coca SG, et al. Duration of acute kidney injury impacts long-term survival after cardiac surgery. *Ann Thorac Surg* 2010, 90:1142-1148.
24. Coca SG, King JT,Jr, Rosenthal RA, et al. The duration of postoperative acute kidney injury is an additional parameter predicting long-term survival in diabetic veterans. *Kidney Int* 2010, 78:926-933.
25. Loef BG, Epema AH, Smilde TD, et al. Immediate postoperative renal function deterioration in cardiac surgical patients predicts in-hospital mortality and long-term survival. *J Am Soc Nephrol* 2005, 16:195-200.
26. Mehta RH, Honeycutt E, Patel UD, et al. Impact of recovery of renal function on long-term mortality after coronary artery bypass grafting. *Am J Cardiol* 2010, 106:1728-1734.
27. van Kuijk JP, Flu WJ, Chonchol M, et al. Temporary perioperative decline of renal function is an independent predictor for chronic kidney disease. *Clin J Am Soc Nephrol* 2010, 5:1198-1204.
28. Hoste EA, Doom S, De Waele J, et al. Epidemiology of contrast-associated acute kidney injury in ICU patients: a retrospective cohort analysis. *Intensive Care Med* 2011, 37:1921-1931.
29. Lopes JA, Fernandes P, Jorge S, et al. Long-term risk of mortality after acute kidney injury in patients with sepsis: a contemporary analysis. *BMC Nephrology* 2010, 11:9.
30. Manns B, Doig CJ, Lee H, et al. Cost of acute renal failure requiring dialysis in the intensive care unit: clinical and resource implications of renal recovery. *Crit Care Med* 2003, 31:449-455.
31. Lo LJ, Go AS, Chertow GM, et al. Dialysis-requiring acute renal failure increases the risk of progressive chronic kidney disease. *Kidney Int* 2009, 76:893-899.
32. Ng KP, Chanouzas D, Fallouh B, et al. Short and long-term outcome of patients with severe acute kidney injury requiring renal replacement therapy. *QJM* 2012, 105:33-39.
33. Wald R, Quinn RR, Adhikari NK, et al. Risk of chronic dialysis and death following acute kidney injury. *Am J Med* 2012, 125:585-593.

For peer review only - http://bmjopen.bmj.com/site/about/guidelines.xhtml

34. Gupta R, Gurm HS, Bhatt DL, et al. Renal failure after percutaneous coronary intervention is associated with high mortality. *Catheter Cardiovasc Interv* 2005, 64:442-448.
35. Kimura T, Obi Y, Yasuda K, et al. Effects of chronic kidney disease and post-angiographic acute kidney injury on long-term prognosis after coronary artery angiography. *Nephrol Dial Transplan* 2011, 26:1838-1846.
36. Lindsay J, Apple S, Pinnow EE, et al. Percutaneous coronary intervention-associated nephropathy foreshadows increased risk of late adverse events in patients with normal baseline serum creatinine. *Catheter Cardiovasc Interv* 2003, 59:338-343.
37. Rihal CS, Textor SC, Grill DE, et al. Incidence and prognostic importance of acute renal failure after percutaneous coronary intervention. *Circulation* 2002, 105:2259-2264.
38. Roghi A, Savonitto S, Cavallini C, et al. Impact of acute renal failure following percutaneous coronary intervention on long-term mortality. *J Cardiovasc Med* 2008, 9:375-381.
39. Luckraz H, Gravenor MB, George R, et al. Long and short-term outcomes in patients requiring continuous renal replacement therapy post cardiopulmonary bypass. *Eur J Cardiothorac Surg* 2005, 27:906-909.
40. Swaminathan M, Hudson CC, Phillips-Bute BG, et al. Impact of early renal recovery on survival after cardiac surgery-associated acute kidney injury. *Ann Thorac Surg* 2010, 89:1098-1104.
41. Pannu N, James M, Hemmelgarn B, et al. Association between AKI, Recovery of Renal Function, and Long-Term Outcomes after Hospital Discharge. *Clin J Am Soc Nephrol* 2013, 8:194-202.
42. Stevens PE, Levin A, Kidney Disease: Improving Global Outcomes Chronic Kidney Disease Guideline Development Work Group Members. Evaluation and management of chronic kidney disease: synopsis of the kidney disease: improving global outcomes 2012 clinical practice guideline. Ann Intern Med 2013, 158(11):825-830.

For peer review only

For peer review only - http://bmjopen.bmj.com/site/about/guidelines.xhtml

Figure 1- Quality Assessment Criteria

Figure 2- Study Selection and Quality Assessment

Figure 3 - Mortality – By Pre-AKI Baseline

w/o rec = without recovery, with rec = with recovery, HR = hazard ratio, CI = confidence interval

Figure 4 - Mortality – By Post AKI Recovery

w/o rec = without recovery, with rec = with recovery, HR = hazard ratio, CI = confidence interval

Figure 5 - CKD Outcomes

w/o rec = without recovery, with rec = with recovery, HR = hazard ratio, CI = confidence interval

For peer review only

1	Was there a clear objective description for AKI and comparators?
2	Was there a pre AKI assessment of kidney function (creatinine or CKD code) to identify AKI and baseline kidney function?
3	Did the study focus specifically on AKI survivors at hospital discharge or at least 30 days for calculating outcomes?
4	Was pre-AKI baseline CKD (eGFR <60ml/min/1.73m^2) used to stratify or analyze outcomes separately against a non-AKI comparator?
5	Was post-AKI renal recovery clearly described and used to stratify or analyses outcomes separately against a non-AKI comparator?
6	Was the timing of risk prediction and outcomes considered as a potential source of variation in findings?

Figure 1- Quality Assessment Criteria
931x267mm (96 x 96 DPI)

For peer review only

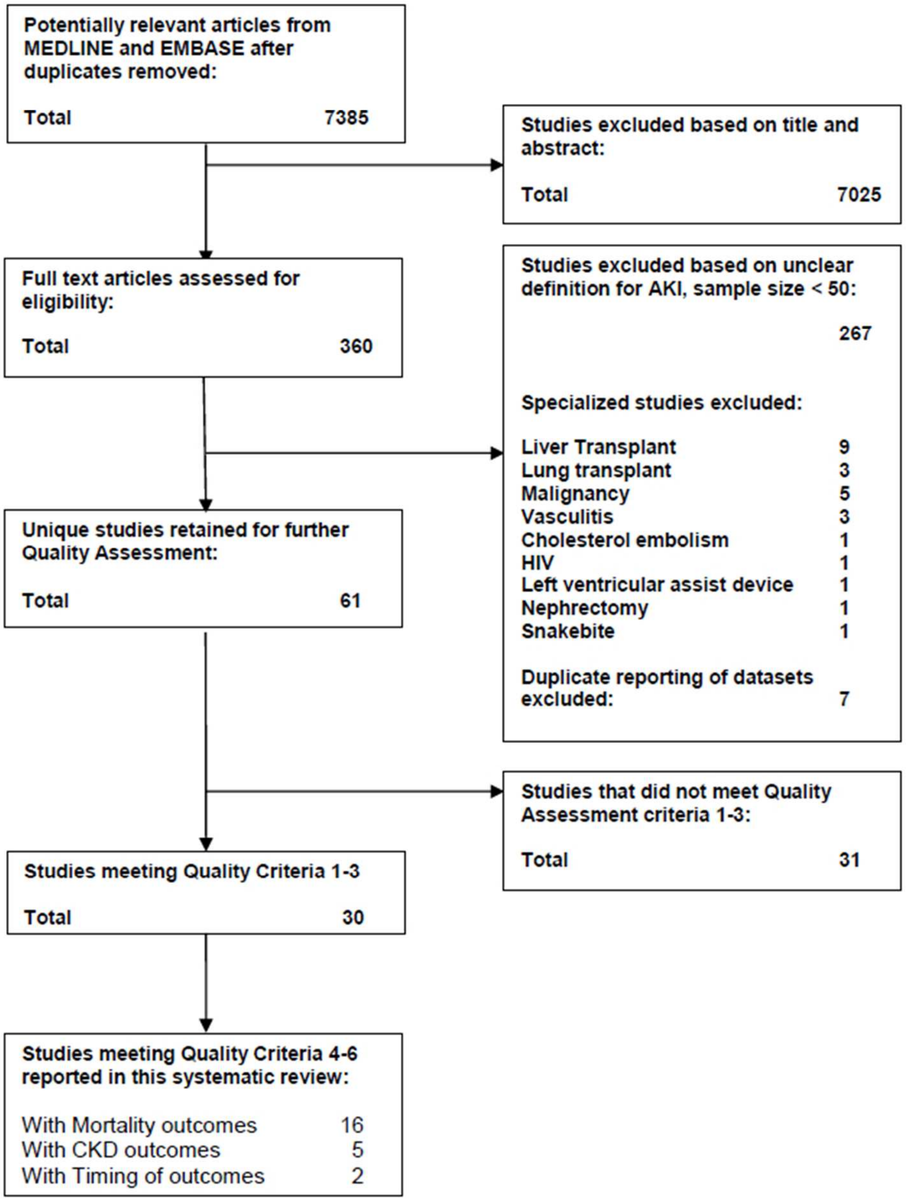

Figure 2- Study Selection and Quality Assessment
166x219mm (96 x 96 DPI)

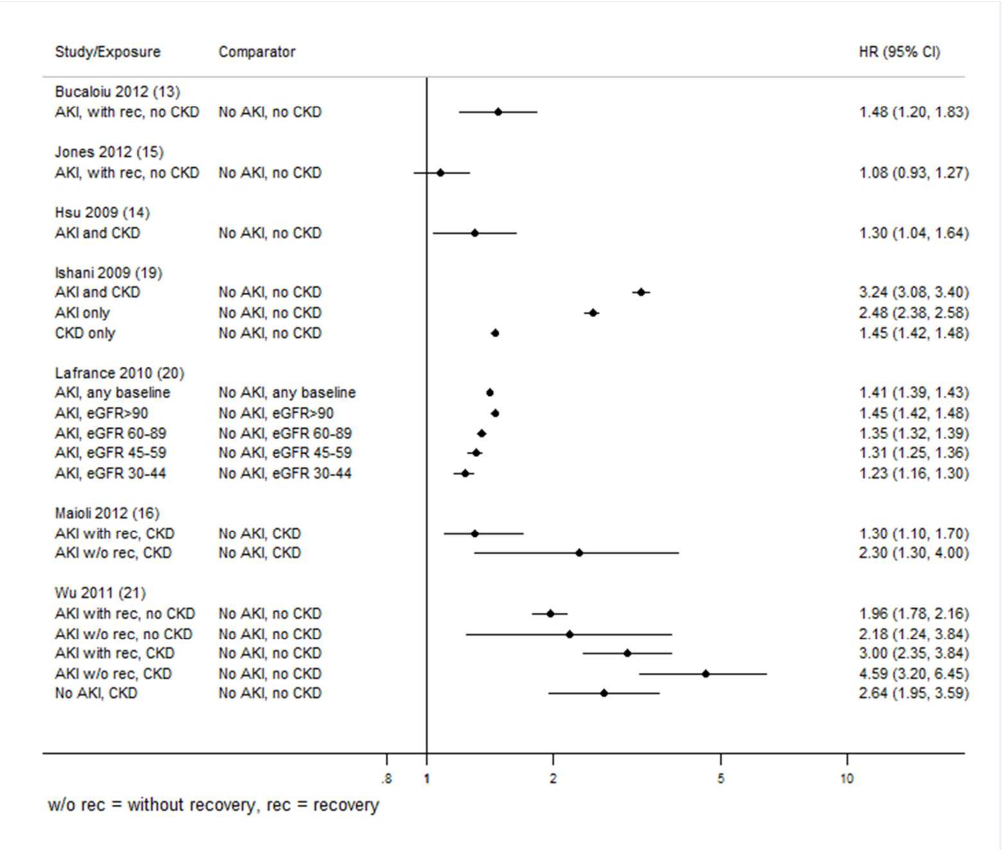

Figure 3 - Mortality – By Pre-AKI Baseline

w/o rec = without recovery, with rec = with recovery, HR = hazard ratio, CI = confidence interval
256x218mm (72 x 72 DPI)

For peer review only - http://bmjopen.bmj.com/site/about/guidelines.xhtml

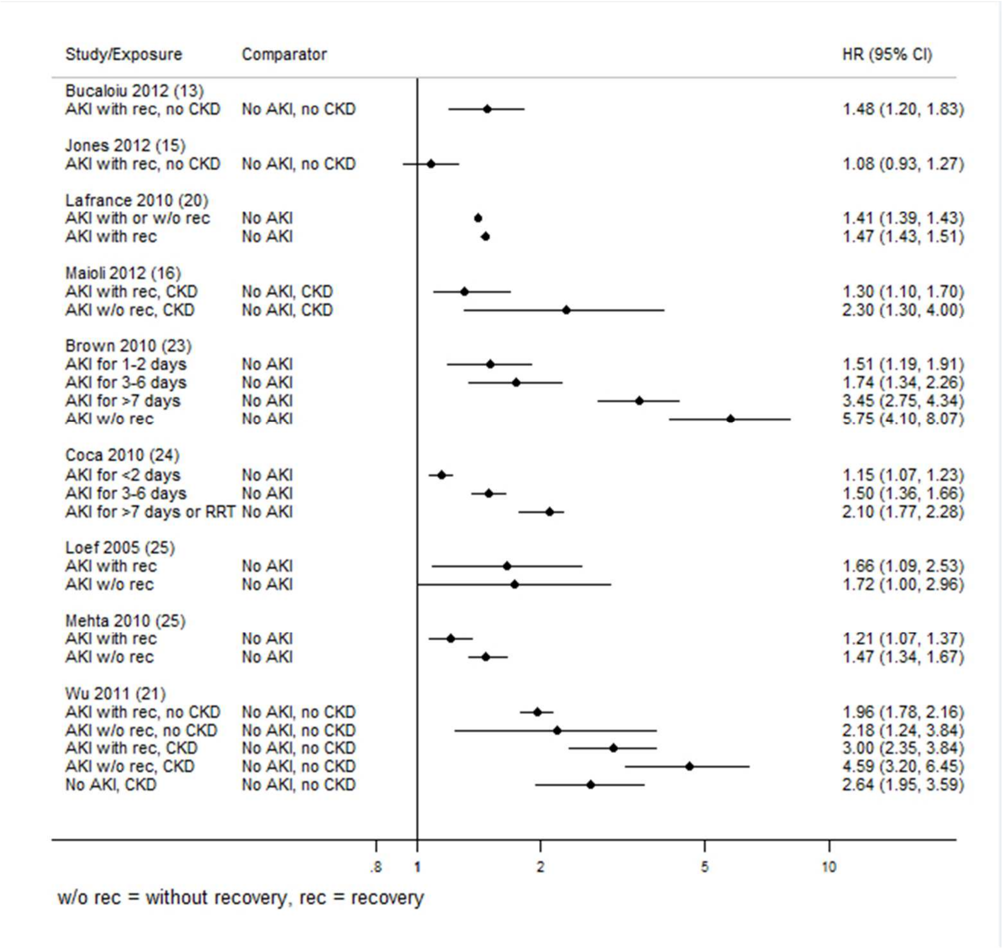

Figure 4 - Mortality – By Post AKI Recovery

w/o rec = without recovery, with rec = with recovery, HR = hazard ratio, CI = confidence interval
232x219mm (72 x 72 DPI)

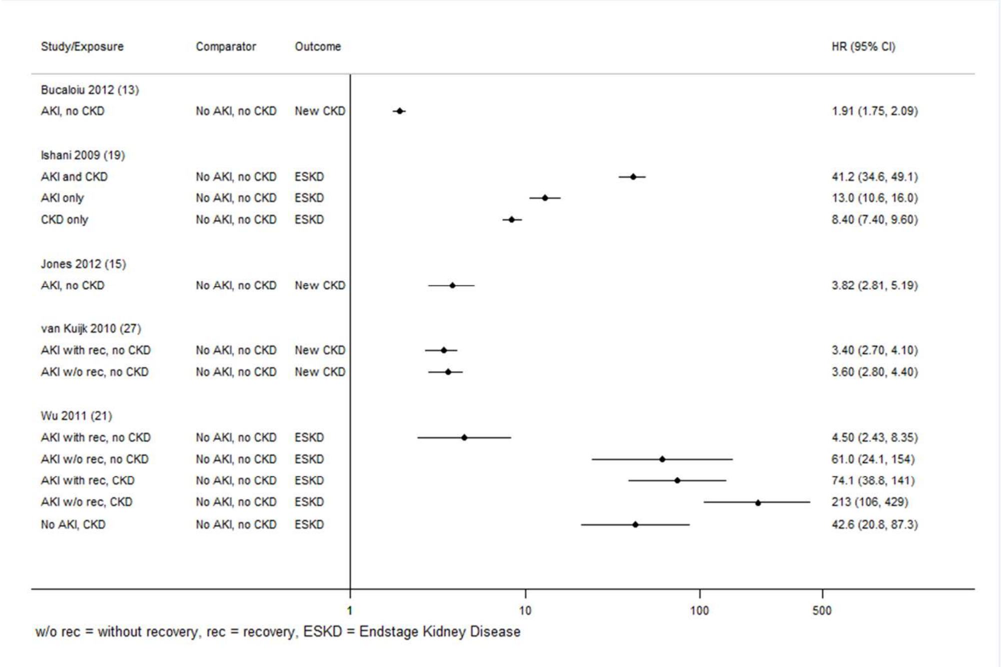

Figure 5 - CKD Outcomes

w/o rec = without recovery, with rec = with recovery, HR = hazard ratio, CI = confidence interval
327x218mm (72 x 72 DPI)

For peer review only - http://bmjopen.bmj.com/site/about/guidelines.xhtml

Supplementary material 1 – Search strategy

1. exp Renal Insufficiency, Acute/ or exp Kidney Failure, Acute/
2. acute kidney injur$.mp. [mp=title, original title, abstract, name of substance word, subject heading word, unique identifier]
3. acute kidney failure.mp.
4. acute renal failure.mp
5. (acute adj3 dialys$).mp.
6. (acute adj3 renal replac$).mp.
7. (acute adj3 renal insufficenc$).mp.
8. exp Prognosis/
9. exp regression analysis/ or exp survival analysis/
10. exp Survival/
11. exp Mortality/
12. prognos$.mp.
13. surviv$.mp.
14. mortalit$.mp.
15. follow*up.mp.
16. recover$.mp.
17. outcome$.mp.
18. course$.mp.
19. predict$.mp.
20. (chronic adj3 dialys$).mp.
21. (chronic adj3 renal replac$).mp.
22. (chronic adj3 renal insufficienc$).mp.
23. chronic kidney disease.mp.
24. chronic renal failure.mp.
25. chronic renal disease.mp.
26. exp kidney failure, chronic/ or exp renal insufficiency, chronic/
27. exp Renal Replacement Therapy/
28. end*stage kidney.mp.
29. end*stage renal.mp.
30. end stage kidney.mp.
31. end stage renal.mp.
32. follow up.mp.
33. 8 or 9 or 10 or 11 or 12 or 13 or 14 or 15 or 16 or 17 or 18 or 19 or 20 or 21 or 22 or 23 or 24 or 25 or 26 or 27 or 28 or 29 or 30 or 31 or 32
34. 1 or 2 or 3 or 4 or 5 or 6 or 7
35. 33 and 34
36. exp adolescent/ or exp child/ or exp infant/
37. child$.mp.
38. infant.mp.
39. paediatric.mp
40. pediatric.mp.
41. case report.mp.
42. exp case reports/
43. 36 or 37 or 38 or 39 or 40 or 41 or 42
44. 35 not 43
45. limit 44 to (humans and yr="1990 -Current")
46. exp epidemiologic study characteristics as topic/
47. exp clinical conference/ or exp clinical trial/ or exp comparative study/ or exp evaluation studies/ or exp meta-analysis/ or exp multicenter study/ or exp validation studies/
48. 46 or 47
49. 45 and 48

Supplementary material 2 - Quality Assessment of Included Studies

(ITU=intensive therapy unit; RRT=renal replacement therapy; N/A=Not Applicable; Cr=serum creatinine)

Study	Clinical Context	AKI Survivors	AKI Definition	Pre-AKI baseline available	Short and Long Term Outcomes Separated	Follow Up (*mean/median available)	Quality criteria 1-3 met (clear AKI definition with pre-AKI data or hospital coding, survivor definition)
Akposso 2000 [1]	ITU	229	Cr rise	N	Y	Up to 142 months	
Bagshaw 2006 [2]	ITU	97	RRT	N	Y	Up to 1 year	
Bell 2005 [3]	ITU	112	RRT	N	Y	Up to 5 years	
Bell 2007 [4]	ITU	N/A	RRT	N	N	Up to 7 years	
Bell 2009 [5]	ITU	N/A	Cr rise	N	N	Up to 1000 days	
Carl 2010 [6]	ITU	62	RRT	N	Y	Up to 1 year	
Cartin-Ceba 2009 [7]	ITU	784	Cr rise	N	Y	Up to 6 years	
Goldberg 2009 [8]	ITU	294	Cr rise	N	Y	*36 months	
Hoste 2011 [9]	ITU	82	Cr rise	Y	Y	1 year	Y
Lins 2006 [10]	ITU	145	Cr rise	N	Y	Up 1 year	
Lopes 2010 [11]	ITU	61	Cr rise	Y	Y	*21 months	Y
Manns 2003 [12]	ITU	66	RRT	Y	Y	Up to 1 year	Y
Morgera 2002 [13]	ITU	267	RRT	N	Y	*938 days	
Schiffl 2006,2008, 2012, 2012 [14-17]	ITU	226	RRT	Y	Y	Up to 1-10 years	Y
Triverio 2009 [18]	ITU	95	RRT	Y	Y	Up to 3 years	Y

For peer review only

Ahlstrom 2005 [19]	Unselected	N/A	RRT	N	N	*3.9 years	
Amdur 2009, Chawla 2011 [20,21]	Unselected	5366	Code	Y	N	Up to 60 months	
Bhandari 1996 [22]	Unselected	107	RRT	N	Y	Up to 5 years	
Bucaloiu 2012 [23]	Unselected	1610	Cr rise	Y	Y	*3.3 years	Y
Frost 1993 [24]	Unselected	N/A	RRT	N	N	5 years	
Hsu 2009 [25]	Unselected	782	RRT	Y	Y	4 years	Y
Ishani 2009 [26]	Unselected	7197	Code	Code	Y	2 years	Y
James 2010B [27]	Unselected	N/A	Code	Y	N	*35 months	
Jones 2012 [28]	Unselected	719	Code	Y	Y	*2.5 years	Y
Lafrance 2010A [29]	Unselected	82711	Cr rise	Y	Y	*2.34 years	Y
Lafrance 2010B [30]	Unselected	N/A	Cr rise	Y	N	*19.4 months	
Lee 2011 [31]	Unselected	131	RRT	Y	Y	5 years	
Liano 2007, Ponte 2008 [32,33]	Unselected	177	Cr rise	Y	Y	*7.2 years	Y
Lo 2009 [34]	Unselected	343	RRT	Y	Y	10344 person-yrs	Y
Murugan 2010 [35]	Unselected	561	Cr rise	Estimated baselines	Y	1 year	
Ng 2011 [36]	Unselected	262	RRT	Y	Y	1 year	Y
Thakar 2011 [37]	Unselected	N/A	Cr rise	N	N	*61.2 months	
Wald 2009 [38]	Unselected	3769	RRT	N	Y	*3 years	
Wald 2012 [39]	Unselected	41327	Code	Code	Y	*2 years	Y
Amin 2010 [40]	Cardiac	393	Cr rise	N	Y	4 year	
Gupta 2005 [41]	Cardiac	143	Cr rise	Y	Y	*38 months	Y
Hwang 2011 [42]	Cardiac	N/A	Cr rise	N	N	1 year	
James 2010A, James 2011 [43,44]	Cardiac	553	Cr rise	Y	N	*19.7 months	
Kim 2011 [45]	Cardiac	74	Cr rise	N	Y	*358 days	
Kimura 2010 [46]	Cardiac	81	Cr rise	Y	Y	*3.8 years	Y
Lindsay 2003 [47]	Cardiac	179	Cr rise	Y	Y	1 year	Y
Maioli 2012 [48]	Cardiac	167	Cr rise	Y	Y	*3.8 years	Y
Newsome 2008 [49]	Cardiac	18069	Cr rise	N	Y	*4.1 years	
Rihal 2002 [50]	Cardiac	185	Cr rise	Y	Y	5 years	Y
Roghi 2008 [51]	Cardiac	106	Cr rise	Y	Y	2 years	Y
Bihorac 2009 [52]	Postoperative	3326	Cr rise	Estimated baselines	Y	>10 years	
Brown 2010 [53]	Postoperative	N/A	Cr rise	Y	Y	*2.6 years	Y
Coca 2010 [54,55]	Postoperative	6257	Cr rise	Y	Y	*3.8 years	Y
Hein 2006 [56]	Postoperative	148	RRT	N	Y	3 years	
Hobson 2009 [57]	Postoperative	861	Cr rise	Estimated baselines	Y	10 years	
Ishani 2011 [58]	Postoperative	4053	Cr rise	Y	Y	*5.1 years	Y
Jafari 2010 [59]	Postoperative	94	Cr rise	N	Y	1 year	
Kheterpal 2007[60]	Postoperative	101	Cr rise	Y	Y	1 year	Y
Loef 2005 [61]	Postoperative	145	Cr rise	Y	Y	100 months	Y
Luckraz 2005 [62]	Postoperative	53	RRT	Y	Y	5 years	Y
Mehta 2010 [63]	Postoperative	2083	Cr rise	Y	Y	*7 years	Y
Swaminathan 2010 [64]	Postoperative	1113	Cr rise	Y	Y	135 months	Y
van Kuijk 2010 [65]	Postoperative	493	Cr rise	Y	Y	*5 years	Y
van Kuijk 2011 [66]	Postoperative	N/A	Cr rise	Y	N	*2.2 years	
Welten 2007 [67]	Postoperative	384	Cr rise	Y	N	*6 years	
Wu 2011 [68]	Postoperative	4393	Cr rise	Y	Y	*4.76 years	Y

1. Akposso K, Hertig A, Couprie R, Flahaut A, Alberti C, Karras GA, Haymann JP, Costa De Beauregard MA, Lahlou A, Rondeau E, Sraer JD: Acute renal failure in patients over 80 years old: 25-years' experience. *Intensive Care Med* 2000, 26:400-406.
2. Bagshaw SM, Mortis G, Doig CJ, Godinez-Luna T, Fick GH, Laupland KB: One-year mortality in critically ill patients by severity of kidney dysfunction: a population-based assessment. *Am J Kidney Dis* 2006, 48:402-409.
3. Bell M, Liljestam E, Granath F, Fryckstedt J, Ekbom A, Martling CR: Optimal follow-up time after continuous renal replacement therapy in actual renal failure patients stratified with the RIFLE criteria. *Nephrol Dial Transplant* 2005, 20:354-360.
4. Bell M, Swing, Granath F, Schon S, Ekbom A, Martling CR: Continuous renal replacement therapy is associated with less chronic renal failure than intermittent haemodialysis after acute renal failure. *Intensive Care Med* 2007, 33:773-780.
5. Bell M. Granath F. Martensson J. Lofberg E. Ekbom A. Martling CR. KING (Karolinska Intensive care Nephrology Group): Cystatin C is correlated with mortality in patients with and without acute kidney injury. *Nephrol Dial Transplant* 2009, 24:3096-3102.

For peer review only

6. Carl DE, Grossman C, Behnke M, Sessler CN, Gehr TW. Effect of timing of dialysis on mortality in critically ill, septic patients with acute renal failure. *Hemodial Int* 2010, 14:11-17.
7. Cartin-Ceba R, Haugen EN, Iscimen R, Trillo-Alvarez C, Juncos L, Gajic O: Evaluation of "Loss" and "End stage renal disease" after acute kidney injury defined by the Risk, Injury, Failure, Loss and ESRD classification in critically ill patients. *Intensive Care Med* 2000, 35:2087-2095.
8. Goldberg A, Kogan E, Hammerman H, Markiewicz W, Aronson D: The impact of transient and persistent acute kidney injury on long-term outcomes after acute myocardial infarction. *Kidney Int* 2009, 76:900-906.
9. Hoste EA, Doom S, De Waele J, Delrue LJ, Defreyne L, Benoit DD, Decruyenaere J: Epidemiology of contrast-associated acute kidney injury in ICU patients: a retrospective cohort analysis. *Intensive Care Med* 2011, 37:1921-1931.
10. Lins RL, Elseviers MM, Daelemans R: Severity scoring and mortality 1 year after acute renal failure. *Nephrol Dial Transplant* 2006, 21:1066-1068.
11. Lopes JA, Fernandes P, Jorge S, Resina C, Santos C, Pereira A, Neves J, Antunes F, Gomes da Costa A: Long-term risk of mortality after acute kidney injury in patients with sepsis: a contemporary analysis. *BMC Nephrology* 2010, 11:9.
12. Manns B, Doig CJ, Lee H, Dean S, Tonelli M, Johnson D, Donaldson C: Cost of acute renal failure requiring dialysis in the intensive care unit: clinical and resource implications of renal recovery. *Crit Care Med* 2003, 31:449-455.
13. Morgera S, Kraft AK, Siebert G, Luft FC, Neumayer HH: Long-term outcomes in acute renal failure patients treated with continuous renal replacement therapies. *Am J Kidney Dis* 2002, 40:275-279.
14. Schiffl H: Renal recovery from acute tubular necrosis requiring renal replacement therapy: a prospective study in critically ill patients. *Nephrol Dial Transplant* 2006, 21:1248-1252.
15. Schiffl H, Fischer R: Five-year outcomes of severe acute kidney injury requiring renal replacement therapy. *Nephrol Dial Transplant* 2008, 23:2235-2241.
16. Schiffl H, Fischer R: Clinical cause of presumed acute tubular necrosis requiring renal replacement therapy and outcome of critically ill patients: post hoc analysis of a prospective 7-year cohort study. *Int Urol Nephrol 2012*, 44:1779-1789.
17. Schiffl H, Lang SM, Fischer R: Long-term outcomes of survivors of ICU acute kidney injury requiring renal replacement therapy: a 10-year prospective cohort study. *Clin Kidney J* 2012, 5:297-302.
18. Triverio PA, Martin PY, Romand J, Pugin J, Perneger T, Saudan P: Long-term prognosis after acute kidney injury requiring renal replacement therapy. *Nephrol Dial Transplant*, 24:2186-2189.
19. Ahlstrom A, Tallgren M, Peltonen S, Rasanen P, Pettila V: Survival and quality of life of patients requiring acute renal replacement therapy. *Intensive Care Med* 2005, 31:1222-1228.
20. Amdur RL, Chawla LS, Amodeo S, Kimmel PL, Palant CE: Outcomes following diagnosis of acute renal failure in U.S. veterans: focus on acute tubular necrosis. *Kidney Int* 2009, 76:1089-1097.
21. Chawla LS, Amdur RL, Amodeo S, Kimmel PL, Palant CE The severity of acute kidney injury predicts progression to chronic kidney disease. *Kidney Int* 2011, 79:1361-1369.
22. Bhandari S, Turney JH: Survivors of acute renal failure who do not recover renal function. *QJM* 1996, 89:415-421.

For peer review only

23. Bucaloiu ID, Kirchner HL, Norfolk ER, Hartle JE,2nd, Perkins RM: Increased risk of death and de novo chronic kidney disease following reversible acute kidney injury. *Kidney Int* 2012, 81:477-485.
24. Frost L, Pedersen RS, Bentzen S, Bille H, Hansen HE: Short and long term outcome in a consecutive series of 419 patients with acute dialysis-requiring renal failure. *Scand J Urol* 199, ;27:453-462.
25. Hsu CY, Chertow GM, McCulloch CE, Fan D, Ordonez JD, Go AS: Nonrecovery of kidney function and death after acute on chronic renal failure. *Clin J Am Soc Nephrol* 2009, 4:891-898.
26. Ishani A, Xue JL, Himmelfarb J, Eggers PW, Kimmel PL, Molitoris BA, Collins AJ: Acute kidney injury increases risk of ESRD among elderly. *J Am Soc Nephrol* 2009, 20:223-228.
27. James MT, Hemmelgarn BR, Wiebe N, Pannu N, Manns BJ, Klarenbach SW, Tonelli M, Alberta Kidney Disease N: Glomerular filtration rate, proteinuria, and the incidence and consequences of acute kidney injury: a cohort study. *Lancet* 2010, 376:2096-2103.
28. Jones J, Holmen J, De Graauw J, Jovanovich A, Thornton S, Chonchol M: Association of complete recovery from acute kidney injury with incident CKD stage 3 and all-cause mortality. *Am J Kidney Dis* 2012, 60:402-408.
29. Lafrance JP, Miller D:. Acute kidney injury associates with increased long-term mortality. *J Am Soc Nephrol* 2010, 21:345-352.
30. Lafrance JP, Djurdjev O, Levin A: Incidence and outcomes of acute kidney injury in a referred chronic kidney disease cohort. *Nephrol Dial Transplant* 2010, 25:2203-2209.
31. Lee PH, Wu VC, Hu FC, Lai CF, Chen YM, Tsai TJ, Wu KD, National Taiwan University Study Group on Acute Renal Failure: Outcomes following dialysis for acute kidney injury among different stages of chronic kidney disease. *Am J Nephrol* 2011, 34:95-103.
32. Liano F, Felipe C, Tenorio MT, Rivera M, Abraira V, Saez-de-Urturi JM, Ocana J, Fuentes C, Severiano S: Long-term outcome of acute tubular necrosis: a contribution to its natural history. *Kidney Int* 2007, 71:679-686.
33. Ponte B, Felipe C, Muriel A, Tenorio MT, Liano F: Long-term functional evolution after an acute kidney injury: a 10-year study. *Nephrol Dial Transplant* 2008, 23:3859-3866.
34. Lo LJ, Go AS, Chertow GM, McCulloch CE, Fan D, Ordonez JD, Hsu CY: Dialysis-requiring acute renal failure increases the risk of progressive chronic kidney disease. *Kidney Int* 2009, 76:893-899.
35. Murugan R, Kellum JA: Acute kidney injury: what's the prognosis? *Nat Rev Nephrol* 2011, 7:209-217.
36. Ng KP, Chanouzas D, Fallouh B, Baharani J: Short and long-term outcome of patients with severe acute kidney injury requiring renal replacement therapy. *QJM* 2012, 105:33-39.
37. Thakar CV, Christianson A, Himmelfarb J, Leonard AC: Acute kidney injury episodes and chronic kidney disease risk in diabetes mellitus. *Clin J Am Soc Nephrol* 2011, 6:2567-2572.
38. Wald R, Quinn RR, Luo J, Li P, Scales DC, Mamdani MM, Ray JG, University of Toronto Acute Kidney Injury Research,Group: Chronic dialysis and death among survivors of acute kidney injury requiring dialysis. *JAMA* 2009, 302:1179-1185.
39. Wald R, Quinn RR, Adhikari NK, Burns KE, Friedrich JO, Garg AX, Harel Z, Hladunewich MA, Luo J, Mamdani M, Perl J, Ray JG, University of Toronto Acute Kidney Injury Research,Group: Risk of chronic dialysis and death following acute kidney injury. *Am J Med* 2012, 125:585-593.

For peer review only

40. Amin AP, Spertus JA, Reid KJ, Lan X, Buchanan DM, Decker C, Masoudi FA: The prognostic importance of worsening renal function during an acute myocardial infarction on long-term mortality. *Am Heart J* 2010, 160:1065-1071.
41. Gupta R, Gurm HS, Bhatt DL, Chew DP, Ellis SG: Renal failure after percutaneous coronary intervention is associated with high mortality. *Catheter Cardiovasc Interv* 2005, 64:442-448.
42. Hwang SH, Jeong MH, Ahmed K, Kim MC, Cho KH, Lee MG, Ko JS, Park KH, Sim DS, Yoon NS, Yoon HJ, Kim KH, Hong YJ, Park HW, Kim JH, Ahn YK, Cho JG, Park JC, Kang JC: Different clinical outcomes of acute kidney injury according to acute kidney injury network criteria in patients between ST elevation and non-ST elevation myocardial infarction. *Int J Cardiol* 2011, 150:99-101.
43. James MT, Ghali WA, Tonelli M, Faris P, Knudtson ML, Pannu N, Klarenbach SW, Manns BJ, Hemmelgarn BR: Acute kidney injury following coronary angiography is associated with a long-term decline in kidney function. *Kidney Int* 2010, 78:803-809.
44. James MT. Ghali WA. Knudtson ML. Ravani P. Tonelli M. Faris P. Pannu N. Manns BJ. Klarenbach SW. Hemmelgarn BR. Alberta Provincial Project for Outcome Assessment in Coronary Heart Disease (APPROACH) Investigators: Associations between acute kidney injury and cardiovascular and renal outcomes after coronary angiography. *Circulation* 2011, 123:409-416.
45. Kim MJ, Choi HS, Oh SH, Lee HC, Kim CS, Choi JS, Klarenbach SW, Manns BJ, Hemmelgarn BR: Acute kidney injury following coronary angiography is associated with a long-term decline in kidney function. *Yonsei Med J* 2011, 52:603-609.
46. Kimura T, Obi Y, Yasuda K, Sasaki K, Takeda Y, Nagai Y, Imai E, Rakugi H, Isaka Y, Hayashi T: Effects of chronic kidney disease and post-angiographic acute kidney injury on long-term prognosis after coronary artery angiography. *Nephrol Dial Transplan* 2011, 26:1838-1846.
47. Lindsay J, Apple S, Pinnow EE, Gevorkian N, Gruberg L, Satler LF, Pichard AD, Kent KM, Suddath W, Waksman R: Percutaneous coronary intervention-associated nephropathy foreshadows increased risk of late adverse events in patients with normal baseline serum creatinine. *Catheter Cardiovasc Interv* 2003, 59:338-343.
48. Maioli M, Toso A, Leoncini M, Gallopin M, Musilli N, Bellandi F: Persistent renal damage after contrast-induced acute kidney injury: incidence, evolution, risk factors, and prognosis. *Circulation* 2012, 125:3099-3107.
49. Newsome BB, Warnock DG, McClellan WM, Herzog CA, Kiefe CI, Eggers PW, Allison JJ: Long-term risk of mortality and end-stage renal disease among the elderly after small increases in serum creatinine level during hospitalization for acute myocardial infarction. *Arch Intern Med* 2008, 168:609-616.
50. Rihal CS, Textor SC, Grill DE, Berger PB, Ting HH, Best PJ, Singh M, Bell MR, Barsness GW, Mathew V, Garratt KN, Holmes DR,Jr: Incidence and prognostic importance of acute renal failure after percutaneous coronary intervention. *Circulation* 2002, 105:2259-2264.
51. Roghi A. Savonitto S. Cavallini C. Arraiz G. Angoli L. Castriota F. Bernardi G. Sansa M. De Servi S. Pitscheider W. Danzi GB. Reimers B. Klugmann S. Zaninotto M. Ardissino D. Atherosclerosis, Thrombosis and Vascular Biology Study Group and the Italian Society for Invasive Cardiology Investigators: Impact of acute renal failure following percutaneous coronary intervention on long-term mortality. *J Cardiovasc Med* 2008, 9:375-381.
52. Bihorac A, Yavas S, Subbiah S, Hobson CE, Schold JD, Gabrielli A, Layon AJ, Segal MS: Long-term risk of mortality and acute kidney injury during hospitalization after major surgery. *Ann Surg* 2009, 249:851-858.

For peer review only

53. Brown JR, Kramer RS, Coca SG, Parikh CR: Duration of acute kidney injury impacts long-term survival after cardiac surgery. *Ann Thorac Surg* 2010, 90:1142-1148.
54. Coca SG, King JT,Jr, Rosenthal RA, Perkal MF, Parikh CR: The duration of postoperative acute kidney injury is an additional parameter predicting long-term survival in diabetic veterans. *Kidney Int* 2010, 78:926-933.
55. Coca SG: Long-term outcomes of acute kidney injury. *Curr Opin Nephrol Hypertens* 2010, 19:266-272.
56. Hein OV, Birnbaum J, Wernecke KD, Konertz W, Jain U, Spies C: Three-year survival after four major post-cardiac operative complications. *Crit Care Med* 2006, 34:2729-2737.
57. Hobson CE, Yavas S, Segal MS, Schold JD, Tribble CG, Layon AJ, Bihorac A: Acute kidney injury is associated with increased long-term mortality after cardiothoracic surgery. *Circulation* 2009, 119:2444-2453.
58. Ishani A, Nelson D, Clothier B, Schult T, Nugent S, Greer N, Slinin Y, Ensrud KE: The magnitude of acute serum creatinine increase after cardiac surgery and the risk of chronic kidney disease, progression of kidney disease, and death. *Arch Intern Med* 2011, 171:226-233.
59. Jafari SM, Huang R, Joshi A, Parvizi J, Hozack WJ: Renal impairment following total joint arthroplasty: who is at risk? *J Arthroplasty* 2010, 25:49; Se-53.
60. Kheterpal S, Tremper KK, Englesbe MJ, O'Reilly M, Shanks AM, Fetterman DM, Rosenberg AL, Swartz RD: Predictors of postoperative acute renal failure after noncardiac surgery in patients with previously normal renal function. *Anesthesiology* 2007, 107:892-902.
61. Loef BG, Epema AH, Smilde TD, Henning RH, Ebels T, Navis G, Stegeman CA: Immediate postoperative renal function deterioration in cardiac surgical patients predicts in-hospital mortality and long-term survival. *J Am Soc Nephrol* 2005, 16:195-200.
62. Luckraz H, Gravenor MB, George R, Taylor S, Williams A, Ashraf S, Argano V, Youhana A: Long and short-term outcomes in patients requiring continuous renal replacement therapy post cardiopulmonary bypass. *Eur J Cardiothorac Surg* 2005, 27:906-909.
63. Mehta RH, Honeycutt E, Patel UD, Lopes RD, Shaw LK, Glower DD, Harrington RA, Califf RM, Sketch MH,Jr: Impact of recovery of renal function on long-term mortality after coronary artery bypass grafting. *Am J Cardiol* 2010, 106:1728-1734.
64. Swaminathan M, Hudson CC, Phillips-Bute BG, Patel UD, Mathew JP, Newman MF, Milano CA, Shaw AD, Stafford-Smith M: Impact of early renal recovery on survival after cardiac surgery-associated acute kidney injury. *Ann Thorac Surg* 2010, 89:1098-1104.
65. van Kuijk JP, Flu WJ, Chonchol M, Hoeks SE, Winkel TA, Verhagen HJ, Bax JJ, Poldermans D: Temporary perioperative decline of renal function is an independent predictor for chronic kidney disease. *Clin J Am Soc Nephrol* 2010, 5:1198-1204.
66. van Kuijk JP, Flu WJ, Valentijn TM, Chonchol M, Voute MT, Kuiper RJ, Verhagen HJ, Bax JJ, Poldermans D: Preoperative left ventricular dysfunction predisposes to postoperative acute kidney injury and long-term mortality. *J Nephrol* 2011, 24:764-770.
67. Welten GM, Schouten O, Chonchol M, Hoeks SE, Feringa HH, Bax JJ, Dunkelgrun M, van Gestel YR, van Domburg RT, Poldermans D: Temporary worsening of renal function after aortic surgery is associated with higher long-term mortality. *Am J Kidney Dis* 2007, 50:219-228.
68. Wu VC, Huang TM, Lai CF, Shiao CC, Lin YF, Chu TS, Wu PC, Chao CT, Wang JY, Kao TW, Young GH, Tsai PR, Tsai HB, Wang CL, Wu MS, Chiang WC, Tsai IJ, Hu FC, Lin SL, Chen YM, Tsai TJ, Ko WJ, Wu KD: Acute-on-chronic kidney injury at

For peer review only

hospital discharge is associated with long-term dialysis and mortality. *Kidney Int* 2011, 80:1222-1230.

For peer review only

PRISMA 2009 Checklist

Section/topic	#	Checklist item	Reported on page #
**TITLE**			
Title	1	Identify the report as a systematic review, meta-analysis, or both.	p1
**ABSTRACT**			
Structured summary	2	Provide a structured summary including, as applicable: background; objectives; data sources; study eligibility criteria, participants, and interventions; study appraisal and synthesis methods; results; limitations; conclusions and implications of key findings; systematic review registration number.	p2
**INTRODUCTION**			
Rationale	3	Describe the rationale for the review in the context of what is already known.	p4
Objectives	4	Provide an explicit statement of questions being addressed with reference to participants, interventions, comparisons, outcomes, and study design (PICOS).	p4
**METHODS**			
Protocol and registration	5	Indicate if a review protocol exists, if and where it can be accessed (e.g., Web address), and, if available, provide registration information including registration number.	n/a
Eligibility criteria	6	Specify study characteristics (e.g., PICOS, length of follow-up) and report characteristics (e.g., years considered, language, publication status) used as criteria for eligibility, giving rationale.	pp4-5
Information sources	7	Describe all information sources (e.g., databases with dates of coverage, contact with study authors to identify additional studies) in the search and date last searched.	pp4-5
Search	8	Present full electronic search strategy for at least one database, including any limits used, such that it could be repeated.	Supplementary material
Study selection	9	State the process for selecting studies (i.e., screening, eligibility, included in systematic review, and, if applicable, included in the meta-analysis).	pp4-5, Figure 2
Data collection process	10	Describe method of data extraction from reports (e.g., piloted forms, independently, in duplicate) and any processes for obtaining and confirming data from investigators.	p5
Data items	11	List and define all variables for which data were sought (e.g., PICOS, funding sources) and any assumptions and simplifications made.	p5
Risk of bias in individual studies	12	Describe methods used for assessing risk of bias of individual studies (including specification of whether this was done at the study or outcome level), and how this information is to be used in any data synthesis.	p5, Figure 1
Summary measures	13	State the principal summary measures (e.g., risk ratio, difference in means).	pp5-6
Synthesis of results	14	Describe the methods of handling data and combining results of studies, if done, including measures of consistency (e.g., I^2) for each meta-analysis.	pp5-6

For peer review only

PRISMA 2009 Checklist

Section/topic	#	Checklist item	Reported on page #
Risk of bias across studies	15	Specify any assessment of risk of bias that may affect the cumulative evidence (e.g., publication bias, selective reporting within studies).	n/a
Additional analyses	16	Describe methods of additional analyses (e.g., sensitivity or subgroup analyses, meta-regression), if done, indicating which were pre-specified.	n/a
**RESULTS**			
Study selection	17	Give numbers of studies screened, assessed for eligibility, and included in the review, with reasons for exclusions at each stage, ideally with a flow diagram.	p6
Study characteristics	18	For each study, present characteristics for which data were extracted (e.g., study size, PICOS, follow-up period) and provide the citations.	p6
Risk of bias within studies	19	Present data on risk of bias of each study and, if available, any outcome level assessment (see item 12).	p6, Table 1
Results of individual studies	20	For all outcomes considered (benefits or harms), present, for each study: (a) simple summary data for each intervention group (b) effect estimates and confidence intervals, ideally with a forest plot.	p6-7<br>Tables 2,3<br>Figures 3-5
Synthesis of results	21	Present results of each meta-analysis done, including confidence intervals and measures of consistency.	n/a
Risk of bias across studies	22	Present results of any assessment of risk of bias across studies (see Item 15).	n/a
Additional analysis	23	Give results of additional analyses, if done (e.g., sensitivity or subgroup analyses, meta-regression [see Item 16]).	n/a
**DISCUSSION**			
Summary of evidence	24	Summarize the main findings including the strength of evidence for each main outcome; consider their relevance to key groups (e.g., healthcare providers, users, and policy makers).	pp7-8
Limitations	25	Discuss limitations at study and outcome level (e.g., risk of bias), and at review-level (e.g., incomplete retrieval of identified research, reporting bias).	pp8
Conclusions	26	Provide a general interpretation of the results in the context of other evidence, and implications for future research.	p9
**FUNDING**			
Funding	27	Describe sources of funding for the systematic review and other support (e.g., supply of data); role of funders for the systematic review.	p9

From: Moher D, Liberati A, Tetzlaff J, Altman DG, The PRISMA Group (2009). Preferred Reporting Items for Systematic Reviews and Meta-Analyses: The PRISMA Statement. PLoS Med 6(6): e1000097. doi:10.1371/journal.pmed1000097

For more information, visit: www.prisma-statement.org

For peer review only

For peer review only - http://bmjopen.bmj.com/site/about/guidelines.xhtml

PRISMA 2009 Checklist

For peer review only

BMJ Open

Long Term Prognosis after Acute Kidney Injury (AKI) - What is the Role of Baseline Kidney Function and Recovery? A Systematic Review

Journal:	*BMJ Open*
Manuscript ID:	bmjopen-2014-006497.R1
Article Type:	Research
Date Submitted by the Author:	12-Nov-2014
Complete List of Authors:	Sawhney, Simon; University of Aberdeen, Division of Applied Health Sciences; Aberdeen Royal Infirmary, Renal Unit, Ward 108<br>Mitchell, Mhairi; University of Aberdeen, Division of Applied Health Sciences<br>Fluck, Nick; Aberdeen Royal Infirmary, Renal Unit, Ward 108<br>Marks, Angharad; University of Aberdeen, Division of Applied Health Sciences; Aberdeen Royal Infirmary, Renal Unit, Ward 108<br>Black, Corrinda; University of Aberdeen, Division of Applied Health Sciences
**Primary Subject Heading**:	Epidemiology
Secondary Subject Heading:	Renal medicine
Keywords:	EPIDEMIOLOGY, Acute renal failure < NEPHROLOGY, Chronic renal failure < NEPHROLOGY, PUBLIC HEALTH

SCHOLARONE™
Manuscripts

For peer review only

Long Term Prognosis after Acute Kidney Injury (AKI) - What is the Role of Baseline Kidney Function and Recovery? A Systematic Review

Simon Sawhney[1,2*] MBChB
simon.sawhney@abdn.ac.uk

Mhairi Mitchell[1] MBChB
m.mitchell.08@aberdeen.ac.uk

Angharad Marks[1,2] MBBCh
a.marks@abdn.ac.uk

Nick Fluck[2] MBBS
nfluck@nhs.net

Corrinda Black[1] MBChB
corri.black@abdn.ac.uk

[1]Division of Applied Health Sciences, University of Aberdeen
[2]Renal Unit,Ward 108 Aberdeen Royal Infirmary

[*]Correspondence to:
Simon Sawhney, Division of Applied Health Sciences, University of Aberdeen, Polwarth Building, Foresterhill, Aberdeen AB25 2ZD, UK

Key words:
Acute Kidney Injury
Chronic Renal Insufficiency
Epidemiology
Systematic Review

Word count: 2705 words

For peer review only

For peer review only - http://bmjopen.bmj.com/site/about/guidelines.xhtml

ABSTRACT

Objectives: To summarise the evidence from studies of acute kidney injury (AKI) with regard to the effect of pre-AKI renal function and post-AKI renal function recovery on long-term mortality and renal outcomes, to assess whether these factors should be taken into account in future prognostic studies.

Design/Setting: A systematic review of observational studies listed in Medline and EMBASE from 1990 to October 2012.

Participants: All AKI studies in adults with data on baseline kidney function to identify AKI; with outcomes either stratified by pre-AKI and / or post-AKI kidney function, or described by the timing of the outcomes.

Outcomes: Long-term mortality and worsening chronic kidney disease (CKD).

Results: Of 7385 citations, few studies met inclusion criteria, reported baseline kidney function and stratified by pre or post-AKI function. For mortality outcomes, 3 studies compared patients by pre-AKI renal function and 6 by post-AKI function. For CKD outcomes, 2 studies compared patients by pre-AKI function and 2 by post-AKI function. The presence of CKD pre-AKI (compared to AKI alone) was associated with doubling of mortality and a four- to five-fold increase in CKD outcomes. Non-recovery of kidney function was associated with greater mortality and CKD outcomes in some studies, but findings were inconsistent varying with study design. Two studies also reported that risk of poor outcome reduced over time post-AKI. Meta-analysis was precluded by variations in definitions for AKI, CKD and recovery.

Conclusions: The long term prognosis after AKI varies depending on cause and clinical setting, but it may also, in part, be explained by underlying pre-AKI and post-AKI renal function rather than the AKI episode itself. While carefully considered in clinical practice, few studies address these factors and with inconsistent study design. Future AKI studies should report pre-AKI and post-AKI function consistently as additional factors that may modify AKI prognosis.

For peer review only

Strengths and Limitations of this study

This systematic review followed strict inclusion, exclusion and quality assessment criteria to summarise the available evidence regarding the role of pre-AKI baseline and post-AKI recovery of renal function in long-term AKI outcomes.

Few studies reported long-term AKI outcomes stratified by pre-AKI and post-AKI factors. Quantitative meta-analysis was precluded by heterogeneity in design of included studies.

The review includes papers from Medline and EMBASE up to October 2012. It may potentially have missed studies published in 2013-14 or available in other databases.

For peer review only

For peer review only - http://bmjopen.bmj.com/site/about/guidelines.xhtml

INTRODUCTION

Acute kidney injury (AKI) affects an estimated 13-18% of hospitalised patients[1], frequently under the care of specialties other than nephrology. With the advent of an internationally agreed definition based on changes in serum creatinine and urine output[3], there is now increasing awareness of the poor outcomes suffered by such patients and this has been accompanied by an emphasis on early detection in an effort to improve patient safety and outcomes[1]. Some of the poor outcomes (including increased mortality[3,4] and development of chronic kidney disease (CKD)[5-7]) are increasingly described after hospital discharge, but this is variable and factors associated with long-term prognosis are poorly understood[1]. Recent guidelines from the National Institute for Health and Care Excellence (NICE) call for more studies into acute kidney injury, with non-AKI comparators, but without specifying which factors should be included in study design.

AKI occurs in many different situations and the context is likely to be important both when studying onset of AKI and assessing future outcome risk. In particular, clear knowledge of prior kidney (*pre-AKI* baseline) function is essential to distinguish AKI from CKD. This "baseline function" is typically a creatinine measurement prior to hospitalisation, but may not be available. This can be solved in clinical practice using good clinical judgement, but in epidemiological studies patients with missing baseline values are either assumed to be normal, estimated from other results, or excluded. Baseline function is required for grading AKI severity; as a reference for establishing if recovery is complete; and as a means of stratifying patients with and without *pre-AKI* CKD. It is intuitive that AKI in patients with advanced baseline *pre-AKI* CKD could present and behave differently[8], thus studies without baseline function are at risk of selection bias and misclassification of CKD as AKI.

After an episode of AKI (for instance at hospital discharge or clinic review), the physician also has an opportunity to consider a patient's most recent *post-AKI* kidney function. Accounting for *post-AKI* recovery from AKI (e.g. return of creatinine to within 20% pre-AKI creatinine[3]) may assist future risk assessment. However, the timing of this *post-AKI* assessment may also influence its predictive ability, since clinical course may vary, with both recovery and deterioration possible and not necessarily at a constant rate[9].

Thus, while there is evidence that AKI may have a poor overall prognosis, in this systematic review we seek to evaluate whether previous observational studies have demonstrated the modifying contribution of *pre-AKI* baseline function, *post-AKI* recovery and the timing of outcomes. We also assess whether stratification by these factors should be necessary in future prognostic studies and might provide valuable information to the physician making a risk assessment.

METHODS

A systematic review of observational studies was undertaken in accordance with guidelines for meta-analysis of observational studies in epidemiology (MOOSE)[10]. Medline and EMBASE were searched from January 1990 through October 2012 using Medline subject heading (MeSH) terms and free text for acute kidney injury (acute renal failure, acute kidney disease, acute kidney injury, acute dialysis, RIFLE, AKIN) and prognosis (prognosis, survival, mortality, follow up, progression, chronic kidney

For peer review only - http://bmjopen.bmj.com/site/about/guidelines.xhtml

disease, renal replacement therapy, chronic dialysis, end-stage kidney disease) (see supplementary material). Reference lists of relevant studies and review articles were also searched. There were no language restrictions.

Studies were included if AKI was defined with: creatinine changes within a specified time interval, hospital episode coding for AKI, or the initiation of acute renal replacement therapy (RRT). Studies had to report either mortality or a CKD outcome (development of CKD, progression of CKD severity, or end-stage kidney disease (ESKD)). Studies were included that reported adults (age over 18 years), had at least 50 participants with AKI surviving to hospital discharge, and had follow-up of at least one year. Studies of those with only specialised conditions (e.g. cancer, chemotherapy, transplantation) were excluded.

Two reviewers independently screened titles, abstracts and full papers against the inclusion and exclusion criteria. Where disagreement could not be resolved by discussion, a third author was available, but was not required. One researcher extracted information from the included studies into a data extraction proforma, with confirmation by a second reviewer.

Quality Assessment

Six quality criteria (based on guidelines for assessing the quality of prognostic studies[11]) with a specific focus on the key areas of potential bias and heterogeneity in studies identifying AKI and subsequent outcomes, were used to assess the included studies (figure 1).

Analysis

Study characteristics and quality assessment information were tabulated and described. Multiple papers from the same study dataset were presented together with findings reported from the most complete paper.

Quality criteria 1-3 limited analysis to studies that accurately identified AKI patients and restricted to those who survived the acute event, without significant potential for misclassification of CKD patients. If criteria 1-3 were not met, the study was excluded from further analysis.

Further analysis was limited to studies that analysed patients separately according to one of pre-AKI baseline, post-AKI recovery, or the timing of outcomes by (quality criteria 4-6).

The remaining studies, satisfying criteria 1-3 and one of 4-6, were then summarised in two tables for mortality and CKD outcomes. Clinical setting of AKI was described for each table as follows: intensive care (ITU: patients admitted to ITU, regardless of RRT use); postoperative; cardiac (patients presenting following a myocardial infarction or coronary angiogram); unselected (studies of hospital admissions where the clinical settings above did not describe all patients). Retrospective or prospective design was also reported.

Mortality and CKD outcomes were presented separately, reporting study characteristics, population, exposure, comparators, follow-up and measure of outcome. Multivariate adjusted risk ratios for both survival and CKD outcomes were

For peer review only - http://bmjopen.bmj.com/site/about/guidelines.xhtml

displayed graphically if available. If the timing of outcome was considered in a study this was indicated and described.

RESULTS

The literature search identified 7385 citations. Following review of titles, abstracts and full texts (figure 2), 68 papers describing 61 unique studies were identified (further details summarised in supplementary material). Study size varied, ranging from 61 to 82711 individuals with follow-up from 12 to 142 months. 30 out of 61 studies satisfied quality criteria 1-3, reported either mortality or CKD outcomes (and these studies are summarised in table 1)[12-40]. Notably, 28/61 studies had insufficient pre-AKI data to avoid misclassification of CKD as AKI. Of 30 remaining studies stratification or separation by exclusion of pre-AKI or post-AKI function subgroups was performed in 16/30 mortality studies with 14/30 not conducting separate analyses. It was also performed in 5/30 CKD outcome studies with 25/30 not conducting separate analyses. As noted in table 2, most studies satisfied quality criteria 1-3 by excluding patient groups rather than by stratifying by pre-AKI or post-AKI function. Only three mortality studies stratified by pre-AKI function and six by post-AKI function. Two CKD studies stratified by pre-AKI and two post AKI function. Only two mortality studies[9,20] and one CKD outcome study[9] addressed whether the risk of outcomes changed with increasing time from AKI.

Among these studies, definitions varied: AKI was defined by acute RRT[14], different thresholds for creatinine rise[16,24-27], or hospital episode code[15,19]. CKD was defined by hospital codes[19], low eGFR for CKD cut off (<45ml/min/1.73m^2)[14,21] conventional eGFR for CKD (<60 ml/min/1.73m^2) [16], varying thresholds based on serum creatinine[22,36] or hospital episode code[19]. Definitions of renal recovery also varied: timing from three days[27] to three months[16]; and extent from to within 50% of baseline[21] to within 10% of baseline[15], or below a pre-AKI value[25].

For peer review only

Table 1- Description and Application of Quality Criteria 4-6 in Studies Satisfying Criteria 1-3

			Mortality outcomes reported		CKD outcomes reported		For either outcome
Paper	N	Clinical Setting	by Pre-AKI baseline	by Post-AKI recovery	by Pre-AKI function	by Post-AKI recovery	Timing considered
Hoste 2011 [28]	82	ITU	N	N	-	-	-
Lopes 2010 [29]	61	ITU	N	N	-	-	-
Manns 2003 [30]	66	ITU	N	N	-	-	-
Schiffl 2008 [12]	226	ITU	CKD excluded	Y	-	-	-
Triverio 2009 [18]	95	ITU	Y	Y	-	-	-
Bucaloiu 2012 [13]	1610	Unselected	CKD excluded	Recovered only	CKD excluded	Recovered only	-
Hsu 2009 [14]	782	Unselected	CKD only	N	N	N	-
Ishani 2009 [19]	7197	Unselected	Y by code alone	N	Y by code alone	N	-
Jones 2012 [15]	719	Unselected	CKD excluded	Recovered only	CKD excluded	Recovered only	-
Lafrance 2010 [20]	82711	Unselected	Y	Y	-	-	Sensitivity analysis excluding first six months
Lo 2009 [31]	343	Unselected	N (excluded only if eGFR<45)	N	-	-	-
Ng 2011 [32]	262	Unselected	N	N	-	-	-
Ponte 2008 [22]	177	Unselected	N (Cr>1.4 excluded)	Y	-	-	-
Wald 2012 [33]	41327	Unselected	N	N	N	N	-
Gupta 2005 [34]	143	Cardiac	N	N	-	-	-
Kimura 2010 [35]	81	Cardiac	N	N	-	-	-
Lindsay 2003 [36]	179	Cardiac	Y	N	-	-	-
Maioli 2012 [16]	167	Cardiac	CKD only	Y	-	-	-
Rihal 2002 [37]	185	Cardiac	N	N	-	-	-
Roghi 2008 [38]	106	Cardiac	N	N	-	-	-
Brown 2010 [23]	1886	Postoperative	N	Y	-	-	-
Coca 2010 [24]	6257	Postoperative	N	Y	-	-	-
Ishani 2011 [9]	4053*	Postoperative	N	N	N	N	Changing hazard ratios for mortality and CKD outcomes in time intervals
Kheterpal 2007 [17]	101	Postoperative	CKD excluded	N	-	-	-
Loef 2005 [25]	145	Postoperative	N	Y	-	-	-
Luckraz 2005 [39]	53	Postoperative	N	N	-	-	-
Mehta 2010 [26]	2083	Postoperative	N (excluded only Cr>2)	Y	-	-	-
Swaminathan 2010 [40]	1113	Postoperative	N	N[$]	-	-	-
Van Kuijk 2010 [27]	120	Postoperative	N	N	CKD excluded	Y	-
Wu 2011 [21]	4393	Postoperative	Y by GFR>/<45	Y	Y by GFR>/<45	Y	-

N = outcome addressed but not per quality criterion, - = outcome not addressed by study, * = 19779 in total, 4053 of which had >50% cr rise, [$] = outcomes by rate of fall of creatinine over a 24 hours rather than a recovery variable, Cr = creatinine

For peer review only - http://bmjopen.bmj.com/site/about/guidelines.xhtml

Table 2 –Studies of Mortality Outcomes in AKI by pre and post-AKI Kidney Function

Mortality	N	Clinical Setting	Design	AKI Exposure	Comparator	Follow Up (*mean /median)	Pre-AKI baseline separation	Post-AKI recovery separation	Recovery definition	Findings (*statistically significant p<0.05)
Schiffl 2008 [12]	226	ITU	Cohort	Acute RRT	None	5 years	CKD excluded	Y	Within 10% baseline	Mortality 83% without recovery, 33% with recovery<br>HR without vs with recovery 4.1* (no non-AKI comparator)
Triverio 2009 [18]	95	ITU	Cohort	Acute RRT	None	3 years	Y	Y	eGFR>60	Mortality 50% if baseline CKD, 29% if eGFR<60 at discharge, 18% if no renal impairment before or after AKI
Bucaloiu 2012 [13]	1610	Unselected	Cohort	Cr rise >50%	No AKI	*3.3 years	CKD excluded	Recovered only	Within 10% baseline	HR Mortality 1.48*
Hsu 2009 [14]	782	Unselected	Cohort	Acute RRT	CKD with no RRT	4 years	CKD only (eGFR<45)	N	-	HR composite endpoint of ESKD or Mortality 1.3*
Ishani 2009 [19]	7197	Unselected	Cohort	Code	No AKI or CKD code	2 years	Y by code alone	N	-	HR Mortality vs no AKI or CKD<br>AKI and CKD 3.24*, AKI 2.48*, CKD 1.45*
Jones 2012 [15]	719	Unselected	Cohort	Code	No AKI	*2.5 years	CKD excluded	Recovered only	Within 10% Baseline	HR Mortality 1.08
Lafrance 2010 [20]	82711	Unselected	Cohort	Cr rise >50%	No AKI	*2.34 years	Y	Recovered only in a subanalysis	Within 10% baseline	HR Mortality for AKI vs no AKI in 90d survivors 1.41*<br>Subgroup of 6 month survivors 1.13*<br>Subgroup with recovery 1.47*
Ponte 2008 [22]	177	Unselected	Cohort	Cr rise from <1.4mg/dl to >2	None	*7.2 years	CKD excluded (Cr>1.4mg/dl)	Y	Cr <1.4mg/dl	10 year mortality 40% with recovery, 57% without recovery
Lindsay 2003 [36]	179	Cardiac	Cohort	Cr rise 50% from <1.2mg/dl	No AKI	1 year	CKD excluded (cr>1.2mg/dl)	N	-	1year mortality 9.5% AKI, 2.7% no-AKI
Maioli 2012 [16]	167	Cardiac	Cohort	Cr rise (0.5mg/dl by 3 days)	No AKI	*3.8 years	CKD only (eGFR<60)	Y	Within 25% baseline at 3 months	HR Mortality for AKI with recovery1.3*, without recovery 2.3*
Brown 2010 [23]	1886	Postoperative	Cohort	Cr rise 0.3mg/dl or 50%	No AKI	*2.6 years	N	Y	Number of days AKI definition met	HR Mortality for AKI vs no AKI by AKI Duration<br>1-2d 1.51*, 3-6d 1.74*, >7d 3.45*, persistent 5.75*
Coca 2010 [24]	6257	Postoperative	Cohort	Cr rise 0.3mg/dl or 50%	No AKI	*3.8 years	N	Y	Number of days AKI definition met	HR Mortality for AKI vs no AKI by AKI Duration<br><2d 1.15*, 3-6d 1.5*,>7d any duration with RRT 2.10*
Kheterpal 2007 [17]	101	Postoperative	Cohort	Cr clearance fall to <50	No AKI	1 year	CKD excluded	N	-	1 year mortality 12% AKI, 9% no AKI
Loef 2005 [25]	145	Postoperative	Cohort	Cr rise 25%	No AKI	100 months	N	Y	"improved to or below the preoperative level"	HR Mortality for AKI 1.63*, AKI with recovery 1.66*, AKI without recovery 1.72*, Non-recovery vs recovery 1.22 (not significant)
Mehta 2010 [26]	2083	Postoperative	Cohort	Cr rise 50% or 0.7mg/dl	No AKI	*7 years	N	Y	AKI definition no longer met at 7 days	HR mortality for AKI with recovery 1.21*, without recovery 1.47*
Wu 2011 [21]	4393	Postoperative	Cohort	Cr rise >50%	No AKI and no CKD	*4.76 years	Y by GFR>/<45	Y	Within 50% baseline	HR Mortality for AKI only 1.94*, CKD only 2.64*, AKI and CKD 3.28*<br><br>Stratified by recovery<br>AKI and CKD with recovery 3.0*, without recovery 4.59*<br>AKI only with recovery 1.96*, without recovery 2.18 *<br>CKD without AKI 2.59*

N = outcome addressed but not per quality criterion, - = outcome not addressed by study, ITU=intensive therapy unit; RRT=renal replacement therapy; AKI=acute kidney injury; CKD=chronic kidney disease; eGFR=estimated glomerular filtration rate; HR=hazard ratios; ESKD=end-stage kidney disease (dialysis>90days); Cr=serum creatinine

For peer review only - http://bmjopen.bmj.com/site/about/guidelines.xhtml

Table 3 –Studies of CKD Outcomes in AKI by pre and post-AKI Kidney Function

Progression	N	Clinical Setting	Design	AKI Exposure	Comparator	Follow-up (*mean /median)	Pre-AKI baseline separation	Post-AKI recovery separation	Recovery definition (discharge unless otherwise stated)	Findings (*statistically significant p<0.05)
Bucaloiu 2012 [13]	1610	Unselected	Cohort	Cr rise >50%	No AKI	*3.3 years	CKD excluded	Recovered only	Within 10% baseline	Development of New CKD Stage 3 per 1000 person-year<br>28.1 AKI vs 13.1 no AKI (HR 1.91*)
Ishani 2009 [19]	7197	Unselected	Cohort	Code	No AKI or CKD code	2 years	Y by code alone	N	-	ESKD/1000 person-year (HR vs no code)<br>AKI and CKD 79.45 (HR 41.2*)<br>AKI 24.52 (HR 13*)<br>CKD only 19.88 (HR 8.43*)<br>No Code 2.08 (reference)
Jones 2012 [15]	719	Unselected	Cohort	Code	No AKI	*2.5 years	CKD excluded	Recovered only	Within 10% Baseline	HR for new CKD Stage 3 (15% of AKI vs 3% of no AKI patients)<br>3.82*
van Kuijk 2010 [27]	493	Postoperative	Cohort	10% Cr change by day 2 post-op	No AKI	*5 years	CKD excluded	Y	Within 10% baseline at day 3	Development of New CKD<br>11% if no AKI, 32% if AKI recovered (HR 3.4*), 36% if AKI without recovery (HR3.6*)
Wu 2011 [21]	4393	Postoperative	Cohort	Cr rise (RIFLE, or 50% rise if previous CKD)	No AKI and no CKD	*4.76 years	Y by GFR>/<45	Y	Within 50% baseline	Development of ESKD (vs no code)<br>AKI only 4.64*, CKD only 40.86*, AKI and CKD 91.69*<br><br>Stratified by recovery<br>AKI + CKD + without recovery 212.73*<br>AKI + CKD + recovery 74.07*<br>AKI only + without recovery 60.95*<br>AKI only + recovery 4.5*

N = outcome addressed but not per quality criterion, - = outcome not addressed by study; ITU=intensive therapy unit; RRT=renal replacement therapy; AKI=acute kidney injury; CKD=chronic kidney disease; eGFR=estimated glomerular filtration rate; HR=hazard ratios; ESKD=end-stage renal disease (dialysis>90days); Cr=serum creatinine

For peer review only - http://bmjopen.bmj.com/site/about/guidelines.xhtml

Mortality findings

There were 16 mortality studies (table 2). Follow up ranged from 1 to 7 years and mortality up to 83% for AKI patients at 5 years. AKI was associated with increased mortality in all but one study[15] regardless of pre-AKI baseline (HR 1.08 to 4.59) (figure 3))or recovery of renal function (HR1.08 to 5.75) (figure 4). Five studies did not report non-AKI comparators[12,18,22] or risk ratios[17,36].

Of the three studies stratified by pre-AKI baseline function, in two the overall prognosis was worse in AKI patients with prior CKD with doubling of hazard ratios with respect to a non-AKI non-CKD comparator[16,21]. However, in a third study, where comparators were also stratified by eGFR, the independent mortality risk from AKI diminished with advancing CKD[20]. Six studies compared mortality stratifying either by post AKI recovery (four studies)[16,21, 25, 26] or by AKI duration (two studies)[23,24]. All but one[16] were postoperative studies with different thresholds for defining recovery. Risk from incomplete recovery varied from minimal with overlapping confidence intervals[16,21,25, 26] to greater than double when AKI lasted more than one week[23,24]. Only one study combined stratification by both pre-AKI baseline and post-AKI recovery (defining recovery to 50% baseline, CKD as eGFR<45 ml/min/1.73m^2) with both non-recovery and baseline CKD adding to mortality risk[21].

CKD Outcomes

In five studies, there was marked variation in CKD outcomes following AKI (HR1.91 to 213) depending on pre-AKI baseline and post-AKI recovery (table 3 and figure 5)[13,15,21,19,27]. In the two studies stratifying by pre-AKI function, AKI with baseline CKD was associated with a substantially greater (four-fivefold) risk of ESKD than AKI in cases without prior CKD[19,21] although notably one study defined CKD at eGFR <45ml/min/1.73m^2 [21] and the other by a hospital code[19]. The two studies stratifying by post-AKI function had different findings. In one postoperative study[27] there was no association between non-recovery post-AKI (vs patients with recovery) and additional CKD progression, when recovery was measured at day three[27], but association was substantial (five-tenfold) in the other study where recovery was defined at hospital discharge[21]. Again only one study stratified by both baseline and recovery, finding both associated with poorer prognosis[21].

Timing of Outcomes

Two studies considered whether hazard ratios changed over time following the AKI episode[9,20]. Lafrance et al. (2010)[20], performed a sensitivity analysis of their study of AKI survivors and mortality, limiting analysis from 90 day to six month AKI survivors. The mortality HR (AKI versus non-AKI) fell from 1.41(1.39-1.43) in 90 day survivors to 1.13 (1.11-1.14) in six month survivors[20]. Ishani et al. (2011)[9] reported changes in both hazard ratio of mortality and CKD outcomes from AKI (vs no AKI) when dividing follow up into six month time discrete intervals. For both mortality and CKD outcomes, greatest relative risk (vs non-AKI) was in the first six months, with marked attenuation of risk (but still statistically significant) from one year[9]. While recognising that risk may not be constant, the study did not also consider the importance of stratifying patients by pre-AKI baseline or post-AKI recovery.

DISCUSSION

The introduction of a global definition for AKI has led to growing interest in the high incidence and poor outcomes. In this review of 7385 citations, there were only 30

For peer review only - http://bmjopen.bmj.com/site/about/guidelines.xhtml

studies that met the initial quality criteria for selecting AKI patients with sufficient efforts to avoid misclassifying CKD. Of these studies only three mortality studies stratified by pre-AKI, six by post-AKI function, two CKD outcome studies by pre-AKI and two by post AKI function. Only one study considered both pre-AKI and post-AKI function. Only two studies considered the change in risk over time from AKI.

As with two previous reviews, we note that AKI is associated with overall poorer mortality and CKD outcomes[4,6], but with substantial variation in the magnitude of risk. We were transparent about the variation in definitions and clinical setting and identified heterogeneity as a problem with the design of AKI studies to date. The two previous reviews included studies with incomplete or estimated data on baseline kidney function[4,6], but in studies with available data we found that outcomes were modulated by both pre-AKI baseline and post-AKI renal recovery. Unfortunately, these studies varied in definitions for AKI, recovery and follow up preventing pooled meta-analysis. While proteinuria and oliguria may also be important factors, research on their association with long term prognosis is sparse and we therefore focused on the course of serum creatinine prior and subsequent to the AKI episode.

Pre-AKI baseline CKD was associated with doubling of mortality outcomes and four- to five-fold increased risk of CKD outcomes compared to non-AKI non-CKD patients. Unfortunately, only one study chose CKD comparators for AKI patients at each level of pre-AKI function, finding that the additional risk from AKI diminished in those already at high risk due to advanced baseline CKD[20]. It is likely the mortality and ESKD risk in advanced CKD is already high and less influenced by AKI.

Non-recovery of kidney function was associated with greater mortality and CKD outcomes in some studies, but the finding was inconsistent and dependent on study design. Studies with a less stringent threshold[21] and later point of assessment[16] showed a poorer prognosis with non-recovery, highlighting the significance of arbitrary thresholds in study design. We also note a further study outside the time period in this review reporting a cohort with poorer outcomes with non-recovery after AKI, in line with our overall findings[41].

Increased risk of poor outcomes following AKI may be greatest early post-AKI, and therefore the timing of clinical assessment is important. Despite this, most studies followed a "proportional hazards" assumption (i.e. that the relative risk of AKI vs non-AKI comparator was constant over time), but this may not hold. Only two studies considered the timing of assessment post-AKI and found that both additional risk of mortality and renal progression may diminish over time[9,20], but neither combined this with adjustment or stratification by post-AKI recovery. Both factors are required to assess if the rate of CKD progression is more rapid in AKI than non-AKI (but baseline CKD) comparators with equal kidney function at the time of hospital discharge. Thus, understanding of how mortality and renal progression risks change over time remains inadequately addressed.

Overall while our findings broadly agree with previous reviews[4,6], we found that some of the variation in AKI prognosis may be explained by pre-AKI CKD and post-AKI non-recovery which both lead to a poorer prognosis. This is of great relevance given that these factors were infrequently addressed by stratification, and 28/61 studies had

For peer review only - http://bmjopen.bmj.com/site/about/guidelines.xhtml

insufficient pre-AKI data to minimise misclassification between CKD and AKI. The relationships are complex with diminishing additional risk with advancing pre-AKI CKD. We also noted a non-linear clinical post-AKI course, with risk prediction dependent on the time point at which risk is assessed, necessitating fixed follow up points to prevent overestimation of risk in a cohort with shorter follow up. Importantly, few studies assessed these factors, using different definitions, and with only one study assessing both pre-AKI and post-AKI factors. This review included studies up to October 2012 and in the near future it is likely that AKI registries with additional routine outcome data will become available. Therefore there is now an opportunity to ensure that a concordance exists in definitions and reporting of future AKI studies before this review is revisited.

The NICE guidelines call for further studies of long term AKI prognosis but without specific detail on which factors should be included. We suggest that future studies should adopt consistent definitions, incorporate baseline and recovery function and consider the timing of outcomes during follow up. We therefore suggest that future long term prognostic studies should adopt the KDIGO definition for identifying and stratifying AKI[2], the KDIGO CKD stages for stratifying AKI and non-AKI comparator groups by pre-AKI eGFR[42], the UK Renal Association definition of renal recovery for stratifying recovery (within 20% of baseline)[3] and fixed interim points for establishing recovery (7, 30, 90 days) and excluding early outcomes (up to 30, 90, and 180 days).

CONCLUSION

AKI has a poor, but variable prognosis influenced by clinical setting, underlying cause and comorbidity. There is a recognised need to understand which patients are at greater long-term risk and when AKI may carry additional risk beyond the underlying illness. In this review, pre-AKI CKD was associated with doubling of mortality outcomes and a four-fivefold increase in renal outcomes. Non-recovery of kidney function may also affect prognosis, but the magnitude depended on both recovery definition and the timing of assessment. These factors are already considered by physicians in clinical practice, but unfortunately we found that few prognostic studies have addressed them. The heterogeneity of studies and accompanying lack of a clear consistent pattern of effect limit their clinical interpretation. As pre and post-AKI renal function may influence clinical practice, we suggest that consistent reporting of these factors is needed in future prognostic studies to establish how they modify AKI prognosis. This will inform both the clinician and future research.

Contributorship

SS, AM, NF and CB contributed to the design of the review, SS and MM acquired the data. SS, AM and CB contributed to analysis. SS drafted and SS, AM, NF and CB critically revised the intellectual content of the work. All authors have given final approval for this paper to be published.

Competing Interests

None declared.

Funding

SS is supported by a research training fellowship from the Wellcome Trust.

For peer review only - http://bmjopen.bmj.com/site/about/guidelines.xhtml

Acknowledgements
None declared.

Data Sharing
No additional data available.

REFERENCES

1. Ftouh S, Lewington A, on behalf of the Acute Kidney Injury Guideline Development Group convened by the National Clinical Guidelines Centre and commissioned by the National Institute for Health and Care Excellence,in association with The Royal College of Physicians' Clinical Effectiveness and Evaluation Unit. Prevention, detection and management of acute kidney injury: concise guideline. *Clinical Medicine* 2014, 14:61-5.
2. Kidney Disease: Improving Global Outcomes (KDIGO) Acute Kidney Injury Work Group. KDIGO Clinical Practice Guideline for Acute Kidney Injury. *Kidney Int* 2012, Suppl2:1-138.
3. Lewington A, Kanagasundaram S. Clinical Practice Guidelines. Acute Kidney Injury. 5th ed.: UK Renal Association; 2011.
4. Coca SG, Yusuf B, Shlipak MG, et al. Long-term risk of mortality and other adverse outcomes after acute kidney injury: a systematic review and meta-analysis. *Am J Kidney Dis* 2009, 53:961-973.
5. Bydash JR, Ishani A. Acute kidney injury and chronic kidney disease: a work in progress. *Clin J Am Soc Nephrol* 2011, 6:2555-2557.
6. Coca SG, Singanamala S, Parikh CR. Chronic kidney disease after acute kidney injury: a systematic review and meta-analysis. *Kidney Int* 2012, 81:442-448.
7. Chawla LS, Kimmel PL. Acute kidney injury and chronic kidney disease: an integrated clinical syndrome. *Kidney Int* 2012, 82:516-524.
8. James MT, Hemmelgarn BR, Wiebe N, et al. Glomerular filtration rate, proteinuria, and the incidence and consequences of acute kidney injury: a cohort study. *Lancet* 2010, 376:2096-2103.
9. Ishani A, Nelson D, Clothier B, et al. The magnitude of acute serum creatinine increase after cardiac surgery and the risk of chronic kidney disease, progression of kidney disease, and death. *Arch Intern Med* 2011, 171:226-233.
10. Stroup DF, Berlin JA, Morton SC, et al. Meta-analysis of observational studies in epidemiology: a proposal for reporting. Meta-analysis Of Observational Studies in Epidemiology (MOOSE) group. JAMA 2000, 283(15):2008-2012.
11. Hayden JA, Cote P, Bombardier C. Evaluation of the quality of prognosis studies in systematic reviews. *Ann Intern Med* 2006, 144:427-437.
12. Schiffl H, Fischer R. Five-year outcomes of severe acute kidney injury requiring renal replacement therapy. *Nephrol Dial Transplant* 2008, 23:2235-2241.
13. Bucaloiu ID, Kirchner HL, Norfolk ER, et al. Increased risk of death and de novo chronic kidney disease following reversible acute kidney injury. *Kidney Int* 2012, 81:477-485.
14. Hsu CY, Chertow GM, McCulloch CE, et al. Nonrecovery of kidney function and death after acute on chronic renal failure. *Clin J Am Soc Nephrol* 2009, 4:891-898.

For peer review only - http://bmjopen.bmj.com/site/about/guidelines.xhtml

15. Jones J, Holmen J, De Graauw J, et al. Association of complete recovery from acute kidney injury with incident CKD stage 3 and all-cause mortality. *Am J Kidney Dis* 2012, 60:402-408.
16. Maioli M, Toso A, Leoncini M, et al. Persistent renal damage after contrast-induced acute kidney injury: incidence, evolution, risk factors, and prognosis. *Circulation* 2012, 125:3099-3107.
17. Kheterpal S, Tremper KK, Englesbe MJ, et al. Predictors of postoperative acute renal failure after noncardiac surgery in patients with previously normal renal function. *Anesthesiology* 2007, 107:892-902.
18. Triverio PA, Martin PY, Romand J, et al. Long-term prognosis after acute kidney injury requiring renal replacement therapy. *Nephrol Dial Transplant* 2009, 24:2186-2189.
19. Ishani A, Xue JL, Himmelfarb J, et al. Acute kidney injury increases risk of ESRD among elderly. *J Am Soc Nephrol* 2009, 20:223-228
20. Lafrance JP, Miller DR. Acute kidney injury associates with increased long-term mortality. *J Am Soc Nephrol* 2010, 21:345-352.
21. Wu VC, Huang TM, Lai CF, et al. Acute-on-chronic kidney injury at hospital discharge is associated with long-term dialysis and mortality. *Kidney Int* 2011, 80:1222-1230.
22. Ponte B, Felipe C, Muriel A, et al. Long-term functional evolution after an acute kidney injury: a 10-year study: *Nephrol Dial Transplan* 2008, 23:3859-3866.
23. Brown JR, Kramer RS, Coca SG, et al. Duration of acute kidney injury impacts long-term survival after cardiac surgery. *Ann Thorac Surg* 2010, 90:1142-1148.
24. Coca SG, King JT,Jr, Rosenthal RA, et al. The duration of postoperative acute kidney injury is an additional parameter predicting long-term survival in diabetic veterans. *Kidney Int* 2010, 78:926-933.
25. Loef BG, Epema AH, Smilde TD, et al. Immediate postoperative renal function deterioration in cardiac surgical patients predicts in-hospital mortality and long-term survival. *J Am Soc Nephrol* 2005, 16:195-200.
26. Mehta RH, Honeycutt E, Patel UD, et al. Impact of recovery of renal function on long-term mortality after coronary artery bypass grafting. *Am J Cardiol* 2010, 106:1728-1734.
27. van Kuijk JP, Flu WJ, Chonchol M, et al. Temporary perioperative decline of renal function is an independent predictor for chronic kidney disease. *Clin J Am Soc Nephrol* 2010, 5:1198-1204.
28. Hoste EA, Doom S, De Waele J, et al. Epidemiology of contrast-associated acute kidney injury in ICU patients: a retrospective cohort analysis. *Intensive Care Med* 2011, 37:1921-1931.
29. Lopes JA, Fernandes P, Jorge S, et al. Long-term risk of mortality after acute kidney injury in patients with sepsis: a contemporary analysis. *BMC Nephrology* 2010, 11:9.
30. Manns B, Doig CJ, Lee H, et al. Cost of acute renal failure requiring dialysis in the intensive care unit: clinical and resource implications of renal recovery. *Crit Care Med* 2003, 31:449-455.
31. Lo LJ, Go AS, Chertow GM, et al. Dialysis-requiring acute renal failure increases the risk of progressive chronic kidney disease. *Kidney Int* 2009, 76:893-899.

For peer review only - http://bmjopen.bmj.com/site/about/guidelines.xhtml

32. Ng KP, Chanouzas D, Fallouh B, et al. Short and long-term outcome of patients with severe acute kidney injury requiring renal replacement therapy. *QJM* 2012, 105:33-39.
33. Wald R, Quinn RR, Adhikari NK, et al. Risk of chronic dialysis and death following acute kidney injury. *Am J Med* 2012, 125:585-593.
34. Gupta R, Gurm HS, Bhatt DL, et al. Renal failure after percutaneous coronary intervention is associated with high mortality. *Catheter Cardiovasc Interv* 2005, 64:442-448.
35. Kimura T, Obi Y, Yasuda K, et al. Effects of chronic kidney disease and post-angiographic acute kidney injury on long-term prognosis after coronary artery angiography. *Nephrol Dial Transplan* 2011, 26:1838-1846.
36. Lindsay J, Apple S, Pinnow EE, et al. Percutaneous coronary intervention-associated nephropathy foreshadows increased risk of late adverse events in patients with normal baseline serum creatinine. *Catheter Cardiovasc Interv* 2003, 59:338-343.
37. Rihal CS, Textor SC, Grill DE, et al. Incidence and prognostic importance of acute renal failure after percutaneous coronary intervention. *Circulation* 2002, 105:2259-2264.
38. Roghi A, Savonitto S, Cavallini C, et al. Impact of acute renal failure following percutaneous coronary intervention on long-term mortality. *J Cardiovasc Med* 2008, 9:375-381.
39. Luckraz H, Gravenor MB, George R, et al. Long and short-term outcomes in patients requiring continuous renal replacement therapy post cardiopulmonary bypass. *Eur J Cardiothorac Surg* 2005, 27:906-909.
40. Swaminathan M, Hudson CC, Phillips-Bute BG, et al. Impact of early renal recovery on survival after cardiac surgery-associated acute kidney injury. *Ann Thorac Surg* 2010, 89:1098-1104.
41. Pannu N, James M, Hemmelgarn B, et al. Association between AKI, Recovery of Renal Function, and Long-Term Outcomes after Hospital Discharge. *Clin J Am Soc Nephrol* 2013, 8:194-202.
42. Stevens PE, Levin A, Kidney Disease: Improving Global Outcomes Chronic Kidney Disease Guideline Development Work Group Members. Evaluation and management of chronic kidney disease: synopsis of the kidney disease: improving global outcomes 2012 clinical practice guideline. Ann Intern Med 2013, 158(11):825-830.

For peer review only

For peer review only - http://bmjopen.bmj.com/site/about/guidelines.xhtml

Figure 1- Quality Assessment Criteria

Figure 2- Study Selection and Quality Assessment

Figure 3 - Mortality – By Pre-AKI Baseline

w/o rec = without recovery, with rec = with recovery, HR = hazard ratio, CI = confidence interval

Figure 4 - Mortality – By Post AKI Recovery

w/o rec = without recovery, with rec = with recovery, HR = hazard ratio, CI = confidence interval

Figure 5 - CKD Outcomes

w/o rec = without recovery, with rec = with recovery, HR = hazard ratio, CI = confidence interval

For peer review only

For peer review only - http://bmjopen.bmj.com/site/about/guidelines.xhtml

Long Term Prognosis after Acute Kidney Injury (AKI) - What is the Role of Baseline Kidney Function and Recovery? A Systematic Review

Simon Sawhney[1,2*] MBChB
simon.sawhney@abdn.ac.uk

Mhairi Mitchell[1] MBChB
m.mitchell.08@aberdeen.ac.uk

Angharad Marks[1,2] MBBCh
a.marks@abdn.ac.uk

Nick Fluck[2] MBBS
nfluck@nhs.net

Corrinda Black[1] MBChB
corri.black@abdn.ac.uk

[1]Division of Applied Health Sciences, University of Aberdeen
[2]Renal Unit,Ward 108 Aberdeen Royal Infirmary

[*]Correspondence to:
Simon Sawhney, Division of Applied Health Sciences, University of Aberdeen, Polwarth Building, Foresterhill, Aberdeen AB25 2ZD, UK

Key words:
Acute Kidney Injury
Chronic Renal Insufficiency
Epidemiology
Systematic Review

Word count: ~~2591~~ 2705 words

For peer review only - http://bmjopen.bmj.com/site/about/guidelines.xhtml

ABSTRACT

Objectives: To summarise the evidence from studies of acute kidney injury (AKI) with regard to the effect of level of pre-AKI renal function and post-AKI renal function recovery on long-term mortality and renal outcomes, to assess whether these factors should be taken into account in future prognostic studies.

Design/Setting: A systematic review of observational studies listed in Medline and EMBASE from 1990 to presentOctober 2012.

Participants: All AKI studies in adults with data on baseline kidney function to identify AKI; with outcomes either stratified by pre-AKI and / or post-AKI kidney function, or described by the timing of the outcomes.

Outcomes: Long-term mortality and worsening chronic kidney disease (CKD).

Results: Of 7385 citations, 16 studies of mortality and 5 studies of CKD outcomes met inclusion quality criteria. Three studies of mortality stratified by pre-AKI and six by post-AKI renal function. Two studies of CKD stratified by pre-AKI and two by post-AKI function. Of 7385 citations, few studies met inclusion criteria, reported baseline kidney function and stratified by pre or post-AKI function. For mortality outcomes, 3 studies compared patients by pre-AKI renal function and 6 by post-AKI function. For CKD outcomes, 2 studies compared patients by pre-AKI function and 2 by post-AKI function. The presence of CKD pre-AKI (compared to AKI alone) was associated with doubling of mortality and a four- to five-fold increase in CKD outcomes. Non-recovery of kidney function was associated with greater mortality and CKD outcomes in some studies, but findings were inconsistent varying with study design. Two studies also reported that risk of poor outcome reduced over time post-AKI. Meta-analysis was precluded by variations in definitions for AKI, CKD and recovery.

Conclusions: While carefully considered in clinical practice, few heterogeneous long-term prognostic studies address pre-AKI and post-AKI renal function. Variable AKI prognosis may in part be explained by these factors. Future studies should incorporate pre-AKI and post-AKI measurements with consistent definitions to establish how to predict future risk following AKI.

The long term prognosis after AKI varies depending on cause and clinical setting, but it may also, in part, be explained by underlying pre-AKI and post-AKI renal function rather than the AKI episode itself. While carefully considered in clinical practice, few studies address these factors and with inconsistent study design. Future AKI studies should report pre-AKI and post-AKI function consistently as additional factors that may modify AKI prognosis.

Formatted: Font: Not Bold, No underline

Formatted: Font: Not Bold, No underline

For peer review only - http://bmjopen.bmj.com/site/about/guidelines.xhtml

Strengths and Limitations of this study

This systematic review followed strict inclusion, exclusion and quality assessment criteria to summarise the available evidence regarding the role of pre-AKI baseline and post-AKI recovery of renal function in long-term AKI outcomes.

Few studies reported long-term AKI outcomes stratified by pre-AKI and post-AKI factors. Quantitative meta-analysis was precluded by heterogeneity in design of included studies.

The review includes papers from Medline and EMBASE up to October 2012. It may potentially have missed studies published in 2013-14 or available in other databases.

For peer review only

INTRODUCTION

Acute kidney injury (AKI) affects an estimated 13-18% of hospitalised patients[1], frequently under the care of specialties other than nephrology. With the advent of an internationally agreed definition based on changes in serum creatinine and urine output[3], there is now increasing awareness of the poor outcomes suffered by such patients and this has been accompanied by an emphasis on ~~screening~~ early detection in an effort to improve patient safety and outcomes[1]. Some of the poor outcomes (including increased mortality[3,4] and development of chronic kidney disease (CKD)[5-7]) are increasingly described after hospital discharge, but this is variable and factors associated with long-term prognosis are poorly understood[1]. Recent guidelines from the National Institute ~~of Clinical~~for Health and Care Excellence (NICE) call for more studies into acute kidney injury, with non-AKI comparators, but without specifying which factors should be included in study design.

AKI occurs in many different situations and the context is likely to be important both when studying onset of AKI and assessing future outcome risk. In particular, clear knowledge of prior kidney (*pre-AKI* baseline) function is essential to distinguish AKI from CKD. This "baseline function" is typically a creatinine measurement prior to hospitalisation, but may not be available~~ or may be estimated if absent~~. This can be solved in clinical practice using good clinical judgement, but in epidemiological studies patients with missing baseline values are either assumed to be normal, estimated from other results, or excluded. Baseline function is required for grading AKI severity; as a reference for establishing if recovery is complete; and as a means of stratifying patients with and without *pre-AKI* CKD. It is intuitive that AKI in patients with advanced baseline *pre-AKI* CKD could present and behave differently[8], thus studies without baseline function are at risk of selection bias and misclassification of CKD as AKI.

After an episode of AKI (for instance at hospital discharge or clinic review), the physician also has an opportunity to consider a patient's most recent *post-AKI* kidney function. Accounting for *post-AKI* recovery from AKI (e.g. return of creatinine to within 20% pre-AKI creatinine[3]) may assist future risk assessment. However, the timing of this *post-AKI* assessment may also influence its predictive ability, since clinical course may vary, with both recovery and deterioration possible and not necessarily at a constant rate[9].

Thus, while there is evidence that AKI may have a poor overall prognosis, in this systematic review we seek to evaluate whether previous observational studies have demonstrated the modifying contribution of *pre-AKI* baseline function, *post-AKI* recovery and the timing of outcomes. We also assess whether stratification by these factors should be necessary in future prognostic studies and might provide valuable information to the physician making a risk assessment.

METHODS

A systematic review of observational studies was undertaken in accordance with guidelines for meta-analysis of observational studies in epidemiology (MOOSE)[10]. Medline and EMBASE were searched from January 1990 through October 2012 using Medline subject heading (MeSH) terms and free text for acute kidney injury (acute renal failure, acute kidney disease, acute kidney injury, acute dialysis, RIFLE, AKIN)

For peer review only - http://bmjopen.bmj.com/site/about/guidelines.xhtml

and prognosis (prognosis, survival, mortality, follow up, progression, chronic kidney disease, renal replacement therapy, chronic dialysis, end-stage kidney disease) (see supplementary material). Reference lists of relevant studies and review articles were also searched. There were no language restrictions.

Studies were included if AKI was defined with: creatinine changes within a specified time interval, hospital episode coding for AKI, or the initiation of acute renal replacement therapy (RRT). Studies had to report either mortality or a CKD outcome (development of CKD, progression of CKD severity, or end-stage kidney disease (ESKD)). Studies were included that reported adults (age over 18 years), had at least 50 participants with AKI surviving to hospital discharge, and had follow-up of at least one year. Studies of those with only specialised conditions (e.g. cancer, chemotherapy, transplantation) were excluded.

Two reviewers independently screened titles, abstracts and full papers against the inclusion and exclusion criteria. Where disagreement could not be resolved by discussion, a third author was available, but was not required. One researcher extracted information from the included studies into a data extraction proforma, with confirmation by a second reviewer.

Quality Assessment

Six quality criteria (based on guidelines for assessing the quality of prognostic studies[11]) with a specific focus on the key areas of potential bias and heterogeneity in studies identifying AKI and subsequent outcomes, were used to assess the included studies (figure 1).

Analysis

Study characteristics and quality assessment information were tabulated and described. Multiple papers from the same study dataset were presented together with findings reported from the most complete paper.

Quality criteria 1-3 limited analysis to studies that accurately identified AKI patients and restricted to those who survived the acute event, without significant potential for misclassification of CKD patients. If criteria 1-3 were not met, the study was excluded from further analysis.

Further analysis was limited to studies that analysed patients separately according to one of pre-AKI baseline, post-AKI recovery, or the timing of outcomes by (quality criteria 4-6-).

The remaining studies, satisfying criteria 1-3 and one of 4-6, were then summarised in two tables for mortality and CKD outcomes. Clinical setting of AKI was described for each table as follows: intensive care (ITU: patients admitted to ITU, regardless of RRT use); postoperative; cardiac (patients presenting following a myocardial infarction or coronary angiogram); unselected (studies of hospital admissions where the clinical settings above did not describe all patients). Retrospective or prospective design was also reported.

Mortality and CKD outcomes were presented separately, reporting study characteristics, population, exposure, comparators, follow-up and measure of

For peer review only - http://bmjopen.bmj.com/site/about/guidelines.xhtml

outcome. Multivariate adjusted risk ratios for both survival and CKD outcomes were displayed graphically if available. If the timing of outcome was considered in a study this was indicated and described.

RESULTS

The literature search identified 7385 citations. Following review of titles, abstracts and full texts (figure 2), 68 papers describing 61 unique studies were identified (further details summarised in supplementary material). Study size varied, ranging from 61 to 82711 individuals with follow-up from 12 to 142 months. 30 out of 61 studies satisfied quality criteria 1-3, reported either mortality or CKD outcomes (and these studies are summarised in table 1)[12-40]. Notably, 28/61 studies had insufficient pre-AKI data to avoid misclassification of CKD as AKI. Of 30 remaining studies stratification or separation by exclusion of pre-AKI or post-AKI function subgroups was performed in 16/30 mortality studies with 14/30 not conducting separate analyses. It was also performed in 5/30 CKD outcome studies with 25/30 not conducting separate analyses. As noted in table 2, most studies satisfied quality criteria 1-3 by excluding patient groups rather than by stratifying by pre-AKI or post-AKI function. Only three mortality studies stratified by pre-AKI function and six by post-AKI function. Two CKD studies stratified by pre-AKI and two post AKI function. Only two mortality studies[9,20] and one CKD outcome study[9] addressed whether the risk of outcomes changed with increasing time from AKI.

Among these studies, definitions varied: AKI was defined by acute RRT[14], different thresholds for creatinine rise[16,24-27], or hospital episode code[15,19]. CKD was defined by hospital codes[19], low eGFR for CKD cut off (<45ml/min/1.73m^2)[14,21] conventional eGFR for CKD (<60 ml/min/1.73m^2) [16], varying thresholds based on serum creatinine[22,36] or hospital episode code[19]. Definitions of renal recovery also varied: timing from three days[27] to three months[16]; and extent from to within 50% of baseline[21] to within 10% of baseline[15], or below a pre-AKI value[25].

For peer review only - http://bmjopen.bmj.com/site/about/guidelines.xhtml

Table 1- Description and Application of Quality Criteria 4-6 in Studies Satisfying Criteria 1-3

			Mortality outcomes reported		CKD outcomes reported		For either outcome
Paper	N	Clinical Setting	by Pre-AKI baseline	by Post-AKI recovery	by Pre-AKI function	by Post-AKI recovery	Timing considered
Hoste 2011[28]	82	ITU	N	N	-	-	-
Lopes 2010[29]	61	ITU	N	N	-	-	-
Manns 2003[30]	66	ITU	N	N	-	-	-
Schiffl 2008[12]	226	ITU	CKD excluded	Y	-	-	-
Triverio 2009[18]	95	ITU	Y	Y	-	-	-
Bucaloiu 2012[13]	1610	Unselected	CKD excluded	Recovered only	CKD excluded	Recovered only	-
Hsu 2009[14]	782	Unselected	CKD only	N	N	N	-
Ishani 2009[19]	7197	Unselected	Y by code alone	N	Y by code alone	N	-
Jones 2012[15]	719	Unselected	CKD excluded	Recovered only	CKD excluded	Recovered only	-
Lafrance 2010[20]	82711	Unselected	Y	Y	-	-	Sensitivity analysis excluding first six months
Lo 2009[31]	343	Unselected	N (excluded only if eGFR<45)	N	-	-	-
Ng 2011[32]	262	Unselected	N	N	-	-	-
Ponte 2008[22]	177	Unselected	N (Cr>1.4 excluded)	Y	-	-	-
Wald 2012[33]	41327	Unselected	N	N	N	N	-
Gupta 2005[34]	143	Cardiac	N	N	-	-	-
Kimura 2010[35]	81	Cardiac	N	N	-	-	-
Lindsay 2003[36]	179	Cardiac	Y	N	-	-	-
Maioli 2012[16]	167	Cardiac	CKD only	Y	-	-	-
Rihal 2002[37]	185	Cardiac	N	N	-	-	-
Roghi 2008[38]	106	Cardiac	N	N	-	-	-
Brown 2010[23]	1886	Postoperative	N	Y	-	-	-
Coca 2010[24]	6257	Postoperative	N	Y	-	-	-
Ishani 2011[9]	4053*	Postoperative	N	N	N	N	Changing hazard ratios for mortality and CKD outcomes in time intervals
Kheterpal 2007[17]	101	Postoperative	CKD excluded	N	-	-	-
Loef 2005[25]	145	Postoperative	N	Y	-	-	-
Luckraz 2005[39]	53	Postoperative	N	N	-	-	-
Mehta 2010[26]	2083	Postoperative	N (excluded only Cr>2)	Y	-	-	-
Swaminathan 2010[40]	1113	Postoperative	N	N[$]	-	-	-
Van Kuijk 2010[27]	120	Postoperative	N	N	CKD excluded	Y	-
Wu 2011[21]	4393	Postoperative	Y by GFR>/<45	Y	Y by GFR>/<45	Y	-

N = outcome addressed but not per quality criterion, - = outcome not addressed by study, * = 19779 in total, 4053 of which had >50% cr rise, [$] = outcomes by rate of fall of creatinine over a 24 hours rather than a recovery variable, Cr = creatinine

For peer review only - http://bmjopen.bmj.com/site/about/guidelines.xhtml

Table 2 –Studies of Mortality Outcomes in AKI by pre and post-AKI Kidney Function

Mortality	N	Clinical Setting	Design	AKI Exposure	Comparator	Follow Up (*mean /median)	Pre-AKI baseline separation	Post-AKI recovery separation	Recovery definition	Findings (*statistically significant p<0.05)
Schiffl 2008 [12]	226	ITU	Cohort	Acute RRT	None	5 years	CKD excluded	Y	Within 10% baseline	Mortality 83% without recovery, 33% with recovery<br>HR without vs with recovery 4.1* (no non-AKI comparator)
Triverio 2009 [18]	95	ITU	Cohort	Acute RRT	None	3 years	Y	Y	eGFR>60	Mortality 50% if baseline CKD, 29% if eGFR<60 at discharge, 18% if no renal impairment before or after AKI
Bucaloiu 2012 [13]	1610	Unselected	Cohort	Cr rise >50%	No AKI	*3.3 years	CKD excluded	Recovered only	Within 10% baseline	HR Mortality 1.48*
Hsu 2009 [14]	782	Unselected	Cohort	Acute RRT	CKD with no RRT	4 years	CKD only (eGFR<45)	N	-	HR composite endpoint of ESKD or Mortality 1.3*
Ishani 2009 [19]	7197	Unselected	Cohort	Code	No AKI or CKD code	2 years	Y by code alone	N	-	HR Mortality vs no AKI or CKD<br>AKI and CKD 3.24*, AKI 2.48*, CKD 1.45*
Jones 2012 [15]	719	Unselected	Cohort	Code	No AKI	*2.5 years	CKD excluded	Recovered only	Within 10% Baseline	HR Mortality 1.08
Lafrance 2010 [20]	82711	Unselected	Cohort	Cr rise >50%	No AKI	*2.34 years	Y	Recovered only in a subanalysis	Within 10% baseline	HR Mortality for AKI vs no AKI in 90d survivors 1.41*<br>Subgroup of 6 month survivors 1.13*<br>Subgroup with recovery 1.47*
Ponte 2008 [22]	177	Unselected	Cohort	Cr rise from <1.4mg/dl to >2	None	*7.2 years	CKD excluded (Cr>1.4mg/dl)	Y	Cr <1.4mg/dl	10 year mortality 40% with recovery, 57% without recovery
Lindsay 2003 [36]	179	Cardiac	Cohort	Cr rise 50% from <1.2mg/dl	No AKI	1 year	CKD excluded (cr>1.2mg/dl)	N	-	1year mortality 9.5% AKI, 2.7% no-AKI
Maioli 2012 [16]	167	Cardiac	Cohort	Cr rise (0.5mg/dl by 3 days)	No AKI	*3.8 years	CKD only (eGFR<60)	Y	Within 25% baseline at 3 months	HR Mortality for AKI with recovery1.3*, without recovery 2.3*
Brown 2010 [23]	1886	Postoperative	Cohort	Cr rise 0.3mg/dl or 50%	No AKI	*2.6 years	N	Y	Number of days AKI definition met	HR Mortality for AKI vs no AKI by AKI Duration<br>1-2d 1.51*, 3-6d 1.74*, >7d 3.45*, persistent 5.75*
Coca 2010 [24]	6257	Postoperative	Cohort	Cr rise 0.3mg/dl or 50%	No AKI	*3.8 years	N	Y	Number of days AKI definition met	HR Mortality for AKI vs no AKI by AKI Duration<br><2d 1.15*, 3-6d 1.5*,>7d any duration with RRT 2.10*
Kheterpal 2007 [17]	101	Postoperative	Cohort	Cr clearance fall to <50	No AKI	1 year	CKD excluded	N	-	1 year mortality 12% AKI, 9% no AKI
Loef 2005 [25]	145	Postoperative	Cohort	Cr rise 25%	No AKI	100 months	N	Y	"improved to or below the preoperative level"	HR Mortality for AKI 1.63*, AKI with recovery 1.66*, AKI without recovery 1.72*, Non-recovery vs recovery 1.22 (not significant)
Mehta 2010 [26]	2083	Postoperative	Cohort	Cr rise 50% or 0.7mg/dl	No AKI	*7 years	N	Y	AKI definition no longer met at 7 days	HR mortality for AKI with recovery 1.21*, without recovery 1.47*
Wu 2011 [21]	4393	Postoperative	Cohort	Cr rise >50%	No AKI and no CKD	*4.76 years	Y by GFR>/<45	Y	Within 50% baseline	HR Mortality for AKI only 1.94*, CKD only 2.64*, AKI and CKD 3.28*<br><br>Stratified by recovery<br>AKI and CKD with recovery 3.0*, without recovery 4.59*<br>AKI only with recovery 1.96*, without recovery 2.18 *<br>CKD without AKI 2.59*

N = outcome addressed but not per quality criterion, - = outcome not addressed by study, ITU=intensive therapy unit; RRT=renal replacement therapy; AKI=acute kidney injury; CKD=chronic kidney disease; eGFR=estimated glomerular filtration rate; HR=hazard ratios; ESKD=end-stage kidney disease (dialysis>90days); Cr=serum creatinine

For peer review only - http://bmjopen.bmj.com/site/about/guidelines.xhtml

Table 3 –Studies of CKD Outcomes in AKI by pre and post-AKI Kidney Function

Progression	N	Clinical Setting	Design	AKI Exposure	Comparator	Follow-up (*mean /median)	Pre-AKI baseline separation	Post-AKI recovery separation	Recovery definition (discharge unless otherwise stated)	Findings (*statistically significant p<0.05)
Bucaloiu 2012 [13]	1610	Unselected	Cohort	Cr rise >50%	No AKI	*3.3 years	CKD excluded	Recovered only	Within 10% baseline	Development of New CKD Stage 3 per 1000 person-year<br>28.1 AKI vs 13.1 no AKI (HR 1.91*)
Ishani 2009 [19]	7197	Unselected	Cohort	Code	No AKI or CKD code	2 years	Y by code alone	N	-	ESKD/1000 person-year (HR vs no code)<br>AKI and CKD 79.45 (HR 41.2*)<br>AKI 24.52 (HR 13*)<br>CKD only 19.88 (HR 8.43*)<br>No Code 2.08 (reference)
Jones 2012 [15]	719	Unselected	Cohort	Code	No AKI	*2.5 years	CKD excluded	Recovered only	Within 10% Baseline	HR for new CKD Stage 3 (15% of AKI vs 3% of no AKI patients) 3.82*
van Kuijk 2010 [27]	493	Postoperative	Cohort	10% Cr change by day 2 post-op	No AKI	*5 years	CKD excluded	Y	Within 10% baseline at day 3	Development of New CKD<br>11% if no AKI, 32% if AKI recovered (HR 3.4*), 36% if AKI without recovery (HR3.6*)
Wu 2011 [21]	4393	Postoperative	Cohort	Cr rise (RIFLE, or 50% rise if previous CKD)	No AKI and no CKD	*4.76 years	Y by GFR>/<45	Y	Within 50% baseline	Development of ESKD (vs no code)<br>AKI only 4.64*, CKD only 40.86*, AKI and CKD 91.69*<br><br>Stratified by recovery<br>AKI + CKD + without recovery 212.73*<br>AKI + CKD + recovery 74.07*<br>AKI only + without recovery 60.95*<br>AKI only + recovery 4.5*

N = outcome addressed but not per quality criterion, - = outcome not addressed by study; ITU=intensive therapy unit; RRT=renal replacement therapy; AKI=acute kidney injury; CKD=chronic kidney disease; eGFR=estimated glomerular filtration rate; HR=hazard ratios; ESKD=end-stage renal disease (dialysis>90days); Cr=serum creatinine

For peer review only - http://bmjopen.bmj.com/site/about/guidelines.xhtml

Mortality findings
There were 16 mortality studies (table 2). Follow up ranged from 1 to 7 years and mortality up to 83% for AKI patients at 5 years. AKI was associated with increased mortality in all but one study[15] regardless of pre-AKI baseline (HR 1.08 to 4.59) (figure 3))or recovery of renal function (HR1.08 to 5.75) (figure 4). Five studies did not report non-AKI comparators[12,18,22] or risk ratios[17,36].

Of the three studies stratified by pre-AKI baseline function, in two the overall prognosis was worse in AKI patients with prior CKD with doubling of hazard ratios with respect to a non-AKI non-CKD comparator[16,21]. However, in a third study, where comparators were also stratified by eGFR, the independent mortality risk from AKI diminished with advancing CKD[20]. Six studies compared mortality stratifying either by post AKI recovery (four studies)[16,21, 25, 26] or by AKI duration (two studies)[23,24]. All but one[16] were postoperative studies with different thresholds for defining recovery. Risk from incomplete recovery varied from minimal with overlapping confidence intervals[16,21,25, 26] to greater than double when AKI lasted more than one week[23,24]. Only one study combined stratification by both pre-AKI baseline and post-AKI recovery (defining recovery to 50% baseline, CKD as eGFR<45 ml/min/1.73m^2) -with both non-recovery and baseline CKD adding to mortality risk[21].

CKD Outcomes
In five studies, there was marked variation in CKD outcomes following AKI (HR1.91 to 213) depending on pre-AKI baseline and post-AKI recovery (table 3 and figure 5)[13,15,21,19,27]. In the two studies stratifying by pre-AKI function, AKI with baseline CKD was associated with a substantially greater (four-fivefold) risk of ESKD than AKI in cases without prior CKD[19,21] although notably one study defined CKD at eGFR <45ml/min/1.73m^2 [21] and the other by a hospital code[19]. The two studies stratifying by post-AKI function had different findings. In one postoperative study[27] there was no association between non-recovery post-AKI (vs patients with recovery) and additional CKD progression, when recovery was measured at day three[27], but association was substantial (five-tenfold) in the other study where recovery was defined at hospital discharge[21]. Again only one study stratified by both baseline and recovery, finding both associated with poorer prognosis[21].

Timing of Outcomes
Two studies considered whether hazard ratios changed over time following the AKI episode[9,20]. Lafrance et al. (2010)[20], performed a sensitivity analysis of their study of AKI survivors and mortality, limiting analysis from 90 day to six month AKI survivors. The mortality HR (AKI versus non-AKI) fell from 1.41(1.39-1.43) in 90 day survivors to 1.13 (1.11-1.14) in six month survivors[20]. Ishani et al. (2011)[9] reported changes in both hazard ratio of mortality and CKD outcomes from AKI (vs no AKI) when dividing follow up into six month time discrete intervals. For both mortality and CKD outcomes, greatest relative risk (vs non-AKI) was in the first six months, with marked attenuation of risk (but still statistically significant) from one year[9]. While recognising that risk may not be constant, the study did not also consider the importance of stratifying patients by pre-AKI baseline or post-AKI recovery.

DISCUSSION
The introduction of a global definition for AKI has led to growing interest in the high incidence and poor outcomes. In this review of 7385 citations, there were only 30

For peer review only - http://bmjopen.bmj.com/site/about/guidelines.xhtml

studies that met the initial quality criteria for selecting AKI patients with sufficient efforts to avoid misclassifying CKD. Of these studies only three mortality studies stratified by pre-AKI, six by post-AKI function, two CKD outcome studies by pre-AKI and two by post AKI function. Only one study considered both pre-AKI and post-AKI function. Only two studies considered the change in risk over time from AKI.

As with two previous reviews, we note that AKI is associated with overall poorer mortality and CKD outcomes[4,6], but with substantial variation in the magnitude of risk. We were transparent about the variation in definitions and clinical setting and identified heterogeneity as a problem with the design of AKI studies to date. The two previous reviews included studies with incomplete or estimated data on baseline kidney function[4,6], but in studies with available data we found that outcomes were modulated by both pre-AKI baseline and post-AKI renal recovery. Unfortunately, these studies varied in definitions for AKI, recovery and follow up preventing pooled meta-analysis. While proteinuria and oliguria may also be important factors, research on their association with long term prognosis is sparse and we therefore focused on the course of serum creatinine prior and subsequent to the AKI episode.

Pre-AKI baseline CKD was associated with doubling of mortality outcomes and four-to five-fold increased risk of CKD outcomes compared to non-AKI non-CKD patients. Unfortunately, only one study chose CKD comparators for AKI patients at each level of pre-AKI function, finding that the additional risk from AKI diminished in those already at high risk due to advanced baseline CKD[20]. It is likely the mortality and ESKD risk in advanced CKD is already high and less influenced by AKI.

Non-recovery of kidney function was associated with greater mortality and CKD outcomes in some studies, but the finding was inconsistent and dependent on study design. Studies with a less stringent threshold[21] and later point of assessment[16] showed a poorer prognosis with non-recovery, highlighting the significance of arbitrary thresholds in study design. We also note a further study outside the time period in this review reporting a cohort with poorer outcomes with non-recovery after AKI, in line with our overall findings[41].

Increased risk of poor outcomes following AKI may be greatest early post-AKI, and therefore the timing of clinical assessment is important. Despite this, most studies followed a "proportional hazards" assumption (i.e. that the relative risk of AKI vs non-AKI comparator was constant over time), but this may not hold. Only two studies considered the timing of assessment post-AKI and found that both additional risk of mortality and renal progression may diminish over time[9,20], but neither combined this with adjustment or stratification by post-AKI recovery. Both factors are required to assess if the rate of CKD progression is more rapid in AKI than non-AKI (but baseline CKD) comparators with equal kidney function at the time of hospital discharge. Thus, understanding of how mortality and renal progression risks change over time remains inadequately addressed.

Overall while our findings broadly agree with previous reviews[4,6], we found that some of the variation in AKI prognosis may be explained by pre-AKI CKD and post-AKI non-recovery which both lead to a poorer prognosis. This is of great relevance given that these factors were infrequently addressed by stratification, and 28/61 studies had

For peer review only - http://bmjopen.bmj.com/site/about/guidelines.xhtml

insufficient pre-AKI data to minimise misclassification between CKD and AKI~~but may be a source of selection bias and misclassification of CKD patients~~. The relationships are complex with diminishing additional risk with advancing pre-AKI CKD. We also noted a non-linear clinical post-AKI course, with risk prediction dependent on the time point at which risk is assessed, necessitating fixed follow up points to prevent overestimation of risk in a cohort with shorter follow up. Importantly, few studies assessed these factors, using different definitions, and with only one study assessing both pre-AKI and post-AKI factors. This review included studies up to October 2012 and in the near future it is likely that AKI registries with additional routine outcome data will become available. Therefore there is now an opportunity to ensure that a concordance exists in definitions and reporting of future AKI studies before this review is revisited.

The NICE guidelines call for further studies of long term AKI prognosis but without specific detail on which factors should be included. We suggest that future studies should adopt consistent definitions, incorporate baseline and recovery function and consider the timing of outcomes during follow up. We therefore suggest that future long term prognostic studies should adopt the KDIGO definition for identifying and stratifying AKI[2], the KDIGO CKD stages for stratifying AKI and non-AKI comparator groups by pre-AKI eGFR[42], the UK Renal Association definition of renal recovery for stratifying recovery (within 20% of baseline)[3] and fixed interim points for establishing recovery (7, 30, 90 days) and excluding early outcomes (up to 30, 90, and 180 days).

CONCLUSION

AKI has a poor, but variable prognosis influenced by clinical setting, underlying cause and comorbidity. ~~and t~~There is a recognised need to understand which patients are at greater long-term risk and when AKI may carry additional risk beyond the underlying illness. ~~Poor prognosis may in part be explained by~~In this review, pre-AKI CKD ~~which is~~was associated with doubling of mortality outcomes and a four-fivefold increase in renal outcomes. ~~It may also be explained by non~~Non-recovery of kidney function may also affect prognosis, ~~although~~ but the magnitude ~~depends~~ depended on both recovery definition and the timing of assessment. These factors are already considered by physicians in clinical practice, ~~Both factors should be taken into account when studying AKI prognosis, and while they naturally form part of risk assessment in clinical practice,~~ but unfortunately we found that few ~~heterogenous~~ prognostic studies have addressed them. The heterogeneity of studies and accompanying lack of a clear consistent pattern of effect limit their clinical interpretation. As pre and post-AKI renal function may influence clinical practice, ~~We~~we suggest that consistent reporting of these factors is needed in future prognostic studies ~~consistently consider these prognostic factors in order~~ to ~~confirm~~ establish ~~their predictive value~~how they modify AKI prognosis. ~~and enable integration into predictive models for AKI prognosis~~This will inform both the clinician and future research.

Contributorship

SS, AM, NF and CB contributed to the design of the review, SS and MM acquired the data. SS, AM and CB contributed to analysis. SS drafted and SS, AM, NF and CB critically revised the intellectual content of the work. All authors have given final approval for this paper to be published.

For peer review only

For peer review only - http://bmjopen.bmj.com/site/about/guidelines.xhtml

Competing Interests
None declared.

Funding
SS is supported by a research training fellowship from the Wellcome Trust.

Acknowledgements
None declared.

Data Sharing
No additional data available.

REFERENCES

1. Ftouh S, Lewington A, on behalf of the Acute Kidney Injury Guideline Development Group convened by the National Clinical Guidelines Centre and commissioned by the National Institute for Health and Care Excellence,in association with The Royal College of Physicians' Clinical Effectiveness and Evaluation Unit. Prevention, detection and management of acute kidney injury: concise guideline. *Clinical Medicine* 2014, 14:61-5.
2. Kidney Disease: Improving Global Outcomes (KDIGO) Acute Kidney Injury Work Group. KDIGO Clinical Practice Guideline for Acute Kidney Injury. *Kidney Int* 2012, Suppl2:1-138.
3. Lewington A, Kanagasundaram S. Clinical Practice Guidelines. Acute Kidney Injury. 5th ed.: UK Renal Association; 2011.
4. Coca SG, Yusuf B, Shlipak MG, et al. Long-term risk of mortality and other adverse outcomes after acute kidney injury: a systematic review and meta-analysis. *Am J Kidney Dis* 2009, 53:961-973.
5. Bydash JR, Ishani A. Acute kidney injury and chronic kidney disease: a work in progress. *Clin J Am Soc Nephrol* 2011, 6:2555-2557.
6. Coca SG, Singanamala S, Parikh CR. Chronic kidney disease after acute kidney injury: a systematic review and meta-analysis. *Kidney Int* 2012, 81:442-448.
7. Chawla LS, Kimmel PL. Acute kidney injury and chronic kidney disease: an integrated clinical syndrome. *Kidney Int* 2012, 82:516-524.
8. James MT, Hemmelgarn BR, Wiebe N, et al. Glomerular filtration rate, proteinuria, and the incidence and consequences of acute kidney injury: a cohort study. *Lancet* 2010, 376:2096-2103.
9. Ishani A, Nelson D, Clothier B, et al. The magnitude of acute serum creatinine increase after cardiac surgery and the risk of chronic kidney disease, progression of kidney disease, and death. *Arch Intern Med* 2011, 171:226-233.
10. Stroup DF, Berlin JA, Morton SC, et al. Meta-analysis of observational studies in epidemiology: a proposal for reporting. Meta-analysis Of Observational Studies in Epidemiology (MOOSE) group. JAMA 2000, 283(15):2008-2012.
11. Hayden JA, Cote P, Bombardier C. Evaluation of the quality of prognosis studies in systematic reviews. *Ann Intern Med* 2006, 144:427-437.
12. Schiffl H, Fischer R. Five-year outcomes of severe acute kidney injury requiring renal replacement therapy. *Nephrol Dial Transplant* 2008, 23:2235-2241.

For peer review only

13. Bucaloiu ID, Kirchner HL, Norfolk ER, et al. Increased risk of death and de novo chronic kidney disease following reversible acute kidney injury. *Kidney Int* 2012, 81:477-485.
14. Hsu CY, Chertow GM, McCulloch CE, et al. Nonrecovery of kidney function and death after acute on chronic renal failure. *Clin J Am Soc Nephrol* 2009, 4:891-898.
15. Jones J, Holmen J, De Graauw J, et al. Association of complete recovery from acute kidney injury with incident CKD stage 3 and all-cause mortality. *Am J Kidney Dis* 2012, 60:402-408.
16. Maioli M, Toso A, Leoncini M, et al. Persistent renal damage after contrast-induced acute kidney injury: incidence, evolution, risk factors, and prognosis. *Circulation* 2012, 125:3099-3107.
17. Kheterpal S, Tremper KK, Englesbe MJ, et al. Predictors of postoperative acute renal failure after noncardiac surgery in patients with previously normal renal function. *Anesthesiology* 2007, 107:892-902.
18. Triverio PA, Martin PY, Romand J, et al. Long-term prognosis after acute kidney injury requiring renal replacement therapy. *Nephrol Dial Transplant* 2009, 24:2186-2189.
19. Ishani A, Xue JL, Himmelfarb J, et al. Acute kidney injury increases risk of ESRD among elderly. *J Am Soc Nephrol* 2009, 20:223-228
20. Lafrance JP, Miller DR. Acute kidney injury associates with increased long-term mortality. *J Am Soc Nephrol* 2010, 21:345-352.
21. Wu VC, Huang TM, Lai CF, et al. Acute-on-chronic kidney injury at hospital discharge is associated with long-term dialysis and mortality. *Kidney Int* 2011, 80:1222-1230.
22. Ponte B, Felipe C, Muriel A, et al. Long-term functional evolution after an acute kidney injury: a 10-year study: *Nephrol Dial Transplan* 2008, 23:3859-3866.
23. Brown JR, Kramer RS, Coca SG, et al. Duration of acute kidney injury impacts long-term survival after cardiac surgery. *Ann Thorac Surg* 2010, 90:1142-1148.
24. Coca SG, King JT,Jr, Rosenthal RA, et al. The duration of postoperative acute kidney injury is an additional parameter predicting long-term survival in diabetic veterans. *Kidney Int* 2010, 78:926-933.
25. Loef BG, Epema AH, Smilde TD, et al. Immediate postoperative renal function deterioration in cardiac surgical patients predicts in-hospital mortality and long-term survival. *J Am Soc Nephrol* 2005, 16:195-200.
26. Mehta RH, Honeycutt E, Patel UD, et al. Impact of recovery of renal function on long-term mortality after coronary artery bypass grafting. *Am J Cardiol* 2010, 106:1728-1734.
27. van Kuijk JP, Flu WJ, Chonchol M, et al. Temporary perioperative decline of renal function is an independent predictor for chronic kidney disease. *Clin J Am Soc Nephrol* 2010, 5:1198-1204.
28. Hoste EA, Doom S, De Waele J, et al. Epidemiology of contrast-associated acute kidney injury in ICU patients: a retrospective cohort analysis. *Intensive Care Med* 2011, 37:1921-1931.
29. Lopes JA, Fernandes P, Jorge S, et al. Long-term risk of mortality after acute kidney injury in patients with sepsis: a contemporary analysis. *BMC Nephrology* 2010, 11:9.

For peer review only - http://bmjopen.bmj.com/site/about/guidelines.xhtml

30. Manns B, Doig CJ, Lee H, et al. Cost of acute renal failure requiring dialysis in the intensive care unit: clinical and resource implications of renal recovery. *Crit Care Med* 2003, 31:449-455.
31. Lo LJ, Go AS, Chertow GM, et al. Dialysis-requiring acute renal failure increases the risk of progressive chronic kidney disease. *Kidney Int* 2009, 76:893-899.
32. Ng KP, Chanouzas D, Fallouh B, et al. Short and long-term outcome of patients with severe acute kidney injury requiring renal replacement therapy. *QJM* 2012, 105:33-39.
33. Wald R, Quinn RR, Adhikari NK, et al. Risk of chronic dialysis and death following acute kidney injury. *Am J Med* 2012, 125:585-593.
34. Gupta R, Gurm HS, Bhatt DL, et al. Renal failure after percutaneous coronary intervention is associated with high mortality. *Catheter Cardiovasc Interv* 2005, 64:442-448.
35. Kimura T, Obi Y, Yasuda K, et al. Effects of chronic kidney disease and post-angiographic acute kidney injury on long-term prognosis after coronary artery angiography. *Nephrol Dial Transplan* 2011, 26:1838-1846.
36. Lindsay J, Apple S, Pinnow EE, et al. Percutaneous coronary intervention-associated nephropathy foreshadows increased risk of late adverse events in patients with normal baseline serum creatinine. *Catheter Cardiovasc Interv* 2003, 59:338-343.
37. Rihal CS, Textor SC, Grill DE, et al. Incidence and prognostic importance of acute renal failure after percutaneous coronary intervention. *Circulation* 2002, 105:2259-2264.
38. Roghi A, Savonitto S, Cavallini C, et al. Impact of acute renal failure following percutaneous coronary intervention on long-term mortality. *J Cardiovasc Med* 2008, 9:375-381.
39. Luckraz H, Gravenor MB, George R, et al. Long and short-term outcomes in patients requiring continuous renal replacement therapy post cardiopulmonary bypass. *Eur J Cardiothorac Surg* 2005, 27:906-909.
40. Swaminathan M, Hudson CC, Phillips-Bute BG, et al. Impact of early renal recovery on survival after cardiac surgery-associated acute kidney injury. *Ann Thorac Surg* 2010, 89:1098-1104.
41. Pannu N, James M, Hemmelgarn B, et al. Association between AKI, Recovery of Renal Function, and Long-Term Outcomes after Hospital Discharge. *Clin J Am Soc Nephrol* 2013, 8:194-202.
42. Stevens PE, Levin A, Kidney Disease: Improving Global Outcomes Chronic Kidney Disease Guideline Development Work Group Members. Evaluation and management of chronic kidney disease: synopsis of the kidney disease: improving global outcomes 2012 clinical practice guideline. Ann Intern Med 2013, 158(11):825-830.

For peer review only

For peer review only - http://bmjopen.bmj.com/site/about/guidelines.xhtml

Figure 1- Quality Assessment Criteria

Figure 2- Study Selection and Quality Assessment

Figure 3 - Mortality – By Pre-AKI Baseline

w/o rec = without recovery, with rec = with recovery, HR = hazard ratio, CI = confidence interval

Figure 4 - Mortality – By Post AKI Recovery

w/o rec = without recovery, with rec = with recovery, HR = hazard ratio, CI = confidence interval

Figure 5 - CKD Outcomes

w/o rec = without recovery, with rec = with recovery, HR = hazard ratio, CI = confidence interval

For peer review only

For peer review only - http://bmjopen.bmj.com/site/about/guidelines.xhtml

1	Was there a clear objective description for AKI and comparators?
2	Was there a pre AKI assessment of kidney function (creatinine or CKD code) to identify AKI and baseline kidney function?
3	Did the study focus specifically on AKI survivors at hospital discharge or at least 30 days for calculating outcomes?
4	Was pre-AKI baseline CKD (eGFR <60ml/min/1.73m^2) used to stratify or analyze outcomes separately against a non-AKI comparator?
5	Was post-AKI renal recovery clearly described and used to stratify or analyses outcomes separately against a non-AKI comparator?
6	Was the timing of risk prediction and outcomes considered as a potential source of variation in findings?

Figure 1- Quality Assessment Criteria
931x267mm (96 x 96 DPI)

For peer review only

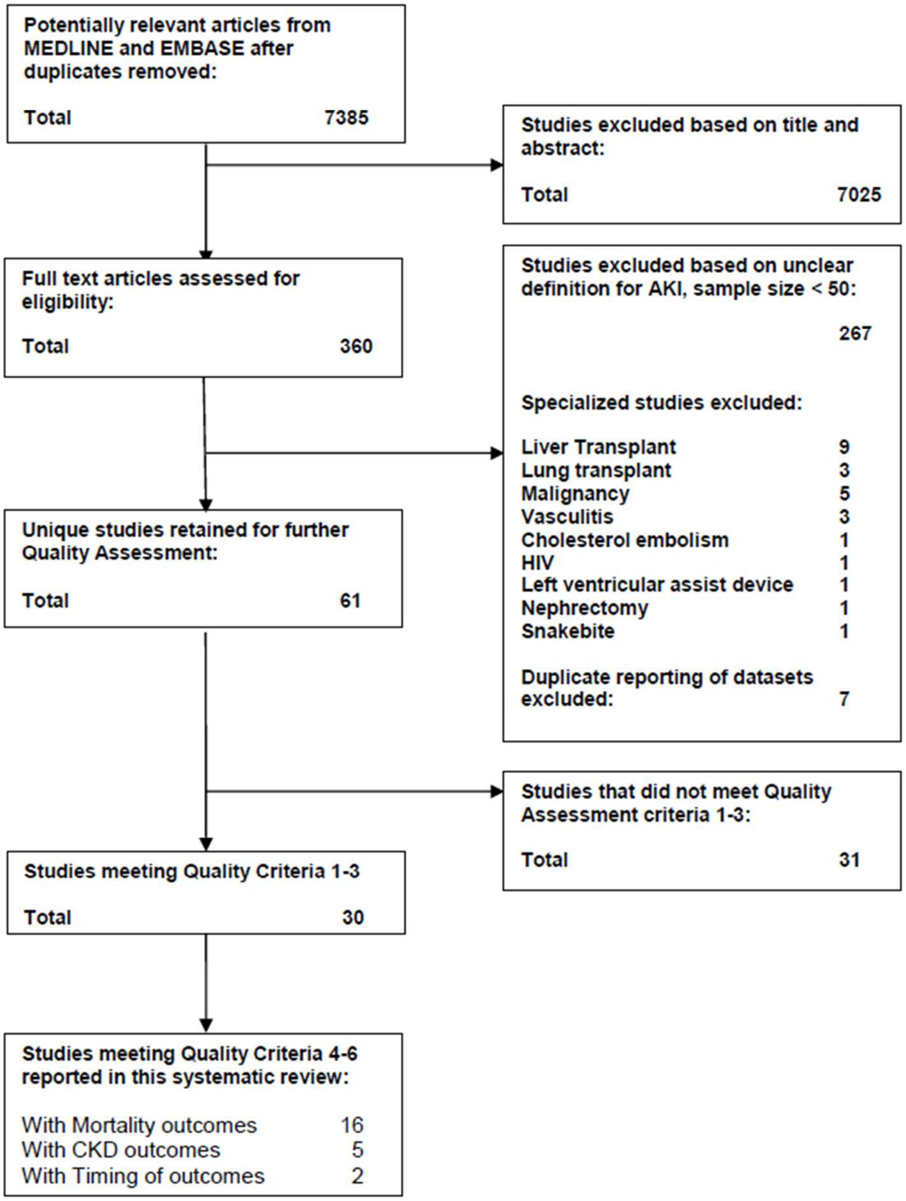

Figure 2- Study Selection and Quality Assessment
166x219mm (96 x 96 DPI)

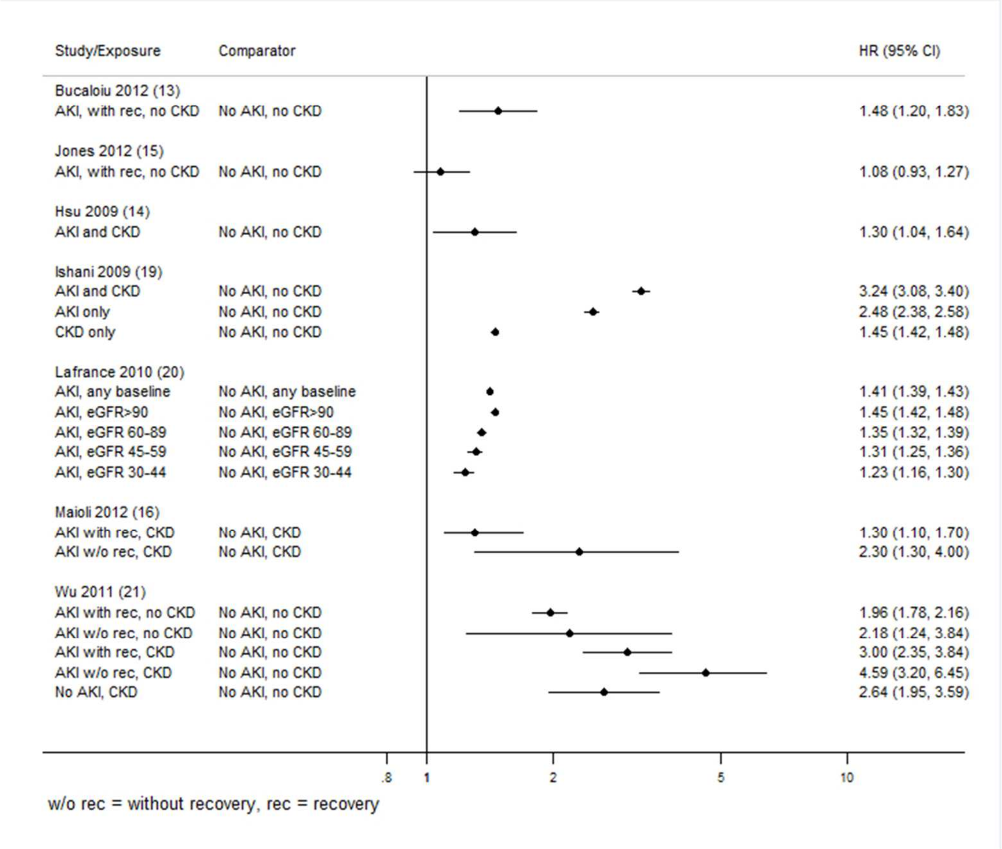

Figure 3 - Mortality – By Pre-AKI Baseline

w/o rec = without recovery, with rec = with recovery, HR = hazard ratio, CI = confidence interval
256x218mm (72 x 72 DPI)

only

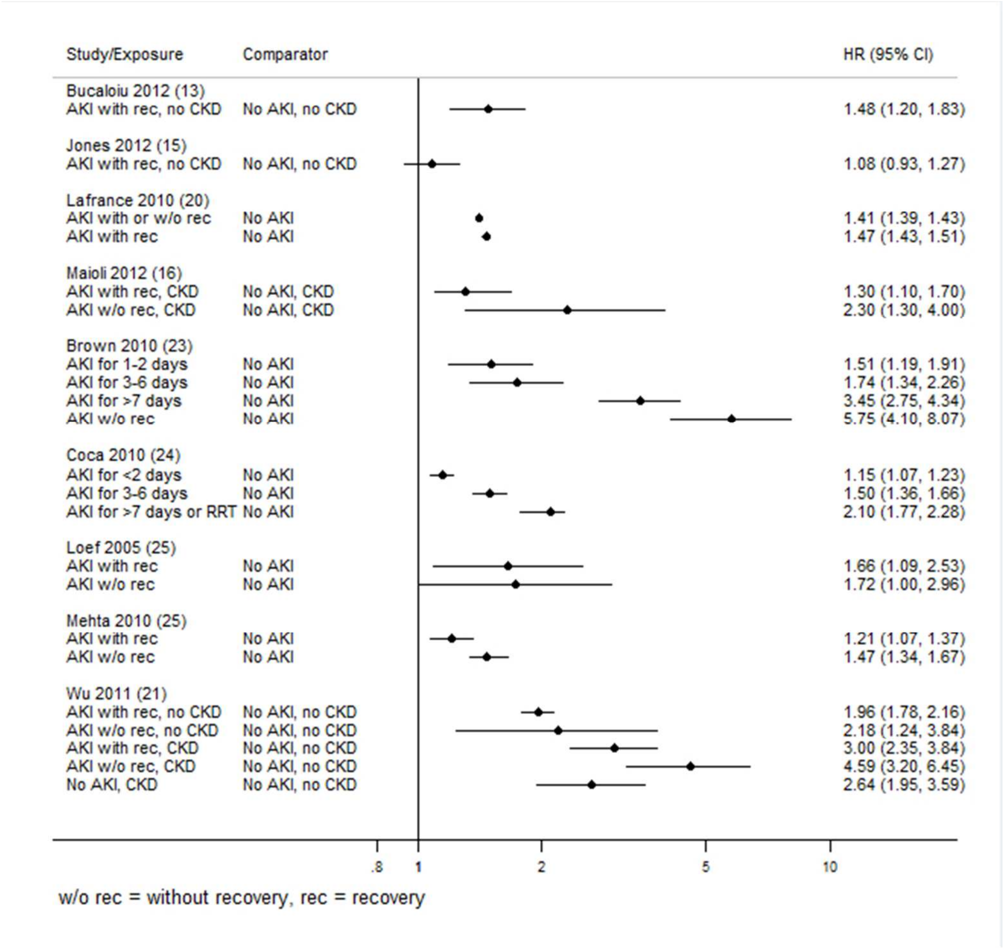

Figure 4 - Mortality – By Post AKI Recovery

w/o rec = without recovery, with rec = with recovery, HR = hazard ratio, CI = confidence interval
232x219mm (72 x 72 DPI)

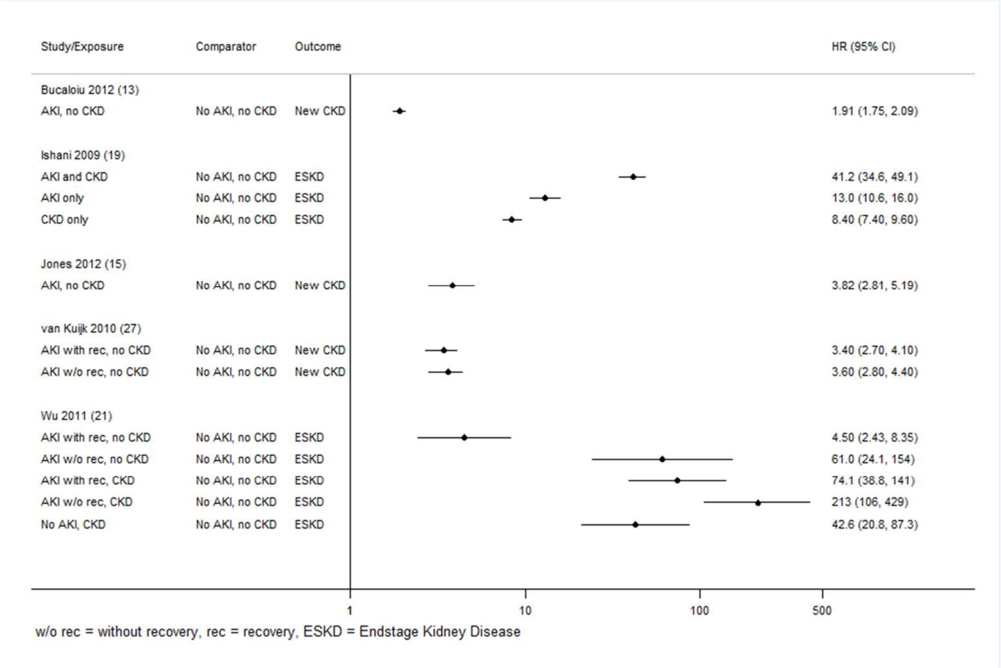

Figure 5 - CKD Outcomes

w/o rec = without recovery, with rec = with recovery, HR = hazard ratio, CI = confidence interval
327x218mm (72 x 72 DPI)

view only

Supplementary material 1 – Search strategy

1. exp Renal Insufficiency, Acute/ or exp Kidney Failure, Acute/
2. acute kidney injur$.mp. [mp=title, original title, abstract, name of substance word, subject heading word, unique identifier]
3. acute kidney failure.mp.
4. acute renal failure.mp
5. (acute adj3 dialys$).mp.
6. (acute adj3 renal replac$).mp.
7. (acute adj3 renal insufficenc$).mp.
8. exp Prognosis/
9. exp regression analysis/ or exp survival analysis/
10. exp Survival/
11. exp Mortality/
12. prognos$.mp.
13. surviv$.mp.
14. mortalit$.mp.
15. follow*up.mp.
16. recover$.mp.
17. outcome$.mp.
18. course$.mp.
19. predict$.mp.
20. (chronic adj3 dialys$).mp.
21. (chronic adj3 renal replac$).mp.
22. (chronic adj3 renal insufficienc$).mp.
23. chronic kidney disease.mp.
24. chronic renal failure.mp.
25. chronic renal disease.mp.
26. exp kidney failure, chronic/ or exp renal insufficiency, chronic/
27. exp Renal Replacement Therapy/
28. end*stage kidney.mp.
29. end*stage renal.mp.
30. end stage kidney.mp.
31. end stage renal.mp.
32. follow up.mp.
33. 8 or 9 or 10 or 11 or 12 or 13 or 14 or 15 or 16 or 17 or 18 or 19 or 20 or 21 or 22 or 23 or 24 or 25 or 26 or 27 or 28 or 29 or 30 or 31 or 32
34. 1 or 2 or 3 or 4 or 5 or 6 or 7
35. 33 and 34
36. exp adolescent/ or exp child/ or exp infant/
37. child$.mp.
38. infant.mp.
39. paediatric.mp
40. pediatric.mp.
41. case report.mp.
42. exp case reports/
43. 36 or 37 or 38 or 39 or 40 or 41 or 42
44. 35 not 43
45. limit 44 to (humans and yr="1990 -Current")
46. exp epidemiologic study characteristics as topic/
47. exp clinical conference/ or exp clinical trial/ or exp comparative study/ or exp evaluation studies/ or exp meta-analysis/ or exp multicenter study/ or exp validation studies/
48. 46 or 47
49. 45 and 48

Supplementary material 2 - Quality Assessment of Included Studies

(ITU=intensive therapy unit; RRT=renal replacement therapy; N/A=Not Applicable; Cr=serum creatinine)

Study	Clinical Context	AKI Survivors	AKI Definition	Pre-AKI baseline available	Short and Long Term Outcomes Separated	Follow Up (*mean/median available)	Quality criteria 1-3 met (clear AKI definition with pre-AKI data or hospital coding, survivor definition)
Akposso 2000 [1]	ITU	229	Cr rise	N	Y	Up to 142 months	
Bagshaw 2006 [2]	ITU	97	RRT	N	Y	Up to 1 year	
Bell 2005 [3]	ITU	112	RRT	N	Y	Up to 5 years	
Bell 2007 [4]	ITU	N/A	RRT	N	N	Up to 7 years	
Bell 2009 [5]	ITU	N/A	Cr rise	N	N	Up to 1000 days	
Carl 2010 [6]	ITU	62	RRT	N	Y	Up to 1 year	
Cartin-Ceba 2009 [7]	ITU	784	Cr rise	N	Y	Up to 6 years	
Goldberg 2009 [8]	ITU	294	Cr rise	N	Y	*36 months	
Hoste 2011 [9]	ITU	82	Cr rise	Y	Y	1 year	Y
Lins 2006 [10]	ITU	145	Cr rise	N	Y	Up 1 year	
Lopes 2010 [11]	ITU	61	Cr rise	Y	Y	*21 months	Y
Manns 2003 [12]	ITU	66	RRT	Y	Y	Up to 1 year	Y
Morgera 2002 [13]	ITU	267	RRT	N	Y	*938 days	
Schiffl 2006,2008, 2012, 2012 [14-17]	ITU	226	RRT	Y	Y	Up to 1-10 years	Y
Triverio 2009 [18]	ITU	95	RRT	Y	Y	Up to 3 years	Y

For peer review only

For peer review only - http://bmjopen.bmj.com/site/about/guidelines.xhtml

Ahlstrom 2005 [19]	Unselected	N/A	RRT	N	N	*3.9 years	
Amdur 2009, Chawla 2011 [20,21]	Unselected	5366	Code	Y	N	Up to 60 months	
Bhandari 1996 [22]	Unselected	107	RRT	N	Y	Up to 5 years	
Bucaloiu 2012 [23]	Unselected	1610	Cr rise	Y	Y	*3.3 years	Y
Frost 1993 [24]	Unselected	N/A	RRT	N	N	5 years	
Hsu 2009 [25]	Unselected	782	RRT	Y	Y	4 years	Y
Ishani 2009 [26]	Unselected	7197	Code	Code	Y	2 years	Y
James 2010B [27]	Unselected	N/A	Code	Y	N	*35 months	
Jones 2012 [28]	Unselected	719	Code	Y	Y	*2.5 years	Y
Lafrance 2010A [29]	Unselected	82711	Cr rise	Y	Y	*2.34 years	Y
Lafrance 2010B [30]	Unselected	N/A	Cr rise	Y	N	*19.4 months	
Lee 2011 [31]	Unselected	131	RRT	Y	Y	5 years	
Liano 2007, Ponte 2008 [32,33]	Unselected	177	Cr rise	Y	Y	*7.2 years	Y
Lo 2009 [34]	Unselected	343	RRT	Y	Y	10344 person-yrs	Y
Murugan 2010 [35]	Unselected	561	Cr rise	Estimated baselines	Y	1 year	
Ng 2011 [36]	Unselected	262	RRT	Y	Y	1 year	Y
Thakar 2011 [37]	Unselected	N/A	Cr rise	N	N	*61.2 months	
Wald 2009 [38]	Unselected	3769	RRT	N	Y	*3 years	
Wald 2012 [39]	Unselected	41327	Code	Code	Y	*2 years	Y
Amin 2010 [40]	Cardiac	393	Cr rise	N	Y	4 year	
Gupta 2005 [41]	Cardiac	143	Cr rise	Y	Y	*38 months	Y
Hwang 2011 [42]	Cardiac	N/A	Cr rise	N	N	1 year	
James 2010A, James 2011 [43,44]	Cardiac	553	Cr rise	Y	N	*19.7 months	
Kim 2011 [45]	Cardiac	74	Cr rise	N	Y	*358 days	
Kimura 2010 [46]	Cardiac	81	Cr rise	Y	Y	*3.8 years	Y
Lindsay 2003 [47]	Cardiac	179	Cr rise	Y	Y	1 year	Y
Maioli 2012 [48]	Cardiac	167	Cr rise	Y	Y	*3.8 years	Y
Newsome 2008 [49]	Cardiac	18069	Cr rise	N	Y	*4.1 years	
Rihal 2002 [50]	Cardiac	185	Cr rise	Y	Y	5 years	Y
Roghi 2008 [51]	Cardiac	106	Cr rise	Y	Y	2 years	Y
Bihorac 2009 [52]	Postoperative	3326	Cr rise	Estimated baselines	Y	>10 years	
Brown 2010 [53]	Postoperative	N/A	Cr rise	Y	Y	*2.6 years	Y
Coca 2010 [54,55]	Postoperative	6257	Cr rise	Y	Y	*3.8 years	Y
Hein 2006 [56]	Postoperative	148	RRT	N	Y	3 years	
Hobson 2009 [57]	Postoperative	861	Cr rise	Estimated baselines	Y	10 years	
Ishani 2011 [58]	Postoperative	4053	Cr rise	Y	Y	*5.1 years	Y
Jafari 2010 [59]	Postoperative	94	Cr rise	N	Y	1 year	
Kheterpal 2007[60]	Postoperative	101	Cr rise	Y	Y	1 year	Y
Loef 2005 [61]	Postoperative	145	Cr rise	Y	Y	100 months	Y
Luckraz 2005 [62]	Postoperative	53	RRT	Y	Y	5 years	Y
Mehta 2010 [63]	Postoperative	2083	Cr rise	Y	Y	*7 years	Y
Swaminathan 2010 [64]	Postoperative	1113	Cr rise	Y	Y	135 months	Y
van Kuijk 2010 [65]	Postoperative	493	Cr rise	Y	Y	*5 years	Y
van Kuijk 2011 [66]	Postoperative	N/A	Cr rise	Y	N	*2.2 years	
Welten 2007 [67]	Postoperative	384	Cr rise	Y	N	*6 years	
Wu 2011 [68]	Postoperative	4393	Cr rise	Y	Y	*4.76 years	Y

1. Akposso K, Hertig A, Couprie R, Flahaut A, Alberti C, Karras GA, Haymann JP, Costa De Beauregard MA, Lahlou A, Rondeau E, Sraer JD: Acute renal failure in patients over 80 years old: 25-years' experience. *Intensive Care Med* 2000, 26:400-406.
2. Bagshaw SM, Mortis G, Doig CJ, Godinez-Luna T, Fick GH, Laupland KB: One-year mortality in critically ill patients by severity of kidney dysfunction: a population-based assessment. *Am J Kidney Dis* 2006, 48:402-409.
3. Bell M, Liljestam E, Granath F, Fryckstedt J, Ekbom A, Martling CR: Optimal follow-up time after continuous renal replacement therapy in actual renal failure patients stratified with the RIFLE criteria. *Nephrol Dial Transplant* 2005, 20:354-360.
4. Bell M, Swing, Granath F, Schon S, Ekbom A, Martling CR: Continuous renal replacement therapy is associated with less chronic renal failure than intermittent haemodialysis after acute renal failure. *Intensive Care Med* 2007, 33:773-780.
5. Bell M. Granath F. Martensson J. Lofberg E. Ekbom A. Martling CR. KING (Karolinska Intensive care Nephrology Group): Cystatin C is correlated with mortality in patients with and without acute kidney injury. *Nephrol Dial Transplant* 2009, 24:3096-3102.

For peer review only

6. Carl DE, Grossman C, Behnke M, Sessler CN, Gehr TW. Effect of timing of dialysis on mortality in critically ill, septic patients with acute renal failure. *Hemodial Int* 2010, 14:11-17.
7. Cartin-Ceba R, Haugen EN, Iscimen R, Trillo-Alvarez C, Juncos L, Gajic O: Evaluation of "Loss" and "End stage renal disease" after acute kidney injury defined by the Risk, Injury, Failure, Loss and ESRD classification in critically ill patients. *Intensive Care Med* 2000, 35:2087-2095.
8. Goldberg A, Kogan E, Hammerman H, Markiewicz W, Aronson D: The impact of transient and persistent acute kidney injury on long-term outcomes after acute myocardial infarction. *Kidney Int* 2009, 76:900-906.
9. Hoste EA, Doom S, De Waele J, Delrue LJ, Defreyne L, Benoit DD, Decruyenaere J: Epidemiology of contrast-associated acute kidney injury in ICU patients: a retrospective cohort analysis. *Intensive Care Med* 2011, 37:1921-1931.
10. Lins RL, Elseviers MM, Daelemans R: Severity scoring and mortality 1 year after acute renal failure. *Nephrol Dial Transplant* 2006, 21:1066-1068.
11. Lopes JA, Fernandes P, Jorge S, Resina C, Santos C, Pereira A, Neves J, Antunes F, Gomes da Costa A: Long-term risk of mortality after acute kidney injury in patients with sepsis: a contemporary analysis. *BMC Nephrology* 2010, 11:9.
12. Manns B, Doig CJ, Lee H, Dean S, Tonelli M, Johnson D, Donaldson C: Cost of acute renal failure requiring dialysis in the intensive care unit: clinical and resource implications of renal recovery. *Crit Care Med* 2003, 31:449-455.
13. Morgera S, Kraft AK, Siebert G, Luft FC, Neumayer HH: Long-term outcomes in acute renal failure patients treated with continuous renal replacement therapies. *Am J Kidney Dis* 2002, 40:275-279.
14. Schiffl H: Renal recovery from acute tubular necrosis requiring renal replacement therapy: a prospective study in critically ill patients. *Nephrol Dial Transplant* 2006, 21:1248-1252.
15. Schiffl H, Fischer R: Five-year outcomes of severe acute kidney injury requiring renal replacement therapy. *Nephrol Dial Transplant* 2008, 23:2235-2241.
16. Schiffl H, Fischer R: Clinical cause of presumed acute tubular necrosis requiring renal replacement therapy and outcome of critically ill patients: post hoc analysis of a prospective 7-year cohort study. *Int Urol Nephrol 2012*, 44:1779-1789.
17. Schiffl H, Lang SM, Fischer R: Long-term outcomes of survivors of ICU acute kidney injury requiring renal replacement therapy: a 10-year prospective cohort study. *Clin Kidney J* 2012, 5:297-302.
18. Triverio PA, Martin PY, Romand J, Pugin J, Perneger T, Saudan P: Long-term prognosis after acute kidney injury requiring renal replacement therapy. *Nephrol Dial Transplant*, 24:2186-2189.
19. Ahlstrom A, Tallgren M, Peltonen S, Rasanen P, Pettila V: Survival and quality of life of patients requiring acute renal replacement therapy. *Intensive Care Med* 2005, 31:1222-1228.
20. Amdur RL, Chawla LS, Amodeo S, Kimmel PL, Palant CE: Outcomes following diagnosis of acute renal failure in U.S. veterans: focus on acute tubular necrosis. *Kidney Int* 2009, 76:1089-1097.
21. Chawla LS, Amdur RL, Amodeo S, Kimmel PL, Palant CE The severity of acute kidney injury predicts progression to chronic kidney disease. *Kidney Int* 2011, 79:1361-1369.
22. Bhandari S, Turney JH: Survivors of acute renal failure who do not recover renal function. *QJM* 1996, 89:415-421.

For peer review only

23. Bucaloiu ID, Kirchner HL, Norfolk ER, Hartle JE,2nd, Perkins RM: Increased risk of death and de novo chronic kidney disease following reversible acute kidney injury. *Kidney Int* 2012, 81:477-485.
24. Frost L, Pedersen RS, Bentzen S, Bille H, Hansen HE: Short and long term outcome in a consecutive series of 419 patients with acute dialysis-requiring renal failure. *Scand J Urol* 199, ;27:453-462.
25. Hsu CY, Chertow GM, McCulloch CE, Fan D, Ordonez JD, Go AS: Nonrecovery of kidney function and death after acute on chronic renal failure. *Clin J Am Soc Nephrol* 2009, 4:891-898.
26. Ishani A, Xue JL, Himmelfarb J, Eggers PW, Kimmel PL, Molitoris BA, Collins AJ: Acute kidney injury increases risk of ESRD among elderly. *J Am Soc Nephrol* 2009, 20:223-228.
27. James MT, Hemmelgarn BR, Wiebe N, Pannu N, Manns BJ, Klarenbach SW, Tonelli M, Alberta Kidney Disease N: Glomerular filtration rate, proteinuria, and the incidence and consequences of acute kidney injury: a cohort study. *Lancet* 2010, 376:2096-2103.
28. Jones J, Holmen J, De Graauw J, Jovanovich A, Thornton S, Chonchol M: Association of complete recovery from acute kidney injury with incident CKD stage 3 and all-cause mortality. *Am J Kidney Dis* 2012, 60:402-408.
29. Lafrance JP, Miller D:. Acute kidney injury associates with increased long-term mortality. *J Am Soc Nephrol* 2010, 21:345-352.
30. Lafrance JP, Djurdjev O, Levin A: Incidence and outcomes of acute kidney injury in a referred chronic kidney disease cohort. *Nephrol Dial Transplant* 2010, 25:2203-2209.
31. Lee PH, Wu VC, Hu FC, Lai CF, Chen YM, Tsai TJ, Wu KD, National Taiwan University Study Group on Acute Renal Failure: Outcomes following dialysis for acute kidney injury among different stages of chronic kidney disease. *Am J Nephrol* 2011, 34:95-103.
32. Liano F, Felipe C, Tenorio MT, Rivera M, Abraira V, Saez-de-Urturi JM, Ocana J, Fuentes C, Severiano S: Long-term outcome of acute tubular necrosis: a contribution to its natural history. *Kidney Int* 2007, 71:679-686.
33. Ponte B, Felipe C, Muriel A, Tenorio MT, Liano F: Long-term functional evolution after an acute kidney injury: a 10-year study. *Nephrol Dial Transplant* 2008, 23:3859-3866.
34. Lo LJ, Go AS, Chertow GM, McCulloch CE, Fan D, Ordonez JD, Hsu CY: Dialysis-requiring acute renal failure increases the risk of progressive chronic kidney disease. *Kidney Int* 2009, 76:893-899.
35. Murugan R, Kellum JA: Acute kidney injury: what's the prognosis? *Nat Rev Nephrol* 2011, 7:209-217.
36. Ng KP, Chanouzas D, Fallouh B, Baharani J: Short and long-term outcome of patients with severe acute kidney injury requiring renal replacement therapy. *QJM* 2012, 105:33-39.
37. Thakar CV, Christianson A, Himmelfarb J, Leonard AC: Acute kidney injury episodes and chronic kidney disease risk in diabetes mellitus. *Clin J Am Soc Nephrol* 2011, 6:2567-2572.
38. Wald R, Quinn RR, Luo J, Li P, Scales DC, Mamdani MM, Ray JG, University of Toronto Acute Kidney Injury Research,Group: Chronic dialysis and death among survivors of acute kidney injury requiring dialysis. *JAMA* 2009, 302:1179-1185.
39. Wald R, Quinn RR, Adhikari NK, Burns KE, Friedrich JO, Garg AX, Harel Z, Hladunewich MA, Luo J, Mamdani M, Perl J, Ray JG, University of Toronto Acute Kidney Injury Research,Group: Risk of chronic dialysis and death following acute kidney injury. *Am J Med* 2012, 125:585-593.

For peer review only

40. Amin AP, Spertus JA, Reid KJ, Lan X, Buchanan DM, Decker C, Masoudi FA: The prognostic importance of worsening renal function during an acute myocardial infarction on long-term mortality. *Am Heart J* 2010, 160:1065-1071.
41. Gupta R, Gurm HS, Bhatt DL, Chew DP, Ellis SG: Renal failure after percutaneous coronary intervention is associated with high mortality. *Catheter Cardiovasc Interv* 2005, 64:442-448.
42. Hwang SH, Jeong MH, Ahmed K, Kim MC, Cho KH, Lee MG, Ko JS, Park KH, Sim DS, Yoon NS, Yoon HJ, Kim KH, Hong YJ, Park HW, Kim JH, Ahn YK, Cho JG, Park JC, Kang JC: Different clinical outcomes of acute kidney injury according to acute kidney injury network criteria in patients between ST elevation and non-ST elevation myocardial infarction. *Int J Cardiol* 2011, 150:99-101.
43. James MT, Ghali WA, Tonelli M, Faris P, Knudtson ML, Pannu N, Klarenbach SW, Manns BJ, Hemmelgarn BR: Acute kidney injury following coronary angiography is associated with a long-term decline in kidney function. *Kidney Int* 2010, 78:803-809.
44. James MT. Ghali WA. Knudtson ML. Ravani P. Tonelli M. Faris P. Pannu N. Manns BJ. Klarenbach SW. Hemmelgarn BR. Alberta Provincial Project for Outcome Assessment in Coronary Heart Disease (APPROACH) Investigators: Associations between acute kidney injury and cardiovascular and renal outcomes after coronary angiography. *Circulation* 2011, 123:409-416.
45. Kim MJ, Choi HS, Oh SH, Lee HC, Kim CS, Choi JS, Klarenbach SW, Manns BJ, Hemmelgarn BR: Acute kidney injury following coronary angiography is associated with a long-term decline in kidney function. *Yonsei Med J* 2011, 52:603-609.
46. Kimura T, Obi Y, Yasuda K, Sasaki K, Takeda Y, Nagai Y, Imai E, Rakugi H, Isaka Y, Hayashi T: Effects of chronic kidney disease and post-angiographic acute kidney injury on long-term prognosis after coronary artery angiography. *Nephrol Dial Transplan* 2011, 26:1838-1846.
47. Lindsay J, Apple S, Pinnow EE, Gevorkian N, Gruberg L, Satler LF, Pichard AD, Kent KM, Suddath W, Waksman R: Percutaneous coronary intervention-associated nephropathy foreshadows increased risk of late adverse events in patients with normal baseline serum creatinine. *Catheter Cardiovasc Interv* 2003, 59:338-343.
48. Maioli M, Toso A, Leoncini M, Gallopin M, Musilli N, Bellandi F: Persistent renal damage after contrast-induced acute kidney injury: incidence, evolution, risk factors, and prognosis. *Circulation* 2012, 125:3099-3107.
49. Newsome BB, Warnock DG, McClellan WM, Herzog CA, Kiefe CI, Eggers PW, Allison JJ: Long-term risk of mortality and end-stage renal disease among the elderly after small increases in serum creatinine level during hospitalization for acute myocardial infarction. *Arch Intern Med* 2008, 168:609-616.
50. Rihal CS, Textor SC, Grill DE, Berger PB, Ting HH, Best PJ, Singh M, Bell MR, Barsness GW, Mathew V, Garratt KN, Holmes DR,Jr: Incidence and prognostic importance of acute renal failure after percutaneous coronary intervention. *Circulation* 2002, 105:2259-2264.
51. Roghi A. Savonitto S. Cavallini C. Arraiz G. Angoli L. Castriota F. Bernardi G. Sansa M. De Servi S. Pitscheider W. Danzi GB. Reimers B. Klugmann S. Zaninotto M. Ardissino D. Atherosclerosis, Thrombosis and Vascular Biology Study Group and the Italian Society for Invasive Cardiology Investigators: Impact of acute renal failure following percutaneous coronary intervention on long-term mortality. *J Cardiovasc Med* 2008, 9:375-381.
52. Bihorac A, Yavas S, Subbiah S, Hobson CE, Schold JD, Gabrielli A, Layon AJ, Segal MS: Long-term risk of mortality and acute kidney injury during hospitalization after major surgery. *Ann Surg* 2009, 249:851-858.

For peer review only

53. Brown JR, Kramer RS, Coca SG, Parikh CR: Duration of acute kidney injury impacts long-term survival after cardiac surgery. *Ann Thorac Surg* 2010, 90:1142-1148.
54. Coca SG, King JT,Jr, Rosenthal RA, Perkal MF, Parikh CR: The duration of postoperative acute kidney injury is an additional parameter predicting long-term survival in diabetic veterans. *Kidney Int* 2010, 78:926-933.
55. Coca SG: Long-term outcomes of acute kidney injury. *Curr Opin Nephrol Hypertens* 2010, 19:266-272.
56. Hein OV, Birnbaum J, Wernecke KD, Konertz W, Jain U, Spies C: Three-year survival after four major post-cardiac operative complications. *Crit Care Med* 2006, 34:2729-2737.
57. Hobson CE, Yavas S, Segal MS, Schold JD, Tribble CG, Layon AJ, Bihorac A: Acute kidney injury is associated with increased long-term mortality after cardiothoracic surgery. *Circulation* 2009, 119:2444-2453.
58. Ishani A, Nelson D, Clothier B, Schult T, Nugent S, Greer N, Slinin Y, Ensrud KE: The magnitude of acute serum creatinine increase after cardiac surgery and the risk of chronic kidney disease, progression of kidney disease, and death. *Arch Intern Med* 2011, 171:226-233.
59. Jafari SM, Huang R, Joshi A, Parvizi J, Hozack WJ: Renal impairment following total joint arthroplasty: who is at risk? *J Arthroplasty* 2010, 25:49; Se-53.
60. Kheterpal S, Tremper KK, Englesbe MJ, O'Reilly M, Shanks AM, Fetterman DM, Rosenberg AL, Swartz RD: Predictors of postoperative acute renal failure after noncardiac surgery in patients with previously normal renal function. *Anesthesiology* 2007, 107:892-902.
61. Loef BG, Epema AH, Smilde TD, Henning RH, Ebels T, Navis G, Stegeman CA: Immediate postoperative renal function deterioration in cardiac surgical patients predicts in-hospital mortality and long-term survival. *J Am Soc Nephrol* 2005, 16:195-200.
62. Luckraz H, Gravenor MB, George R, Taylor S, Williams A, Ashraf S, Argano V, Youhana A: Long and short-term outcomes in patients requiring continuous renal replacement therapy post cardiopulmonary bypass. *Eur J Cardiothorac Surg* 2005, 27:906-909.
63. Mehta RH, Honeycutt E, Patel UD, Lopes RD, Shaw LK, Glower DD, Harrington RA, Califf RM, Sketch MH,Jr: Impact of recovery of renal function on long-term mortality after coronary artery bypass grafting. *Am J Cardiol* 2010, 106:1728-1734.
64. Swaminathan M, Hudson CC, Phillips-Bute BG, Patel UD, Mathew JP, Newman MF, Milano CA, Shaw AD, Stafford-Smith M: Impact of early renal recovery on survival after cardiac surgery-associated acute kidney injury. *Ann Thorac Surg* 2010, 89:1098-1104.
65. van Kuijk JP, Flu WJ, Chonchol M, Hoeks SE, Winkel TA, Verhagen HJ, Bax JJ, Poldermans D: Temporary perioperative decline of renal function is an independent predictor for chronic kidney disease. *Clin J Am Soc Nephrol* 2010, 5:1198-1204.
66. van Kuijk JP, Flu WJ, Valentijn TM, Chonchol M, Voute MT, Kuiper RJ, Verhagen HJ, Bax JJ, Poldermans D: Preoperative left ventricular dysfunction predisposes to postoperative acute kidney injury and long-term mortality. *J Nephrol* 2011, 24:764-770.
67. Welten GM, Schouten O, Chonchol M, Hoeks SE, Feringa HH, Bax JJ, Dunkelgrun M, van Gestel YR, van Domburg RT, Poldermans D: Temporary worsening of renal function after aortic surgery is associated with higher long-term mortality. *Am J Kidney Dis* 2007, 50:219-228.
68. Wu VC, Huang TM, Lai CF, Shiao CC, Lin YF, Chu TS, Wu PC, Chao CT, Wang JY, Kao TW, Young GH, Tsai PR, Tsai HB, Wang CL, Wu MS, Chiang WC, Tsai IJ, Hu FC, Lin SL, Chen YM, Tsai TJ, Ko WJ, Wu KD: Acute-on-chronic kidney injury at

For peer review only

hospital discharge is associated with long-term dialysis and mortality. *Kidney Int* 2011, 80:1222-1230.

For peer review only

PRISMA 2009 Checklist

Section/topic	#	Checklist item	Reported on page #
**TITLE**			
Title	1	Identify the report as a systematic review, meta-analysis, or both.	p1
**ABSTRACT**			
Structured summary	2	Provide a structured summary including, as applicable: background; objectives; data sources; study eligibility criteria, participants, and interventions; study appraisal and synthesis methods; results; limitations; conclusions and implications of key findings; systematic review registration number.	p2
**INTRODUCTION**			
Rationale	3	Describe the rationale for the review in the context of what is already known.	p4
Objectives	4	Provide an explicit statement of questions being addressed with reference to participants, interventions, comparisons, outcomes, and study design (PICOS).	p4
**METHODS**			
Protocol and registration	5	Indicate if a review protocol exists, if and where it can be accessed (e.g., Web address), and, if available, provide registration information including registration number.	n/a
Eligibility criteria	6	Specify study characteristics (e.g., PICOS, length of follow-up) and report characteristics (e.g., years considered, language, publication status) used as criteria for eligibility, giving rationale.	pp4-5
Information sources	7	Describe all information sources (e.g., databases with dates of coverage, contact with study authors to identify additional studies) in the search and date last searched.	pp4-5
Search	8	Present full electronic search strategy for at least one database, including any limits used, such that it could be repeated.	Supplementary material
Study selection	9	State the process for selecting studies (i.e., screening, eligibility, included in systematic review, and, if applicable, included in the meta-analysis).	pp4-5, Figure 2
Data collection process	10	Describe method of data extraction from reports (e.g., piloted forms, independently, in duplicate) and any processes for obtaining and confirming data from investigators.	p5
Data items	11	List and define all variables for which data were sought (e.g., PICOS, funding sources) and any assumptions and simplifications made.	p5
Risk of bias in individual studies	12	Describe methods used for assessing risk of bias of individual studies (including specification of whether this was done at the study or outcome level), and how this information is to be used in any data synthesis.	p5, Figure 1
Summary measures	13	State the principal summary measures (e.g., risk ratio, difference in means).	pp5-6
Synthesis of results	14	Describe the methods of handling data and combining results of studies, if done, including measures of consistency (e.g., I^2) for each meta-analysis.	pp5-6

For peer review only - http://bmjopen.bmj.com/site/about/guidelines.xhtml

PRISMA 2009 Checklist

Section/topic	#	Checklist item	Reported on page #
Risk of bias across studies	15	Specify any assessment of risk of bias that may affect the cumulative evidence (e.g., publication bias, selective reporting within studies).	n/a
Additional analyses	16	Describe methods of additional analyses (e.g., sensitivity or subgroup analyses, meta-regression), if done, indicating which were pre-specified.	n/a
**RESULTS**			
Study selection	17	Give numbers of studies screened, assessed for eligibility, and included in the review, with reasons for exclusions at each stage, ideally with a flow diagram.	p6
Study characteristics	18	For each study, present characteristics for which data were extracted (e.g., study size, PICOS, follow-up period) and provide the citations.	p6
Risk of bias within studies	19	Present data on risk of bias of each study and, if available, any outcome level assessment (see item 12).	p6, Table 1
Results of individual studies	20	For all outcomes considered (benefits or harms), present, for each study: (a) simple summary data for each intervention group (b) effect estimates and confidence intervals, ideally with a forest plot.	p6-7<br>Tables 2,3<br>Figures 3-5
Synthesis of results	21	Present results of each meta-analysis done, including confidence intervals and measures of consistency.	n/a
Risk of bias across studies	22	Present results of any assessment of risk of bias across studies (see Item 15).	n/a
Additional analysis	23	Give results of additional analyses, if done (e.g., sensitivity or subgroup analyses, meta-regression [see Item 16]).	n/a
**DISCUSSION**			
Summary of evidence	24	Summarize the main findings including the strength of evidence for each main outcome; consider their relevance to key groups (e.g., healthcare providers, users, and policy makers).	pp7-8
Limitations	25	Discuss limitations at study and outcome level (e.g., risk of bias), and at review-level (e.g., incomplete retrieval of identified research, reporting bias).	pp8
Conclusions	26	Provide a general interpretation of the results in the context of other evidence, and implications for future research.	p9
**FUNDING**			
Funding	27	Describe sources of funding for the systematic review and other support (e.g., supply of data); role of funders for the systematic review.	p9

From: Moher D, Liberati A, Tetzlaff J, Altman DG, The PRISMA Group (2009). Preferred Reporting Items for Systematic Reviews and Meta-Analyses: The PRISMA Statement. PLoS Med 6(6): e1000097. doi:10.1371/journal.pmed1000097

For more information, visit: www.prisma-statement.org

For peer review only - http://bmjopen.bmj.com/site/about/guidelines.xhtml

PRISMA 2009 Checklist

For peer review only

MOOSE Checklist

Long Term Prognosis after Acute Kidney Injury (AKI) - What is the Role of Baseline Kidney Function and Recovery? A Systematic Review.

	Reported on page	Comments
**Reporting of background should include**		
Problem definition	4	The factors associated with long-term AKI prognosis are poorly understood
Hypothesis statement	4	We seek to evaluate whether previous observational studies have demonstrated the modifying contribution of *pre-AKI* baseline function, *post-AKI* recovery and the timing of outcomes
Description of study outcomes	5	Studies had to report either mortality or a CKD outcome (development of CKD, progression of CKD severity, or end-stage kidney disease (ESKD))
Type of exposure or intervention used	5	AKI: creatinine changes within a specified time interval, hospital episode coding for AKI, or the initiation of acute renal replacement therapy (RRT)
Type of study designs used	4	Observational studies
Study population	5	Studies were included that reported adults (age over 18 years), had at least 50 participants with AKI surviving to hospital discharge, and had follow-up of at least one year.
**Reporting of search strategy should include**		
Qualifications of searchers (eg librarians and investigators)	1	Indicated in the author list
Search strategy, including time period used in the synthesis and key words	4, Supplementary material	From January 1990 to October 2012. Outlined in Methods with further detail in the supplementary material.
Databases and registries searched	4	Medline and EMBASE
Search software used, name and version, including special features used (eg explosion)	4	Medline including explosion of MeSH term
Use of hand searching (eg reference lists of obtained articles)	5	Reference lists of relevant studies and review articles were also searched.

For peer review only

List of citations located and those excluded, including justification	Figure 2, Supplementary material	A Prisma flow diagram in included, quality assessment exclusion of full texts is summarised in the supplementary material.
Method of addressing articles published in languages other than English	5	No language restrictions were placed, translation software was used to confirm relevance of screened abstracts.
Method of handling abstracts and unpublished studies		No abstracts without full text met quality criteria for inclusion in the paper
Description of any contact with authors		No additional contact with authors was made.
**Reporting of methods should include**		
Description of relevance or appropriateness of studies assembled for assessing the hypothesis to be tested	4-5, Figure 1	Inclusion and exclusion criteria are outlined in the methods, including quality assessment criteria.
Rationale for the selection and coding of data (eg sound clinical principles or convenience)	5-6	Data extracted included quality criteria, details of exposure, factors and outcomes of interest
Assessment of confounding (eg comparability of cases and controls in studies where appropriate)	5-6	Only studies with AKI definitions and pre-AKI data that would minimise misclassification of CKD were included.
Assessment of study quality, including blinding of quality assessors, stratification or regression on possible predictors of study results	5, Figure 1	Quality assessment criteria were used to exclude papers without pre-AKI data and short term AKI survivor data. Studies stratifying on pre-AKI and post-AKI function were analysed separately.
Assessment of heterogeneity	Table 1	Differences in clinical setting and AKI definitions for each study were reported.
Description of statistical methods (eg complete description of fixed or random effects models, justification of whether the chosen models account for predictors of study results, dose-response models, or cumulative meta-analysis) in sufficient detail to be replicated		Not applicable – meta-analysis was not considered appropriate
Provision of appropriate tables and graphics	Figures 1-5, Tables 1-3	1 box detailing quality criteria, 1 flow diagram of included studies, 3 forest plots of outcomes stratified by pre-AKI and post-AKI function. 1 table detailing all included studies and 2 tables of mortality and CKD

For peer review only

		outcomes.
**Reporting of results should include**		
Graphic summarizing individual study estimates and overall estimate	Figures 3-5	
Table giving descriptive information for each study included	Table 1	
Results of sensitivity testing (eg subgroup analysis)	Tables 2 and 3	
Indication of statistical uncertainty of findings	Figures 3-5	95% confidence intervals were presented for summary estimates.
**Reporting of discussion should include**		
Quantitative assessment of bias (eg publication bias)		Not performed.
Justification for exclusion (eg exclusion of non-English language citations)	11	We excluded studies that would potentially misclassify AKI as CKD resulting in selection bias, and studies that did not address pre-AKI and post-AKI factors of interest.
Assessment of quality of included studies	11, table , Supplementary material	We summarised the breakdown of included studies and quality criteria met. We also discussed variations in definitions.
**Reporting of conclusions should include**		
Consideration of alternative explanations for observed results	11	We discussed the importance of underlying clinical setting and comorbidity and potential for misclassification between AKI and CKD. We also discussed variations in disease definitions in each study resulting in different patient selections and different outcomes.
Generalization of the conclusions (eg appropriate for the data presented and within the domain of the literature review)	12	We reported a variable prognosis of AKI and variable role of pre-AKI and post-AKI function depending on disease definitions.
Guidelines for future research	12	We have recommended consistency in the reporting of pre-AKI and post-AKI factors in future studies in order to establish their influence on outcomes.
Disclosure of funding source	13	SS is supported by a research training fellowship from the Wellcome Trust.

For peer review only - http://bmjopen.bmj.com/site/about/guidelines.xhtml
